# Light-Driven Photoconversion of Squaramides with Implications in Anion Transport

**DOI:** 10.1021/acs.orglett.3c00993

**Published:** 2023-05-09

**Authors:** Manel Vega, Luis Martínez-Crespo, Miguel Barceló-Oliver, Carmen Rotger, Antonio Costa

**Affiliations:** Department of Chemistry, Universitat de les Illes Balears, Palma 07122, Spain

## Abstract

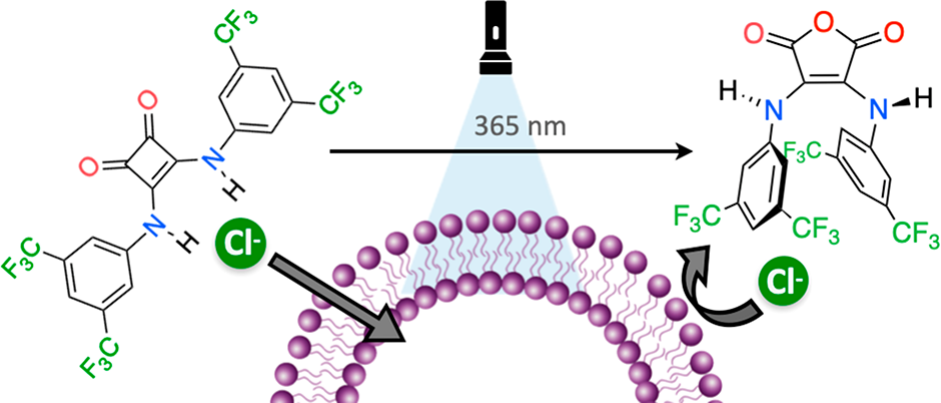

Simple, clean and fast photoconversion of aniline-derived squaramides was achieved by flashlight illumination. UV irradiation enabled the photochemical squaramide ring-opening to generate 1,2-bisketenes, which DMSO trapped as the nucleophilic oxidant. The only photoproducts isolated were 3,4-arylamino maleic anhydrides, which present conformational preferences very different from those of their parent squaramides. Similar photoconversion was achieved in MeOH. The UV-mediated time-dependent anion transport inhibition was demonstrated, establishing a new approach for modulating the transport abilities of AD-squaramides.

Aniline-derived squaramides (AD-squaramides) are a group of disubstituted squaramide derivatives with applications in organocatalysis,^1−5^ and material science.^6−8^ Moreover, AD-squaramides are well-suited materials for ion transport with potential applications in human health.^9−15^ In most cases, the observed activities of AD-squaramides are directly related to their hydrogen-bond donating abilities.^16,17^ Squaramides are chemically stable in organic solution, even in the presence of acids and bases, and in aqueous media, within a 2–8 pH range.^18^ Such stability make AD-squaramides valid for biological uses, although excessive chemical stability, if coupled with ineffective metabolic degradative pathways, can lead to unregulated accumulation and toxicity effects on cells.^13^

It is known that under the influence of UV-light irradiation, squaramides generate the corresponding aminobisketenes by electrocyclic ring-opening of the cyclobutenedione ring (Scheme 1A),^19−23^ although the thermal equilibrium is heavily displaced toward the squaramide moiety. However, despite its minority status, aminobisketenes are highly electrophilic species that can be trapped by irreversible reactions with nucleophiles such as water or alcohols, displacing the unfavorable squaramide–aminobisketene equilibrium.^19,24^

**Scheme 1:**
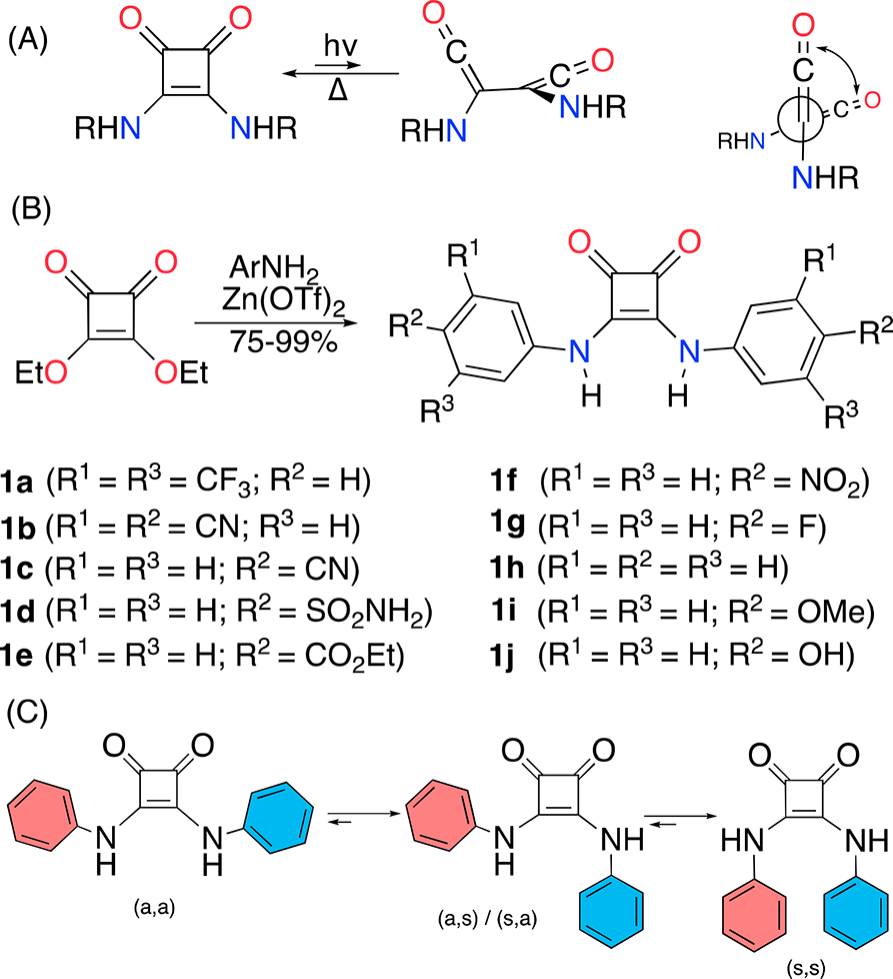
(A) Light-Driven Squaramide–1,2-Bisketene Equilibrium (Lateral and Front View). (B) Synthesis and Molecular Structures of AD-Squaramides Studied in This Work. (C) Possible Limiting Conformations of AD-Squaramides

In this work, diversely modified AD-squaramides were prepared and their light-driven photoconversion was investigated in DMSO and MeOH. Moreover, we present compelling evidence that squaramide-mediated chloride transport can be modulated or even completely inhibited by UV irradiation in bilayer membranes.

AD-squaramides **1a**–**1j** were synthesized by modifying the standard condensation method of diethyl squarate with anilines catalyzed by zinc triflate (Scheme 1).^25^ In our procedure, we changed the original toluene–DMF solvent mixture to *n*-octanol. This sustainable, high-boiling point solvent afforded in most cases the AD-squaramides in comparable yields to those reported for the same or related squaramides (Supporting Information, (SI)).

Although, the AD-squaramides could, in principle, exist in four different conformational states (Scheme 1C), namely, anti,anti (a,a) and degenerate anti,syn (a,s), syn,anti (s,a), and syn,syn (s,s), we assigned the observed set of narrow NMR peaks to the (a,a) conformer, in agreement with previous reports.^26^ The ortho hydrogens always appear downfield related to meta or para hydrogens due to the paramagnetic influence of the squaramide carbonyls. Moreover, due to hydrogen bonding with the DMSO solvent, the NHs appear strongly deshielded at 9.5–10.7 ppm.^25^ The X-ray structures of **1a** (reported),^9,25^**1b**, **1c**, and **1d** (Figures S5.1–S5.3, respectively), support the (a,a)-type conformational assignment.

Our photoconversion studies began on the NMR tube scale with a solution of **1a** (10^–3^ M) in DMSO-*d*_*6*_ at room temperature. Upon short illumination periods (seconds) with a commercial UV-LED flashlight (365 nm, 10W) we detected the time-dependent formation of a new photoproduct **2a** (Figure 1B). Clean and irreversible photoconversion of **1a** to **2a** took place in less than 30 s (Figure 1B and Figure S3.1). The peaks for the photoproduct **2a** are all upfield-shifted relative to **1a**, with the signals of the ortho hydrogens exhibiting more shielding than that of the para hydrogen, −0.81 vs −0.5 ppm, respectively. This observation, general for all photoconversions described below, is consistent with a photoproduct **2a** that is a symmetric, less hydrogen-bonded compound with the aryl rings in a cofacial (s,s)-type conformation.^27^ The ESI-HRMS analysis of **2a** exhibited a molecular mass recorded in acetone, *m*/*z* 16 units higher than the starting AD-squaramide **1a**. Based on these results, and similarly to what has been reported from 1,2-bisketenes in the presence of oxygen,^19^ we proposed an anhydride structure for the photoproduct **2a** obtained in DMSO (Figure 1).

**Figure 1:**
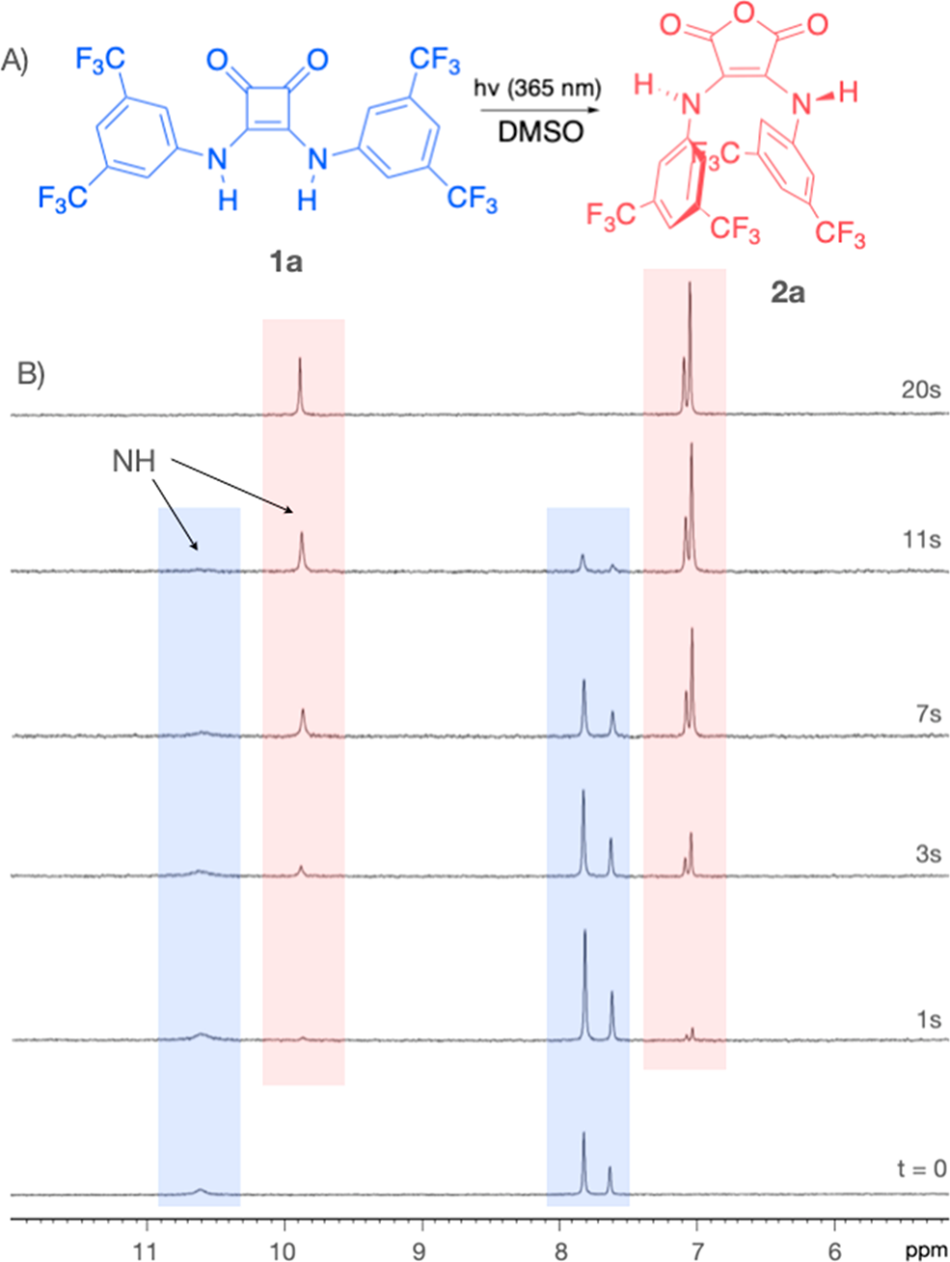
(A) Molecular structures of AD-squaramides **1** and their photoproducts **2**. (B) Time course of ^1^H NMR spectral changes observed during the photoconversion of **1a** to **2a**. The irradiation time is indicated in seconds.

Similarly, the *in situ* generated photoproducts from AD-squaramides **1b**–**1j** (10^–3^ M) were examined by ^1^H NMR (Figures S3.2–S3.10). In order to compare the different photoconversion rates, we determined the initial rates by linear fitting (*R*^2^ > 0.98) of the initial data points obtained (Table 1, Figures S3.1–S3.8). While the clean complete photoconversion of AD-squaramides **1a**–**1e** takes place in less than one minute, it lasts almost one hour for **1f** and **1h** in comparable conditions. Moreover, the electron-rich AD-squaramides **1i** and **1j** remained without apparent transformation after one hour irradiation, implying that their squaramide-aminobisketene equilibria were heavily displaced to the squaramide side without any other competitive process being involved (Figures S3.9 and S3.10).

**Table 1:** Initial Conversion Rates of AD-Squaramides **1** and Isolated Yields of Maleic Anhydride Derivatives **2**

Compd.	Init. rate/10^7^ mol dm^–3^ s^–1^	Compd.	Yield^a^/%
**1a**	810 ± 70	**2a**	76
**1b**	955 ± 120	**2b**	68
**1c**	1060 ± 15	**2c**	90
**1d**	480 ± 24	**2d**	55
**1e**	325 ± 36	**2e**	74
**1f**	0.48 ± 0.01	**2f**	-b
**1g**	0.15 ± 0.01	**2g**	-b
**1h**	0.14 ± 0.01	**2h**	-b
**1i**	n.a.	**2i**	-c
**1j**	n.a.	**2j**	-c

a Isolated yields after CC purification.

b Too slow photoconversion for practical use.

c Not observed.

Next, to reproduce the photoconversion at a preparative (0.5 mmol) scale, we used a 400 W medium-pressure UV lamp to irradiate a solution of **1a** in a THF–DMSO solvent mixture in a Schlenk tube attached to a glass photoreactor (temp. ca. 28 °C). After completion and solvent elimination, crude **2a**–**2e** was purified by column chromatography (CC) (Table 1). The anhydrides were spectroscopically characterized, and the structures of **2a** and **2c** were also confirmed by single-crystal X-ray diffraction analysis (Scheme 2 and Figures S5.4 and S5.5).

**Scheme 2:**
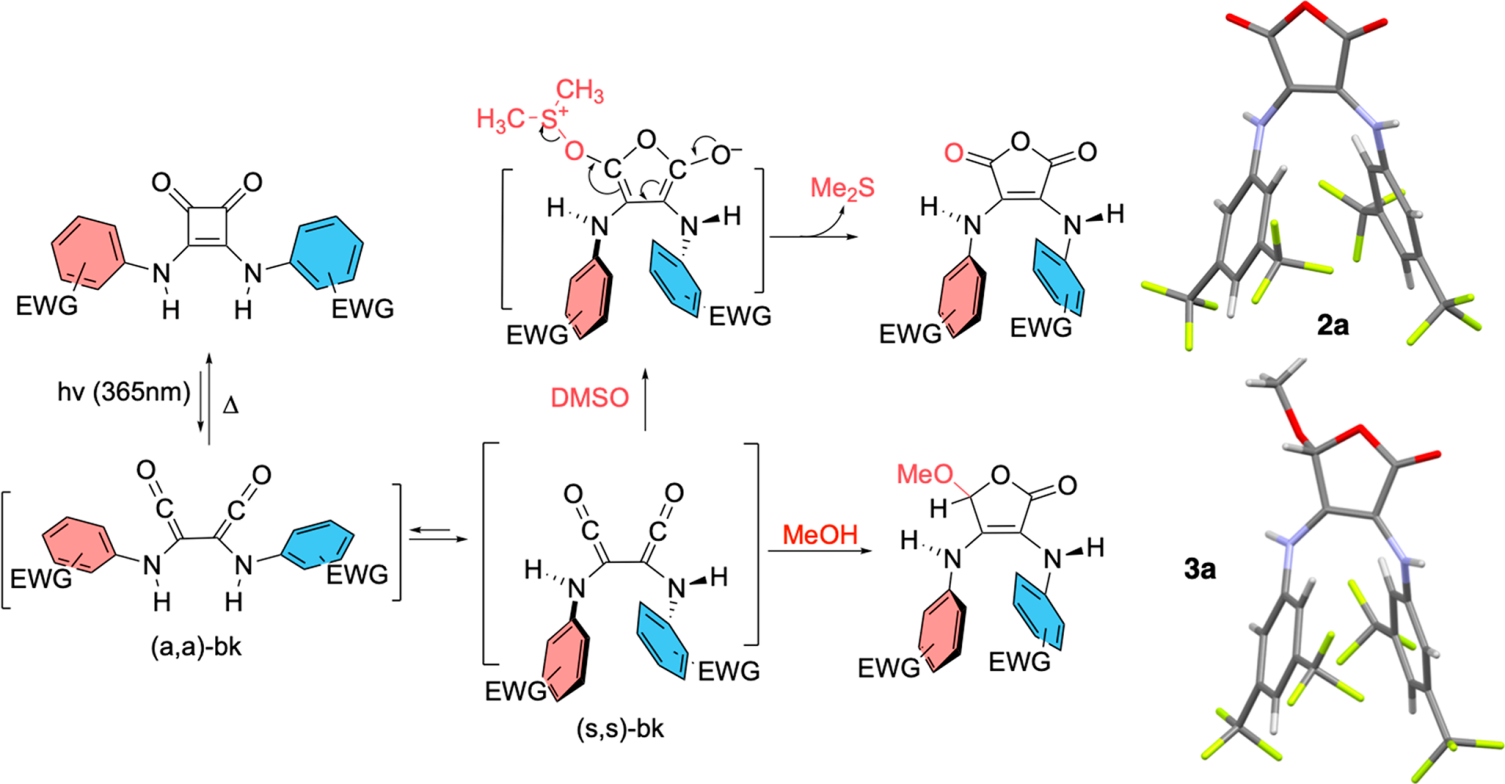
Proposed Reaction Mechanism for the Photoconversion of AD-Squaramides and X-ray Molecular Structures of Photoproducts 2a (CCDC ref. 2242682) and 3a-MeOH (CCDC ref. 2242680)

The X-ray molecular structures of **2a** and **2c** show that these anhydrides exist in a skewed (s,s)-type conformation, in agreement with NMR data. Moreover, the resulting anhydrides were soluble even in comparably low polarity solvents such as CHCl_3_, which can be attributed to the (s,s)-type orientation of the aryl rings and their poor hydrogen bonding abilities relative to the parent AD-squaramides. Density functional theory (DFT) calculations (WB97X-D/6-31G*) on both (a,a)-**2a** and (s,s)-**2a** conformers (Figure S6.1) suggest that the preference for the (s,s)-type conformers originates from the repulsive interactions of the anhydride carbonyls with its nearest aryl ring (due to the short distance existing in a “forced” planar structure) and to the attractive stacking between EWG-substituted aryl rings. Notably, a similar conformational trend has been reported in the structurally related five-membered rings, croconamides.^26^

Although the photoconversion of AD-squaramides **1** to 2,3-arylamino maleic anhydrides **2** involves oxidation, the outcome of the reactions is the same whether it is carried out in oxygen-free atmospheres or in air. This observation suggests that DMSO, used as the solvent, could also act as the oxidant and trapping agent in these reactions, which would be in agreement with the zero-order kinetics determined for **1a** (Figure S3.1).

The ability of DMSO to promote nucleophilic oxidations is well established,^28^ and this solvent has been reported to react with di-*tert*-butylketene through nucleophilic addition.^29^ In our experiments, the detection of a ^1^H NMR peak at 2.0 ppm assigned to dimethyl sulfide (Figure S4) supports the role of DMSO as the nucleophilic oxidant. This role is also consistent with the lack of photoconversion observed with electron-rich AD-squaramides **1i** and **1j**, and with the inhibited photoconversion of **1a** observed in the presence of anions basic enough to deprotonate this AD-squaramide, such as OH^–^, F^–^, or AcO^–^ (Figure S3.11).^25^ In such cases, the lower electrophilic character of the ketene carbonyls would prevent the bisketene nucleophilic trapping by the DMSO molecules. By contrast, the presence of anions which do not cause deprotonation, such as Cl^–^ or NO_3_^–^, do not significantly affect the photoconversions. This behavior is not influenced by the countercation nature (Figures S3.12-S3.15).

Taking all these results into consideration, we propose the mechanism shown in Scheme 2. After the initial and rapid light-driven ring-opening of the AD-squaramides **1**, the resulting bisketene (bk) high-energy intermediate (a,a)-bk, evolves to a relatively more stable bisketene (s,s)-bk. Then, DMSO traps the (s,s)-bk intermediate by nucleophilic addition to one of the two degenerate electrophilic centers of the bisketene, followed by the formation of the 5-membered ring. Finally, the irreversible reductive elimination of dimethyl sulfide affords the 2,3-arylamino maleic anhydrides **2**.

The observed rate differences among the different AD-squaramides can be accounted for by assuming that arene stacking favors nucleophilic trapping at the (s,s)-bisketene intermediate level. Density functional theory (DFT) calculations (WB97X-D/6-31G*) support this assumption (Figure S6.2) and it is known that attractive face-to-face aryl stacking interactions grow with the number of electron-withdrawing groups (EWG), which would explain the easy phototransformations of **1a** and **1b**.^30,31^ Moreover, the parallel aryl alignment imposed by photoproducts (s,s)-**2** could explain the poor performance observed for **1g** (4-F) relative to **1c** (4-CN), in agreement with deviations of fluorobenzene dimers from the normal dimerization trends observed in reported theoretical calculations.^32^

To study if the intermediate 1,2-bisketenes can also be trapped by other nucleophiles, we performed the phototransformation of **1a** in a nonoxidant noncomplexing medium such as MeOH. After completion at a preparative scale, and CC purification, 5-methoxy-3,4-arylamino-2(5*H*)-furanone **3a** was fully characterized by NMR spectroscopy (Figure S2.21–S2.24) and X-ray analysis (Scheme 2 and Figure S5.6). The ^1^H NMR spectrum shows two different sets of NMR peaks, thus highlighting the lack of structural symmetry of **3a**. This photoproduct corresponds to the nonoxidative trapping of the 1,2-bisketene intermediate and helps support the proposed reaction mechanism.

In aqueous media, AD-squaramide photoconversion could alter the result of technologically relevant processes such as anion transport. In line with its conformational preferences,^26^^1^H NMR titrations of **2a** with tetrabutylammonium chloride in DMSO-*d*_6_ (*K*_11_ < 5 M^–1^) and MeCN-*d*_3_ (*K*_11_ < 28 M^–1^), show very low affinity values (Figures S7.1–S7.8), in contrast to the strong chloride affinity measured for **1a** in DMSO (625 M^–1^) and MeCN (>10^4^ M^–1^). Therefore, the photoconversion of **1a**, an efficient anion transporter,^9^ should lead to irreversible inhibition of chloride transport.

We performed transport experiments on large unilamellar vesicles (LUVs) of POPC (1-palmitoyl-2-oleoyl-*sn*-glycero-3-phosphocholine) and cholesterol (7:3 ratio). The vesicles, of an average diameter of ∼160 nm (Figure S8.1), were prepared with encapsulated NaNO_3_ (225 mM) and the chloride-sensitive dye lucigenin (0.8 mM). Initially, transporter **1a** was added to a sample of vesicles as an external stock solution in methanol. Then, the sample was irradiated (365 nm, 10 W) for short periods (seconds). Addition of NaCl (25 mM) initiated the transport (Cl^–^/NO_3_^–^ antiport), which was monitored via the quenching of the fluorescence of lucigenin, a Cl^–^ sensitive dye. Under conditions of efficient transport by compound **1a**, irradiation of the LUVs at different times showed progressive activity loss, reaching almost complete deactivation after only 8 s (Figure 2). Control experiments confirmed that the activity loss is due to the photoconversion of the transporter and not due to any membrane or lucigenin degradation (Figures S8.2 and S8.3). Transport experiments performed with compounds **2a** and **3a** showed that they are much less active than **1a** (Figure S8.4). It should be noted that light-regulated anion transporters usually cannot achieve complete inhibition of the transport process.^32−38^ Our studies suggest that the efficient transport inhibition observed with **1a** relies on the irreversible nature of the photoconversion process, and the drastic change in the binding and conformational properties of the molecules upon photoconversion.

**Figure 2:**
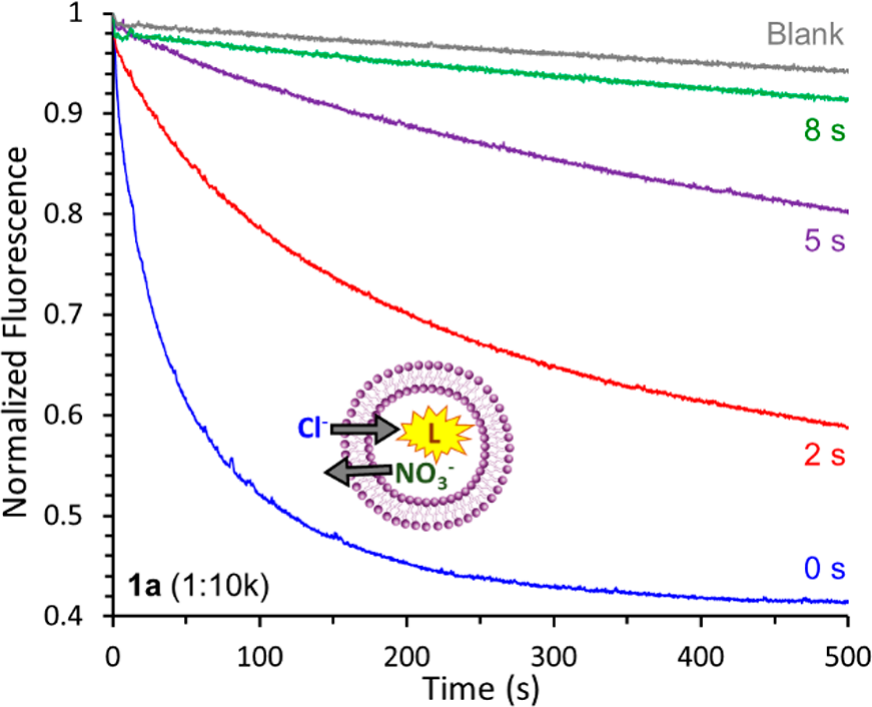
Chloride transport by squaramide **1a** (at a 1:10k transporter:lipid ratio) into LUVs after *in situ* irradiation for 0, 2, 5, and 8 s (365 nm, 10 W), studied by the lucigenin (L) assay.

To sum up, we report on a new reaction of AD-squaramides that exploits the photochemical squaramide-aminobisketene equilibrium to transform the relatively inert squaramides into 2,3-arylamino maleic anhydrides in DMSO, a process that takes place in seconds in favorable cases. In MeOH and water solutions, AD-squaramide photodegradation also occurs, likely due to the nucleophilic nature of those solvents. Our findings should be considered in future research involving irradiation of squaramide-containing compounds, for example, in photocatalysis, photoswitchable binding, and transport studies.^39^ Moreover, the photodegradation of squaramides which are used in supramolecular catalysis and anion transport offers a new way of regulating both processes. As proof of concept, we present the efficient *in situ* light-induced deactivation of a squaramide-based anion transporter. We envisage that in biomedical relevant transport processes, the phototransformation of squaramides can offer a convenient mechanism for spatiotemporal down-regulation or even termination of anion transport activity, avoiding toxicity effects due to undesired guest overtransport or cell accumulation of the transporters.

## Data Availability

The data underlying this study are available in the published article and its Supporting Information

## Supporting Information Available

General experimental procedures, NMR spectra, conversion plots, X-ray crystallographic data, ORTEP diagrams, theoretical calculations, binding model data fittings, DLS data transport measurements, UV spectra, and computational parameters and Cartesian coordinates (PDF)
