# Supplementary material for: Light-Driven Photoconversion of Squaramides with Implications in Anion Transport
Source: Org Lett. 2023 May 9;25(19):3423–8. doi: 10.1021/acs.orglett.3c00993 (PMC10204084; doi:10.1021/acs.orglett.3c00993)
Supplement: Supplementary file 1 — ol3c00993_si_001.pdf [file ol3c00993_si_001.pdf]

Supporting Information

Light-driven photoconversion of squaramides with implications in anion transport

Manel Vega, Luis Martínez-Crespo, Miguel Barceló-Oliver, Carmen Rotger* and Antonio Costa*

Department of Chemistry

Universitat de les Illes Balears, Palma, 07122, Spain.

Email: antoni.costa@uib.es; Fax: +34 971 172436; Tel: +34 971 173266.

Table of contents

**1.**	**Materials, synthesis and characterization**		3
	**1.1**	Synthesis of Aniline-Derived (AD)-squaramides	3
	**1.2**	Synthesis of 3,4-bis(arylamino)maleic anhydrides [3,4-bis(arylamino)-2,5-furane diones]	6
**2.**	**^{1}H and ^{13}C NMR spectra**		9
**3.**	**Photochemical reactions at analytical (^{1}H NMR) scale**		23
	**3.1**	General photoreaction procedure	23
	**3.2**	Photoconversion of AD-squaramides **1a-j**.	23
	**3.3**	Influence of added anions and cations on the photoconversion	33
**4.**	**Observation of dimethyl sulfide (^{1}H NMR) in a reaction sample**		36
**5.**	**Single-Crystal X-ray Diffraction**		36
	**5.1**	Crystal data and structure refinement for compounds **1b**, **1c**, **1d**, **2a**, **2c**, **3a**	37
	**5.2**	ORTEP (50%) Representations	38
**6.**	**Theoretical calculations**		40
**7.**	**^{1}H NMR titrations**		41
**8.**	**Transport experiments**		44
	**8.1**	Preparation of the vesicles	44
	**8.2**	Chloride transport experiments	44
**9**	**References**		49
	**ANNEX I.** Cartesian Coordinates		50

1. Materials, synthesis and characterization

All chemicals were obtained from commercial sources and used without further purification unless otherwise indicated. Proton nuclear magnetic resonance (^{1}H NMR) spectra and carbon nuclear resonance (^{13}C NMR) were recorded at room temperature on a Bruker Avance and Bruker Avance III spectrometers operating at 300 and 600 MHz for 1H and 75.4 and 150.9 MHz for 13-carbon, respectively. The residual proton signal in the NMR solvents were used as a reference (DMSO-d$_6$: δ 2.50 ppm, MeCN-d_3 1.94 ppm). Chemical shifts (δ) are reported in ppm and coupling constants (J) in Hz. Multiplicities for proton signals are abbreviated as s, t, m, and br for singlet, triplet, multiplet, and broad signal, respectively. Structural assignments were made with additional information from gHSQC and gHMBC experiments. High-resolution mass spectra (HRMS) were recorded on a Thermo Scientific Orbitrap Q-Exactive mass spectrometer equipped with a heated electrospray module (HESI-HRMS). FT-IR spectra were obtained FT-IR Tensor 27 with Platinum-ATR accessory. Spectral features are tabulated as follows: wavenumber (cm^{-1}); intensity: strong (s), medium (m), and weak (w).

1.1 Synthesis of Aniline-Derived (AD)-squaramides

General synthetic procedure for preparation of AD-squaramides

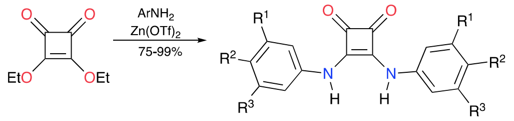

A suspension of diethyl squarate (340 mg, 2 mmol, 1 equiv.), zinc triflate (145 mg, 0.4 mmol, 0.2 equiv.) and the corresponding substituted aniline (4.4 mmol, 2.2 equiv.) in *n*-octanol (6 mL) was heated in an oil bath at 100 °C with stirring for 15 h. Upon cooling, the crude mixture was diluted with EtOAc (20 mL) and the solid filtered through a glass filter with sintered glass disc (porosity 4).

3,4-bis((3,5-bis(trifluoromethyl)phenyl)amino)cyclobut-3-ene-1,2-dione (1a).[s1]

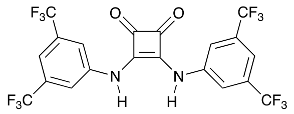

The title compound was obtained following the general procedure by mixing diethyl squarate (340 mg, 2 mmol, 1 equiv.), zinc triflate (145 mg, 0.4 mmol, 0.2 equiv.) and 3,5-bis(trifluoromethyl)aniline (1 g, 4.4 mmol, 2.2 equiv.) in *n*-octanol (6 mL). After heating and initial filtration, the solid was washed with EtOAc (5 × 10 mL), air-dried, and washed again with water (4 × 10 mL). The resulting solid was air-dried and then oven-dried at 105 °C overnight to afford **1a** as an amorphous white solid, 981 mg (91 % yield).

3,4-bis((3,4-biscyanophenyl)amino)cyclobut-3-ene-1,2-dione (1b).

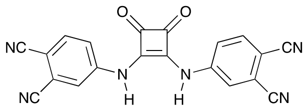

In this case the general synthetic procedure using n-octanol leads to a mixture of squaramide **1b** (24%) and the competitive squaraine. Therefore, the title compound was obtained following the original procedure, mixing a diethyl squarate (340 mg, 2 mmol, 1 equiv.), zinc triflate (145 mg, 0.4 mmol, 0.2 equiv.) and 5-aminoisophthalonitrile (630 mg, 4.4 mmol, 2.2 equiv.) in a toluene-DMF (19:1) solvent mixture.[s1] After heating in an oil bath and initial filtration, the solid was washed with EtOH (5 × 10 mL), suspended in hot DMSO (20 mL) and water (5 mL) and maintained at 70 °C in an oil bath under stirring for 24 h. After addition of water (30 mL) the solid was filtered, washed with water (10 × 10 mL). The solid was air-dried and then oven-dried at 105 °C overnight to afford **1b** as an amorphous yellow solid, 670 mg (92 % yield), Crystals suitable for X-ray analysis were obtained by slow crystallization in CD$_3$CN. mp > 300 °C dec. ^{1}H NMR (300 MHz, DMSO-d$_6$): δ_H 10.71 (s, 2H, NH), 8.12 (d, J = 8.7 Hz, 2H, -Ar), 8.05 (d, J = 2.1 Hz, 2H, -Ar), 7.78

(dd, J = 8.7 Hz, J = 2.1 Hz, 2H, -Ar) ppm. ^{13}C NMR (75 MHz, DMSO-d_6): δ_C 183.3, 166.0, 142.7, 135.4, 123.2, 123.0, 116.1, 115.8, 115.7, 107.6 ppm. IR (powder; cm^{-1}): 3261, 3168, 3047, 2228, 1805, 1695, 1590, 1537, 1440, 1403, 1315, 1253, 854, 734, 523. HRMS (ESI) m/z: $[M-H]^-$ Calcd for $C_{20}H_7N_6O_2$: 363.0630; Found 363.0633.

3,4-bis((4-cyanophenyl)amino)cyclobut-3-ene-1,2-dione (1c).

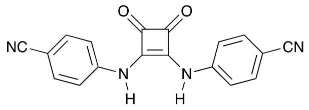

The title compound was obtained following the general synthetic procedure mixing diethyl squarate (340 mg, 2 mmol, 1 equiv.), zinc triflate (145 mg, 0.4 mmol, 0.2 equiv.) and 4-aminobenzonitrile (520 mg, 4.4 mmol, 2.2 equiv.) in n-octanol (6 mL). After heating in an oil bath and initial filtration, the solid was washed with EtOAc (5 × 10 mL) and MeOH (5 × 10 mL). Then, the solid was digested with hot DMSO (20 mL) and water (5 mL) and maintained at 70 °C in an oil bath under stirring for 24 h. After addition of water (30 mL) the solid was filtered and washed with water (10 × 10 mL). Finally, the solid was air-dried and then oven-dried at 105 °C overnight to afford **1c** as an amorphous yellow solid, 597 mg (95 % yield), mp > 300 °C dec. ^1H NMR (300 MHz, DMSO-d_6): δ_H 10.31 (s, 2H, NH), 7.84 (d, J = 7.5 Hz, 4H, -Ar), 7.61 (d, J = 7.8 Hz, 4H, -Ar) ppm. ^{13}C NMR (75 MHz, DMSO-d_6): δ_C 182.5, 166.0, 142.4, 133.8, 119.0, 118.9, 105.0 ppm. IR (powder; cm^{-1}): 3286, 2222, 1793, 1681, 1594, 1515, 1386, 1225, 1178, 833, 656, 540. HRMS (ESI) m/z: $[M-H]^-$ Calcd for $C_{18}H_9N_4O_2$ 313.0731; Found 313.0727.

3,4-bis((4-aminosulphonylphenyl)amino)cyclobut-3-ene-1,2-dione (1d).

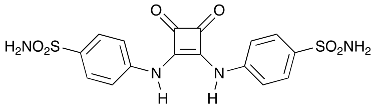

The title compound was obtained following the general synthetic procedure mixing diethyl squarate (340 mg, 2 mmol, 1 equiv.), zinc triflate (145 mg, 0.4 mmol, 0.2 equiv.) and 4-aminobenzenesulfonamide (758 mg, 4.4 mmol, 2.2 equiv.) in n-octanol (6 mL). After heating in an oil bath and initial filtration, the solid was washed with EtOAc (5 × 10 mL) and digested in hot DMSO (10 mL) and water (5 mL) at 70°C in an oil bath for 24h. After addition of water (30 mL) the mixture was centrifuged at 5000 rpm for 15 min. Then the solid was suspended in water and centrifuged again (10 × 10 mL). Finally, the solid was air-dried and then oven-dried at 105 °C overnight to afford **1d** as an amorphous yellow solid, 658 mg (78 % yield). mp > 300 °C dec. ^1H NMR (300 MHz, DMSO-d_6): δ_H 10.21 (s, 2H, -NH-), 7.81 (d, J = 8.4 Hz, 4H, -Ar), 7.6 (d, J = 8.4 Hz, 4H, -Ar), 7.32 (s, 4H, -SO2NH2) ppm. ^{13}C NMR (75 MHz, DMSO-d_6): δ_C 182.2, 166.0, 141.2, 138.5, 127.3, 118.5 ppm. IR (powder; cm^{-1}): 3404, 3243, 3152, 3080, 2998, 1799, 1673, 1607, 1595, 1534, 1437, 1330, 1153, 1099, 854, 743, 688, 540. HRMS (ESI) m/z: $[M-H]^-$Calcd for $C_{16}H_{13}N_4O_6S_2$ 421.0282; Found 421.0285.

3,4-bis((4-ethoxycarbonylphenyl)amino)cyclobut-3-ene-1,2-dione (1e).

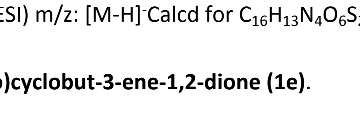

In this case the general synthetic procedure using *n*-octanol affords the n-octylester instead of **1e** (ethyl ester) due to simultaneous and unavoidable transesterification. Therefore, the title compound was obtained following the original procedure mixing diethyl squarate (340 mg, 2 mmol, 1 equiv.), zinc triflate (145 mg, 0.4 mmol, 0.2 equiv.) and ethyl 4-aminobenzoate (727 mg, 4.4 mmol, 2.2 equiv.) in a toluene-DMF (19:1) solvent mixture.[s1] After heating in an oil bath and initial filtration, the solid was washed with MeOH (5 × 10 mL) and water (5 × 10 mL). Then, the solid was air-dried and then oven-dried at 105 °C overnight to afford **1e** as an amorphous yellow solid, 767 mg (91 % yield), mp > 300 °C dec. ^1H NMR (300 MHz, DMSO-d_6): δ_H 10.23 (s, 2H, -NH-), 7.97 (d, J = 8.7 Hz, 4H, -Ar), 7.59 (d, J = 8.4 Hz, 4H, -Ar), 4.3 (q, J = 6.9, 4H, -CH_2-), 1.32 (t, J = 6.9, 6H, -CH_3) ppm. ^{13}C NMR (75 MHz, DMSO-d_6): δ_C 182.2, 166.0, 165.2, 142.7, 130.8, 124.2, 118.1 ppm. IR (powder; cm^{-1}):3143, 3067, 2980, 1797, 1713, 1677, 1599, 1542, 1449, 1266, 851, 765, 691, 502. HRMS (ESI) m/z: $[M-H]^-$ Calcd for $C_{22}H_{19}N_2O_6$ 407.1249; Found 407.1250.

3,4-bis((4-nitrocarbonylphenyl)amino)cyclobut-3-ene-1,2-dione (1f).[s1]

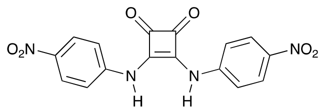

The title compound was obtained following the general procedure. After heating in an oil bath and initial filtration, the solid was washed with MeOH (5 × 10 mL) and digested in hot DMSO (20 mL) and water (5 mL) at 70 °C in an oil bath under stirring for 24 h. After addition of water (30 mL) the solid was filtered and washed with water (10 × 10 mL). Finally, the solid was air-dried and then oven-dried at 105 °C overnight to afford **1f** as an amorphous yellow solid, 673 mg (95 % yield).

3,4-bis((4-fluorophenyl)amino)cyclobut-3-ene-1,2-dione (1g).[s2]

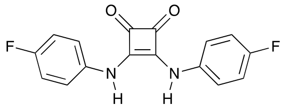

The title compound was obtained following the general procedure. After heating in an oil bath and initial filtration, the solid was washed sequentially with EtOAc (5 × 10 mL), MeOH (5 × 10 mL) and water (5 × 10 mL), air dried and then oven-dried at 105 °C overnight to afford **1g** as an amorphous pale green solid, 550 mg (92 % yield).

3,4-bis(phenylamino)cyclobut-3-ene-1,2-dione (1h).[s1]

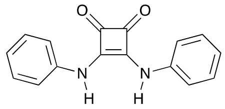

The title compound was obtained following the general procedure. After heating in an oil bath and initial filtration, the solid was washed with EtOH (5 × 10 mL) and water (5 × 10 mL), air dried and then oven-dried at 105 °C overnight to afford **1h** as an amorphous pale-yellow solid, 506 mg (96 % yield).

3,4-bis((4-methoxyphenyl)amino)cyclobut-3-ene-1,2-dione (1i).[s1]

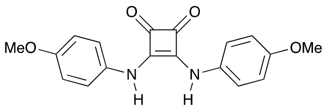

The title compound was obtained following the general procedure. After heating in an oil bath and initial filtration, the solid was washed with EtOAc (5 × 10 mL) and water (5 × 15 mL), air dried and then oven-dried at 105 °C overnight at vacuum on P_2O_5, to afford **1i** as an amorphous pale-yellow solid, 647 mg (99 % yield).

3,4-bis((4-hydroxyphenyl)amino)cyclobut-3-ene-1,2-dione (1j).

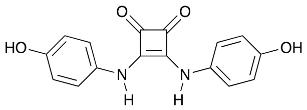

The title compound was obtained following the general procedure mixing diethyl squarate (340 mg, 2 mmol, 1 equiv.), zinc triflate (145 mg, 0.4 mmol, 0.2 equiv.) and 4-aminophenol (480 mg, 4.4 mmol, 2.2 equiv.) in n-octanol (6 mL). After heating in an oil bath and initial filtration, the solid was washed with EtOAc (5 × 10 mL) and water (5 × 15 mL), air dried and then oven-dried at 105 °C overnight, to afford **1j** as an amorphous ochre solid, 552 mg (93 % yield). mp

> 300 °C dec. ^{1}H NMR (300 MHz, DMSO-d_6): δ_H 9.57 (s, 2H, -NH-), 9.33 (s, 2H, -OH) 7.28 (d, J = 9 Hz, 4H, -Ar), 6.76 (d, J = 8.7 Hz, 4H, -Ar) ppm. ^{13}C NMR (75 MHz, DMSO-d_6): δ_C 181.0, 164.9, 153.7, 130.4, 120.1, 115.7 ppm. IR (powder; cm^{-1}): 3254, 1791, 1650, 1570, 1539, 1511, 1454, 1242, 826, 500.. HRMS (ESI) m/z: [M − H]$^-$ Calcd for $C_{16}H_{11}N_2O_4$: 295.0724; Found 295.0726.

1.2 Synthesis of 3,4-bis(arylamino)maleic anhydrides [3,4-bis(arylamino)-2,5-furane diones]

General synthetic procedure for preparation of 3,4-bis(arylamino)-2,5-furane diones.

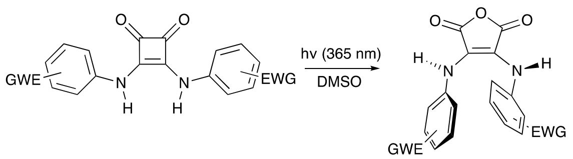

The AD-squaramide (0.5 mmol, 1 equiv.) was dissolved in a dry THF-DMSO (2:1) solvent mixture in an Schlenk tube. The air-refrigerated tube (temp. ca 28°C) was placed 2 cm away related to the external wall of a conventional borosilicate glass photoreactor and irradiated with a 400 W UV medium-pressure lamp. The photoconversion was followed by ^{1}H NMR analysis taking aliquots at certain time intervals. After completion (5-20 h), the reaction mixture was diluted with EtOAc (50 mL) and phase-partitioned with brine (5 × 15 mL). The organic layer was dried on anhydrous Na_2SO_4, percolated over a short (10 cm length, 2 cm O.D.) SiO_2 column and the solvent eliminated at reduced pressure.

3,4-bis((3,5-bis(trifluoromethyl)phenyl)amino)furan-2,5-dione (2a).

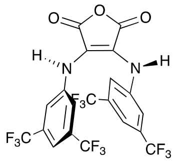

AD-squaramide **1a** (536 mg, 1.0 mmol, 1. equiv.) dissolved in dry DMSO (20 mL) and THF (10 mL) was loaded in an Schlenk tube located 1.5-2 cm cm away to the external wall of the glass photoreactor. Then the solution was irradiated for 10 h (temperature < 28 ºC). After completion the reaction mixture was diluted with EtOAc (50 mL) and phase-partitioned with brine (5 × 15 mL). The organic layer was dried on anhydrous Na_2SO_4, percolated over a short (10 cm length, 2 cm O.D.) SiO_2 column and the solvent eliminated at reduced pressure. The residue was purified by column chromatography (SiO_2; *iso*-propyl ether/hexane (1:1 v/v)), to afford **2a** as a yellow solid (440 mg, 79% yield). mp 191-193 °C. Crystals suitable for X-ray analysis were grown by slow crystallization in MeCN. ^{1}H NMR (300 MHz, DMSO-d_6): δ_H 9.91 (s, 2H, -NH-), 7.16 (s, 2H, -Ar), 7.11 (s, 4H, -Ar) ppm. ^{13}C NMR (75 MHz, DMSO-d_6): δ_C 163.4, 141.2, 129.9 (q, J_{C-F} = 32.8 Hz), 124.8, 121.2, 118.1, 113.2 ppm. IR (powder; cm^{-1}): 3407, 3363, 3101, 1845, 1749, 1681, 1621, 1528, 1380, 1272, 1168, 1124, 1098, 876, 847, 702, 682, 591. HRMS (ESI) m/z: [M-H]$^-$ Calcd for $C_{20}H_7F_{12}N_2O_3$ 551.0271; Found. 551.0271.

3,4-bis(3,4-bis(cyanophenyl)amino)furan-2,5-dione (2b).

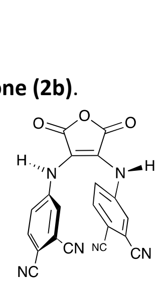

The title compound was obtained according to the general synthetic procedure from **1b** (182.5 mg, 0.5 mmol, 1 equiv.) dissolved in DMSO (10 mL) and THF (5 mL). Total irradiation time 5h. The crude product was purified by column chromatography (SiO_2; EtOAc) to afford **2b** as an orange solid (130 mg, 68 % yield). mp > 250 °C dec. ^{1}H NMR (300 MHz, DMSO-d_6): δ_H 10.23 (s, 2H, -NH-), 7.76 (d, J = 8.6 Hz, 2H, -Ar), 7.16 (d, J = 2.3 Hz, 2H, -Ar), 7.07 (dd, J = 8.7 Hz, J = 2.3 Hz, 2H, -Ar) ppm. ^{13}C NMR (75 MHz, DMSO-d_6): δ_C 162.8, 144.1, 134.1, 121.9, 121.8, 119.6, 116.1, 115.5, 114.7,

104.8 ppm. IR (powder; cm^{-1}): 3253, 3082, 2236, 1850, 1760, 1678, 1600, 1520, 1409, 1342, 1259, 1114, 839, 744, 522. HRMS (ESI) m/z: $[M-H]^-$ Calcd for $C_{20}H_7N_6O_3$ 379.0585; found. 379.0585.

4-bis(4-cyanophenyl)amino)furan-2,5-dione (2c).

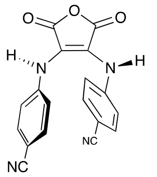

The title compound was obtained according to the general synthetic procedure from **1c** (187 mg, 0.5 mmol, 1 equiv.) dissolved in DMSO (10 mL) and THF (10 mL). Total irradiation time 6 h. The crude product was purified by column chromatography (SiO_2; EtOAc) to afford **2c** as an orange solid (150 mg, 90 % yield). Crystals suitable for X-ray analysis were obtained by slow crystallization in MeCN. mp > 200 °C dec. ^{1}H NMR (300 MHz, DMSO-d_6): δ_H 9.68 (s, 2H, -NH-), 7.41 (d, J = 8.7 Hz, 4H, -Ar), 6.76 (d, J = 8.8 Hz, 4H, -Ar) ppm. ^{13}C NMR (75 MHz, DMSO-d_6): δ_C 163.3, 144.3, 132.4, 119.4, 119.1, 118.0, 102.1 ppm. IR (powder; cm^{-1}): 3321, 2923, 2223, 2212, 1824, 1736, 1665, 1603, 1509, 1416, 1340, 1270, 1173, 929, 823, 747, 590, 543, 413. HRMS (ESI) m/z: $[M-H]^-$ Calcd for $C_{18}H_9N_4O_3$ 329.06801; Found 329.0678.

4-bis(4-aminosulfonylphenyl)amino)furan-2,5-dione (2d).

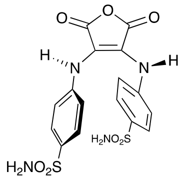

The title compound was obtained according to the general synthetic procedure from **1d** (211.2 mg, 0.5 mmol, 1 equiv.) dissolved in DMSO (10 mL). Total irradiation time 11 h. The crude product was purified by column chromatography (SiO_2; EtOAc) to afford **2d** as a yellow solid (120 mg, 55 % yield). mp 200 °C dec. ^{1}H NMR (300 MHz, DMSO-d_6): δ_H 9.34 (s, 2H, -NH-), 7.45 (d, *J* = 8.7 Hz, 4H, -Ar), 7.07 (s, 4H, SO2NH2), 6.82 (d, *J* = 8.7 Hz, 4H, -Ar) ppm. ^{13}C NMR (75 MHz, DMSO-d_6): δ_C 163.2, 143.1, 135.9, 126.1, 119.9, 117.4 ppm. IR (powder; cm^{-1}): 3265, 1828, 1747, 1668, 1593, 1515, 1411, 1274, 1148, 1096, 905, 825, 537. HRMS (ESI) m/z: $[M-H]^-$ Calcd for $C_{16}H_{13}N_4O_7S_2$ 437.0231; Found 437.0231.

4-bis(4-ethoxycarbonylphenyl)amino)furan-2,5-dione (2e).

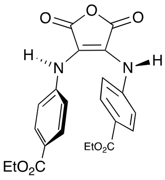

The title compound was obtained according to the general synthetic procedure from **1e** (204.2 mg, 0.5 mmol, 1 equiv.) dissolved in DMSO (10 mL) and THF (10 mL). Total irradiation time 15 h. The crude product was purified by column chromatography (SiO_2; EtOAc-hexane, 1:1 v/v) to afford **2e** as a pale orange solid (156 mg, 74 % yield). mp 164 - 166 °C. ^{1}H NMR (300 MHz, DMSO-d_6): δ_H 9.52 (s, 2H, -NH-), 7.55 (d, *J* = 8.7 Hz, 4H, -Ar), 6.76 (d, *J* = 8.7 Hz, 4H, -Ar), 4.19 (q, J = 7.1, 4H, -CH2-), 1.25 (t, *J* = 7.1, 6H, -CH3) ppm. ^{13}C NMR (75 MHz, DMSO-d_6): δ_C 182.2, 166.0, 165.2, 142.7, 130.8, 124.2, 118.1, 60.5, 14.2 ppm. IR (powder; cm^{-1}): 3331, 2927, 1828, 1732, 1698, 1604, 1523, 1417, 1273, 1256, 1177, 1101, 944, 841, 766, 744, 528, 487. HRMS (ESI) m/z: $[M-H]^-$ Calcd for $C_{22}H_{19}N_2O_7$ 423.1198; Found 423.1198.

3,4-bis((3,5-bis(trifluoromethyl)phenyl)amino)-5-methoxyfuran-2(5H)-one (3a).

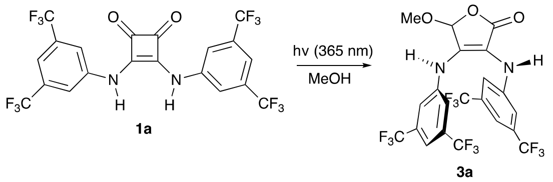

AD-squaramide **1a** (268 mg, 0.5mmol, 1 equiv.) in MeOH (50 mL) were irradiated as described above until completion. Total irradiation time 16 h. After solvent removal, the crude was purified by column chromatography (SiO_2; hexane-dioxane, 4:1 v/v) to afford **3a** as a white solid (95 mg, 33 % yield). mp 78 - 80 °C. Crystals suitable for X-ray analysis were grown in MeCN. NMR (600 MHz, CD_3CN): δ_H 7.97 (s, 1H, -NH-), 7.32 (s, 1H, Ar), 7.2 (s, 2H, Ar), 7.06 (s, 1H, Ar), 6.92 (s, 1H, -NH-), 6.81 (s, 2H, Ar), 5.94 (s, 1H, (MeO)CH(-O-)), 3.62 (s, 3H, -OCH3) ppm. ^{13}C NMR (150 MHz, CD_3CN): 169.5, 145.1, 140.6, 139.7, 132.2 (q, J_{C-F} = 33 Hz), 131.9 (q, J_{C-F} = 33 Hz), 124.4 (q, J_{C-F} = 271 Hz), 124.2 (q, J_{C-F} = 271 Hz), 120.9, 116.8 (t, J_{C-F} = 4 Hz), 116.0, 112.7 (t, J_{C-F} = 4 Hz), 105.7, 100.3, 56.6 δ_C ppm. IR (powder; cm^{-1}): 3296, 3090, 1749, 1667, 1622, 1536, 1471, 1381, 1275, 1169, 1122, 1092, 977, 939, 873, 702, 682. HRMS (ESI) m/z: $[M-H]^-$ Calcd for $C_{21}H_{11}F_{12}N_2O_3^-$ 567.0584; Found 567.0591.

2. ^1H and ^{13}C NMR spectra

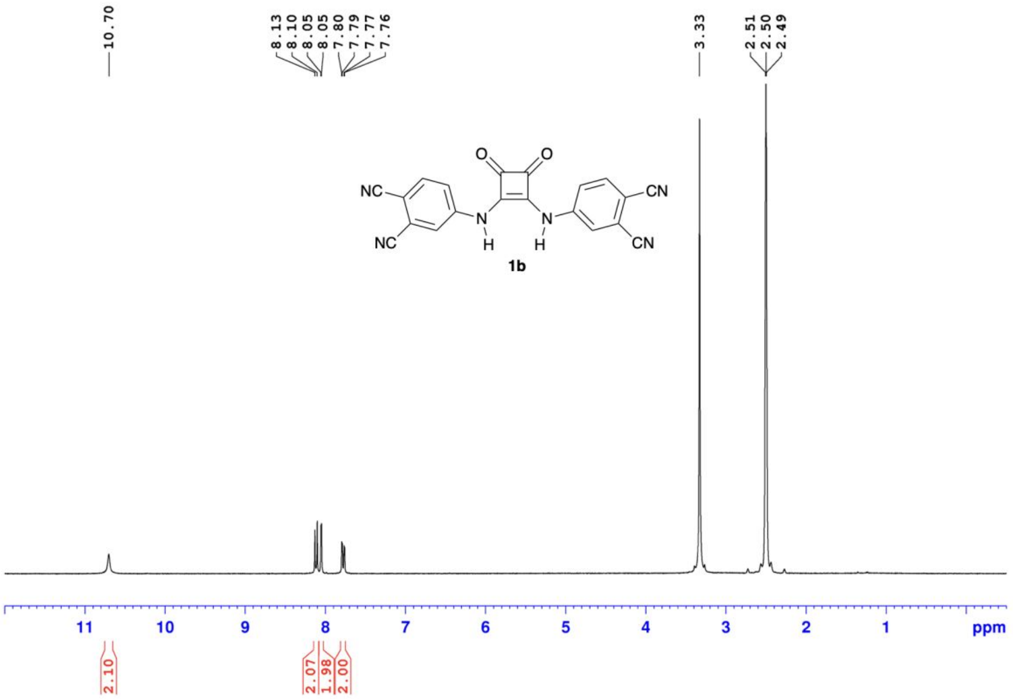

Figure S2.1. ^1H NMR (300 MHz) spectrum of AD-squaramide **1b** in DMSO-d_6

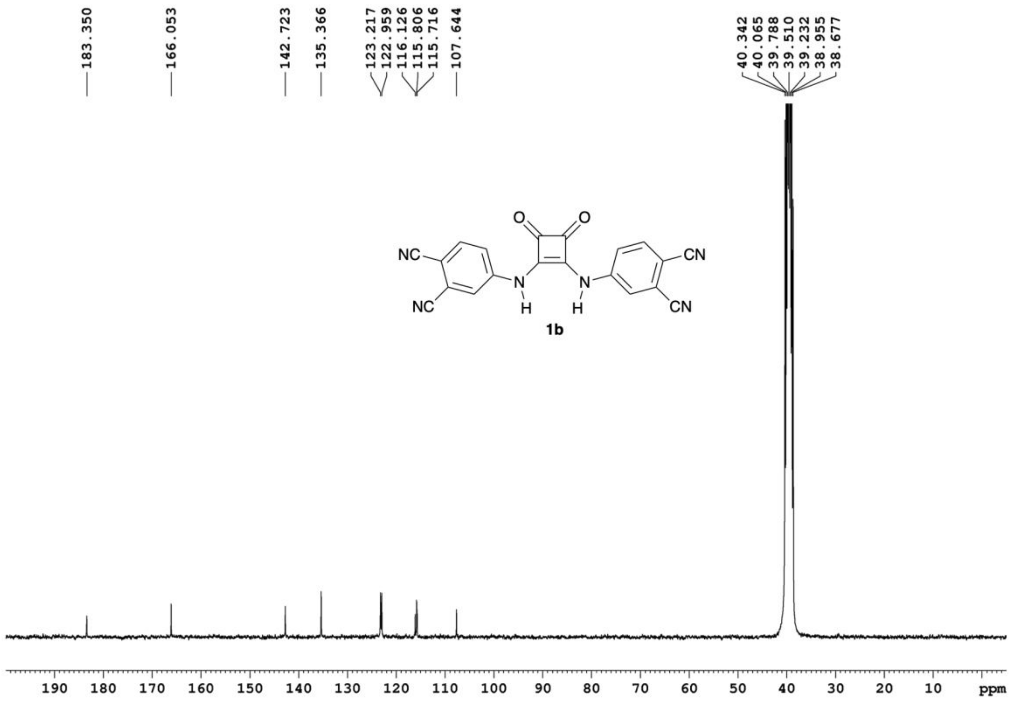

Figure S2.2. ^{13}C NMR (75 MHz) spectrum of AD-squaramide **1b** in DMSO-d_6

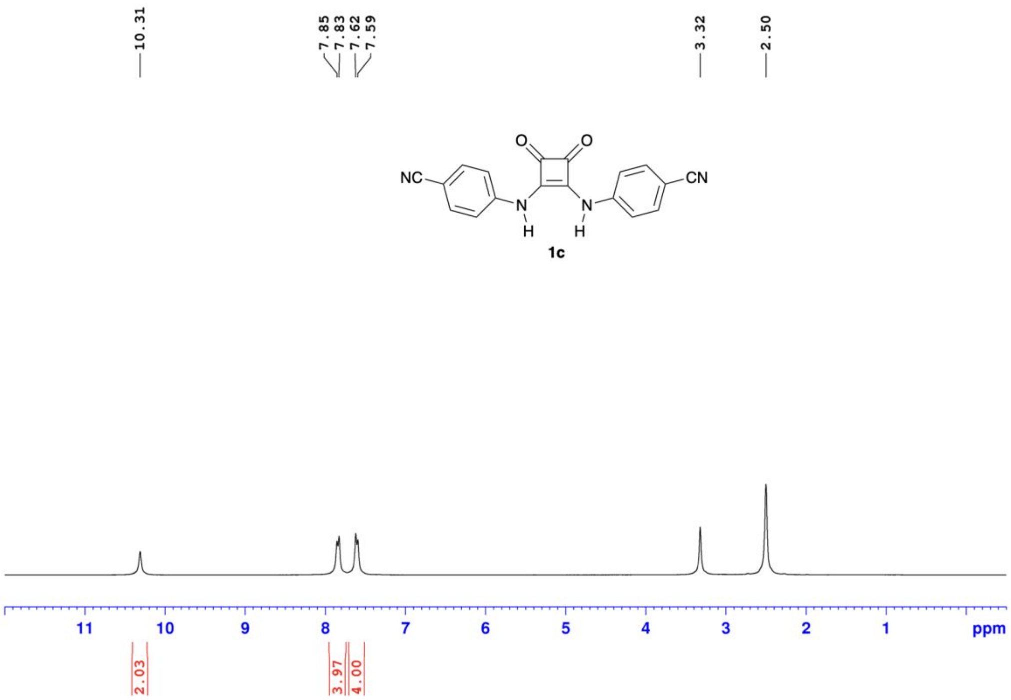

Figure S2.3. ^{1}H NMR (300 MHz) spectrum of AD-squaramide **1c** in DMSO-d_6

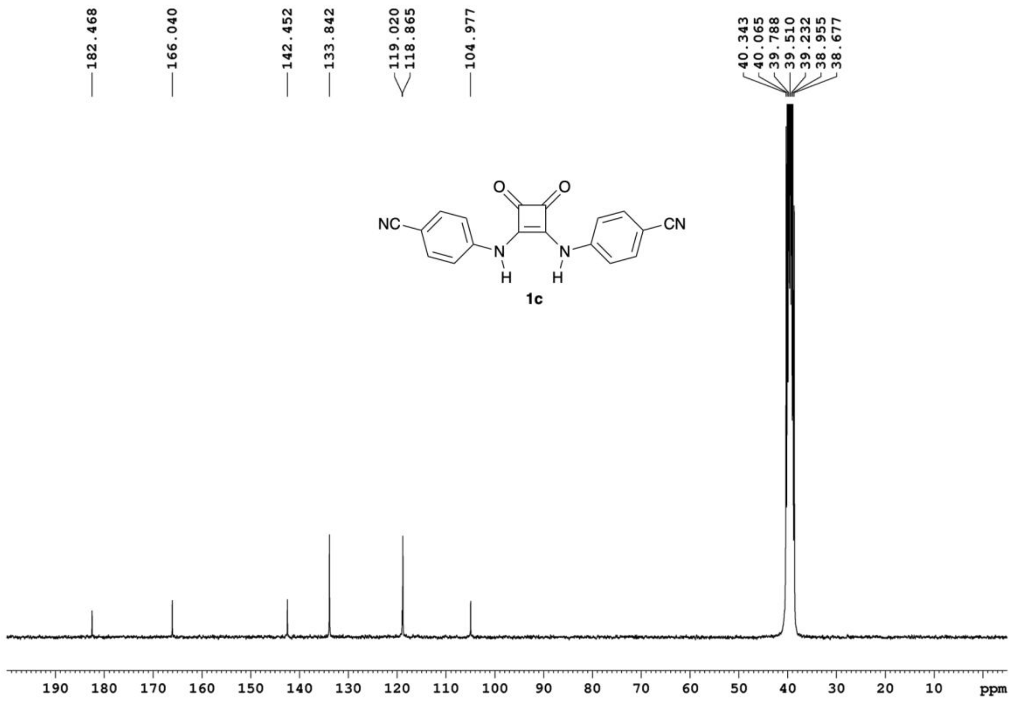

Figure S2.4. ^{13}C NMR (75 MHz) spectrum of AD-squaramide **1c** in DMSO-d_6

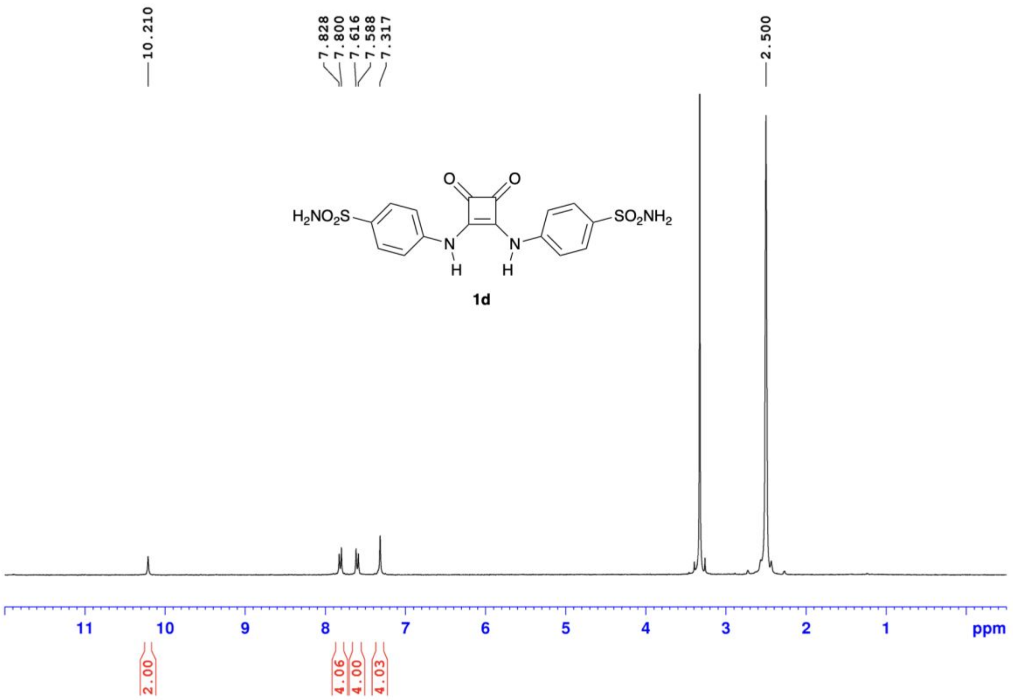

Figure S2.5. ^{1}H NMR (300 MHz) spectrum of AD-squaramide **1d** in DMSO-d_6

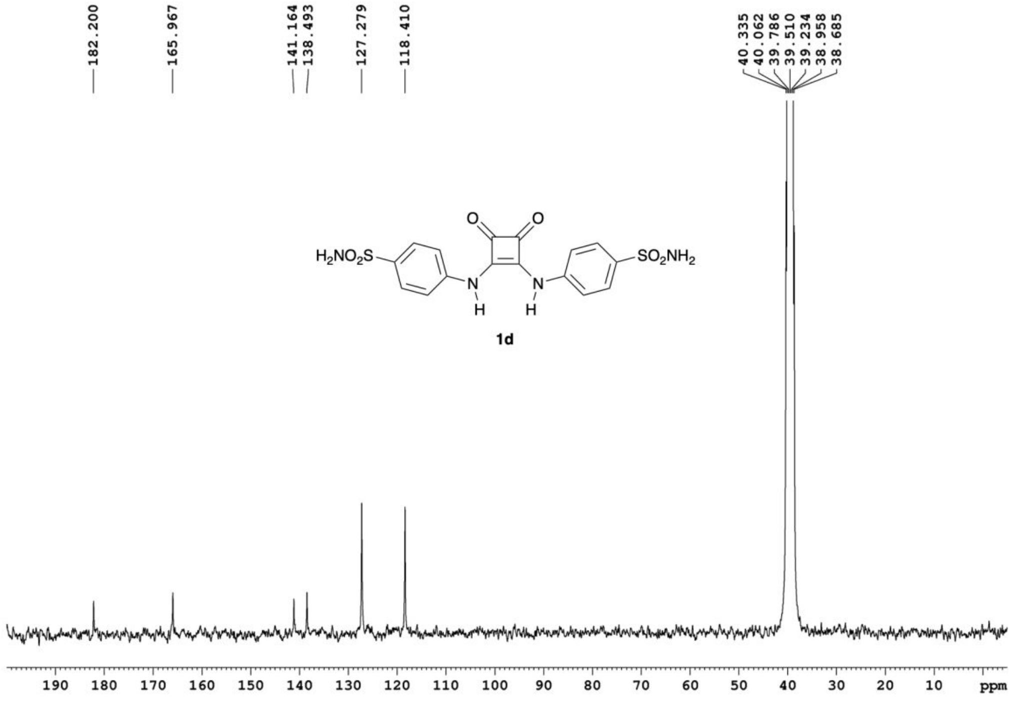

Figure S2.6. ^{13}C NMR (75 MHz) spectrum of AD-squaramide **1d** in DMSO-d_6

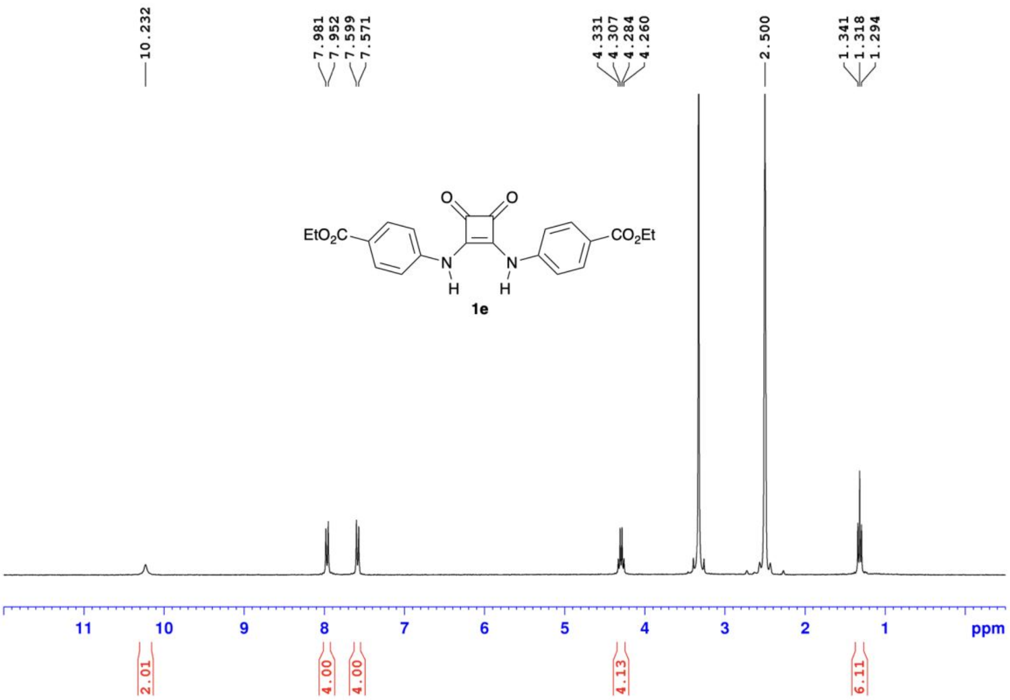

Figure S2.7. ^{1}H NMR (300 MHz) spectrum of AD-squaramide **1e** in DMSO-d_6

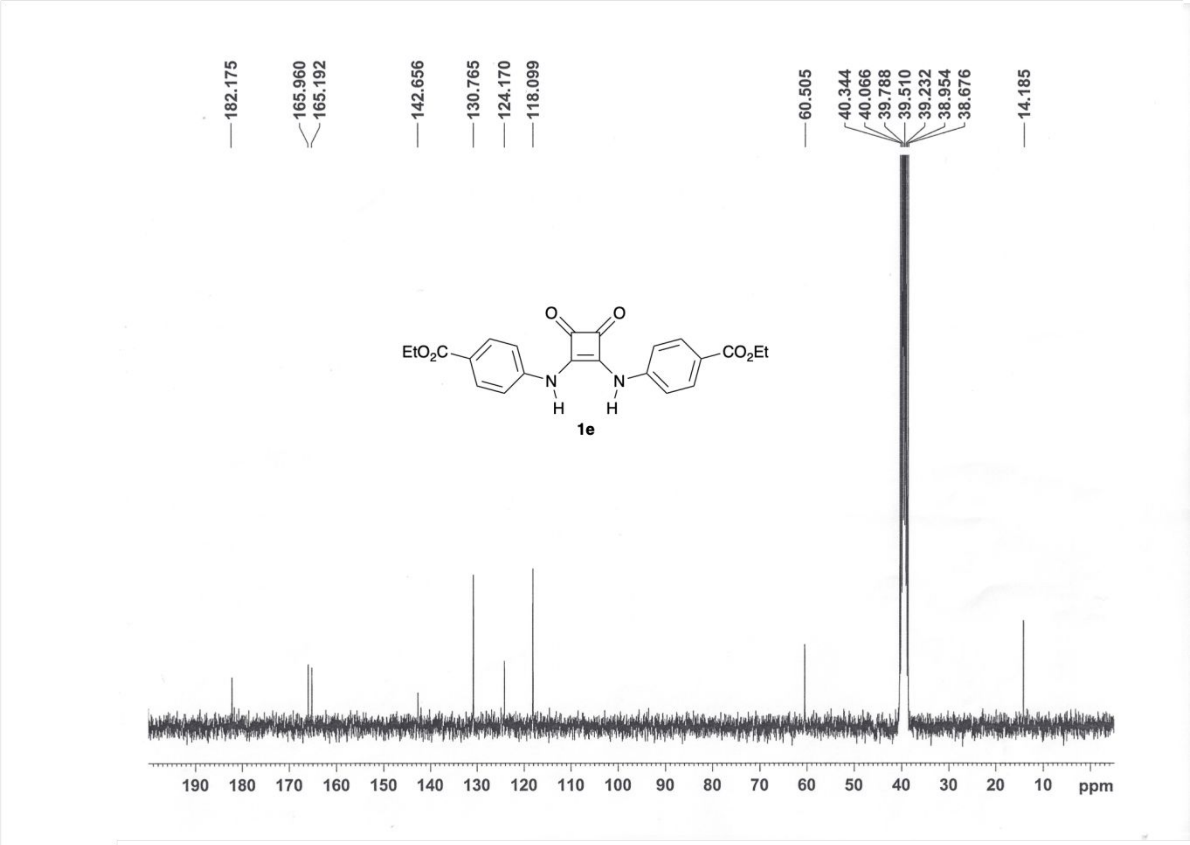

Figure S2.8. ^{13}C NMR (75 MHz) spectrum of AD-squaramide **1e** in DMSO-d_6

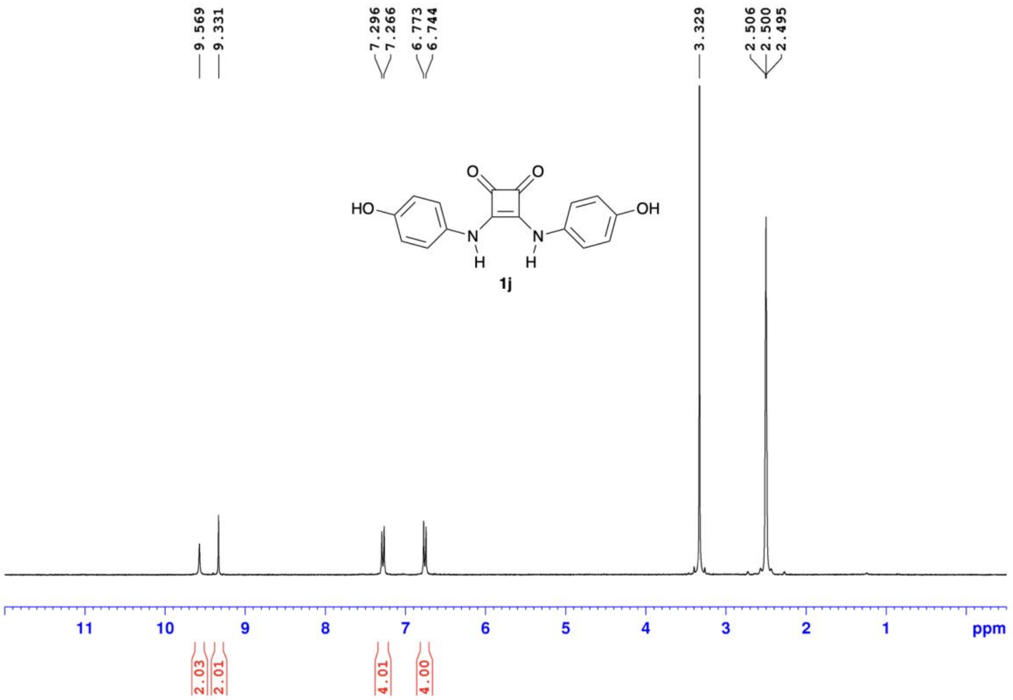

Figure S2.9. ^{1}H NMR (300 MHz) spectrum of AD-squaramide **1j** in DMSO-d_6

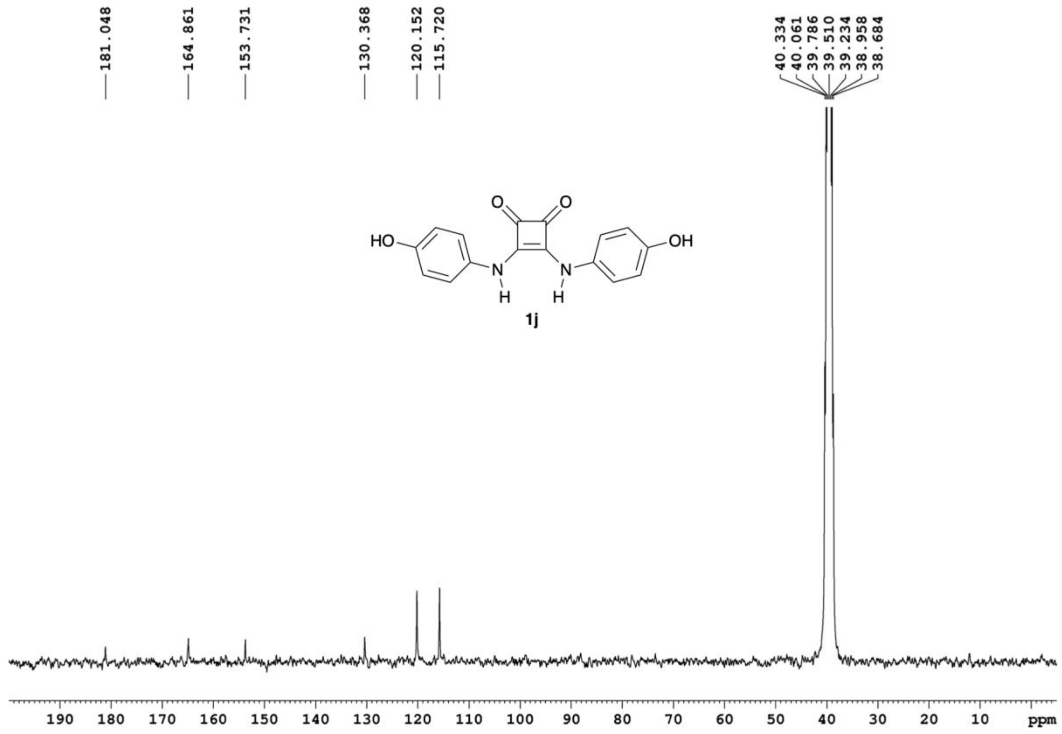

Figure S2.10. ^{13}C NMR (75 MHz) spectrum of AD-squaramide **1j** in DMSO-d_6

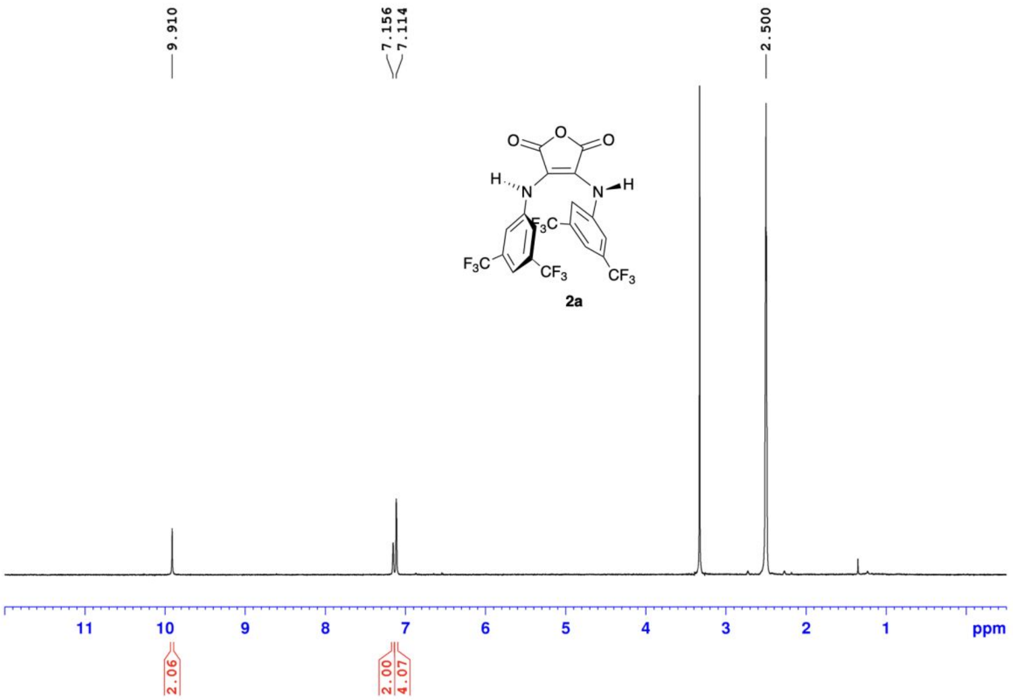

Figure S2.11. ^{1}H NMR (300 MHz) spectrum of anhydride **2a** in DMSO-d_6

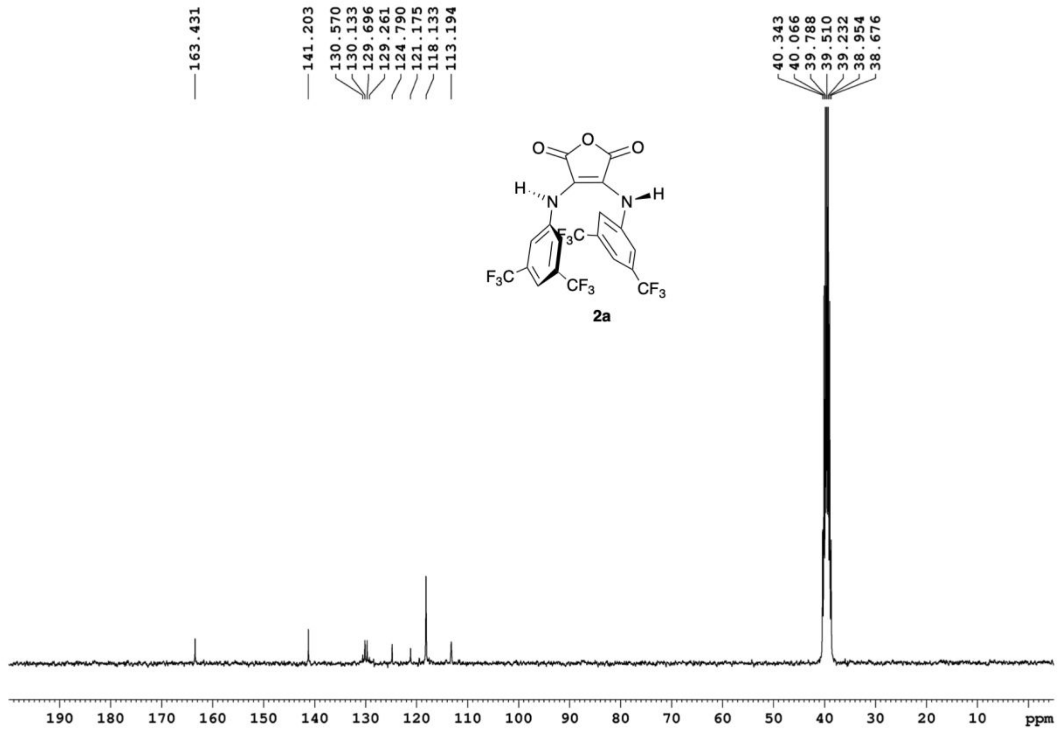

Figure S2.12. ^{13}C NMR (75 MHz) spectrum of anhydride **2a** in DMSO-d_6

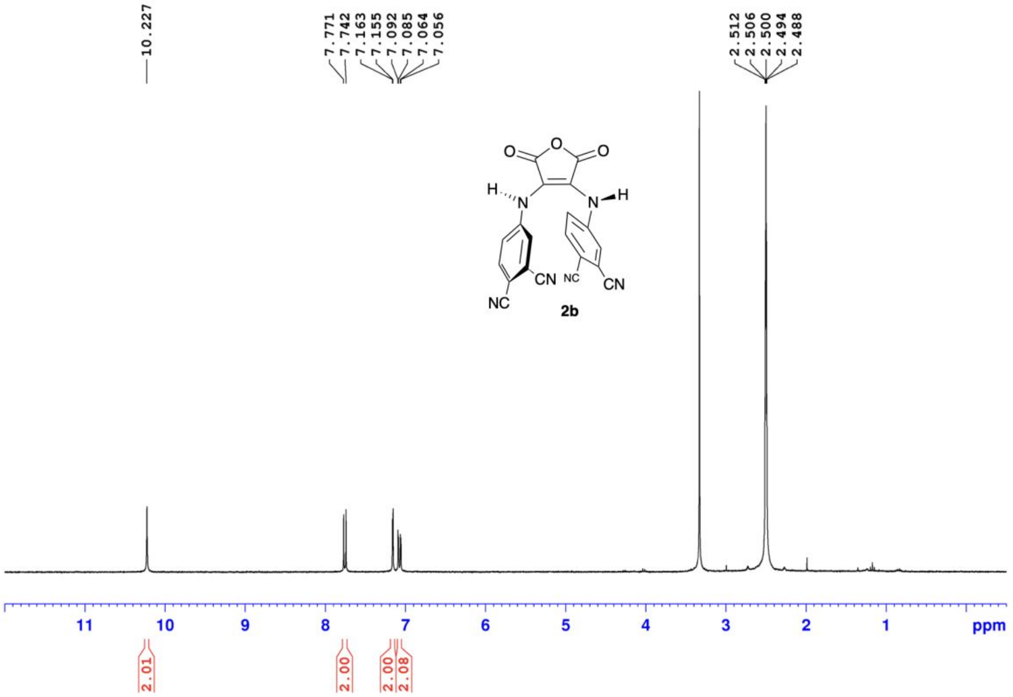

Figure S2.13. ^{1}H NMR (300 MHz) spectrum of anhydride **2b** in DMSO-d_6

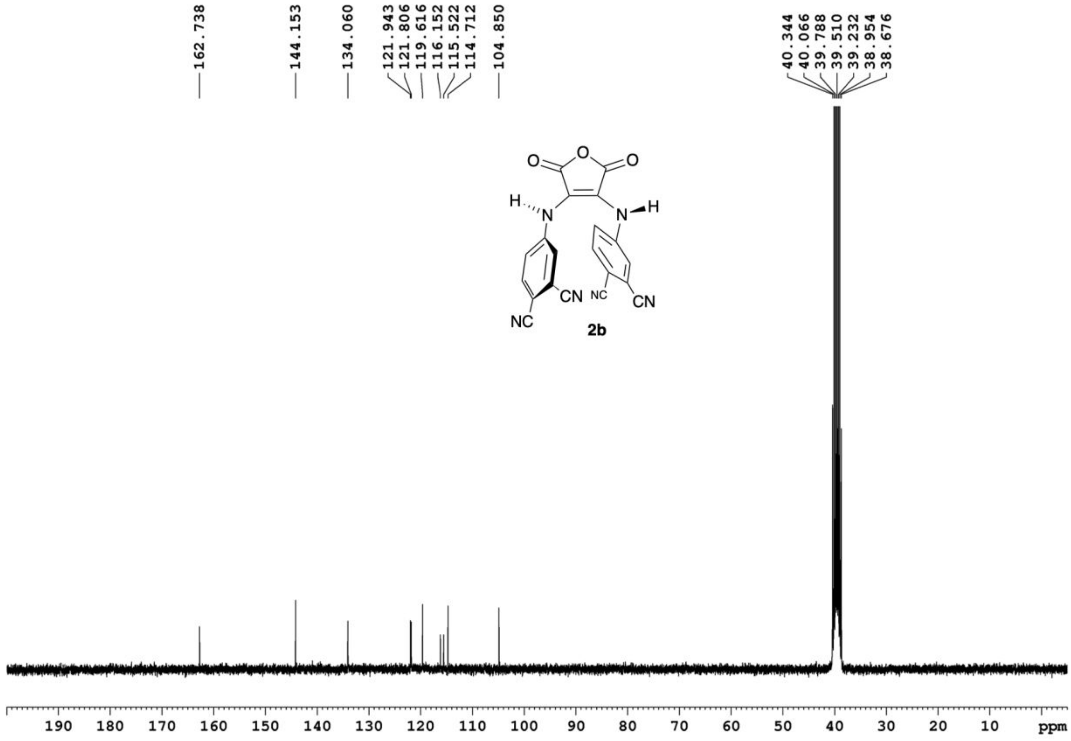

Figure S2.14. ^{13}C NMR (75 MHz) spectrum of anhydride**2b** in DMSO-d_6

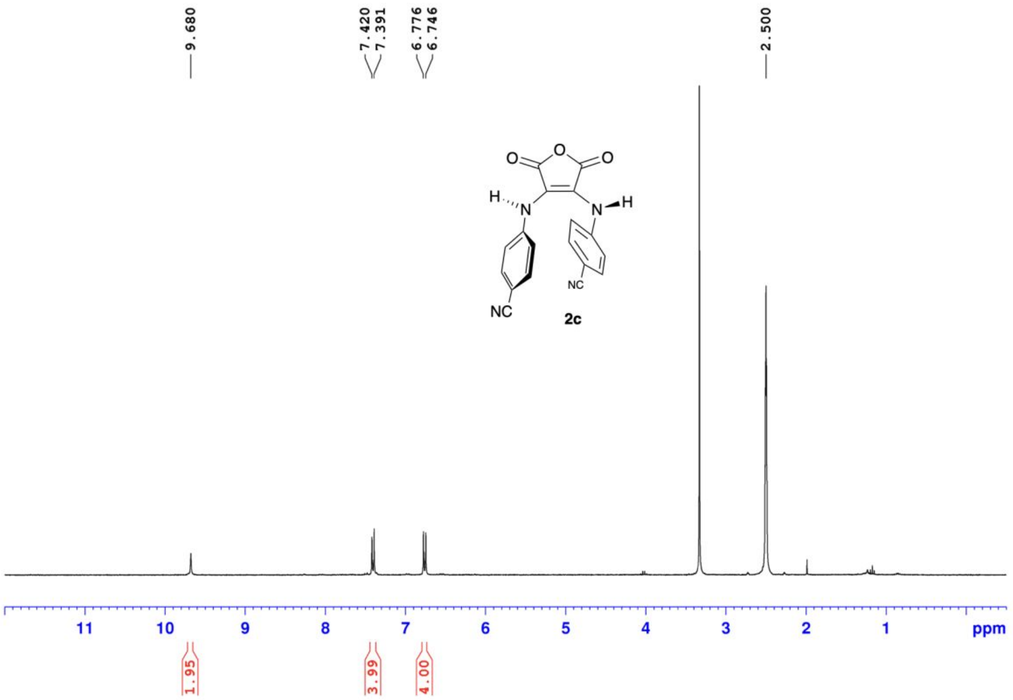

Figure S2.15. ^{1}H NMR (300 MHz) spectrum of anhydride **2c** in DMSO-d_6

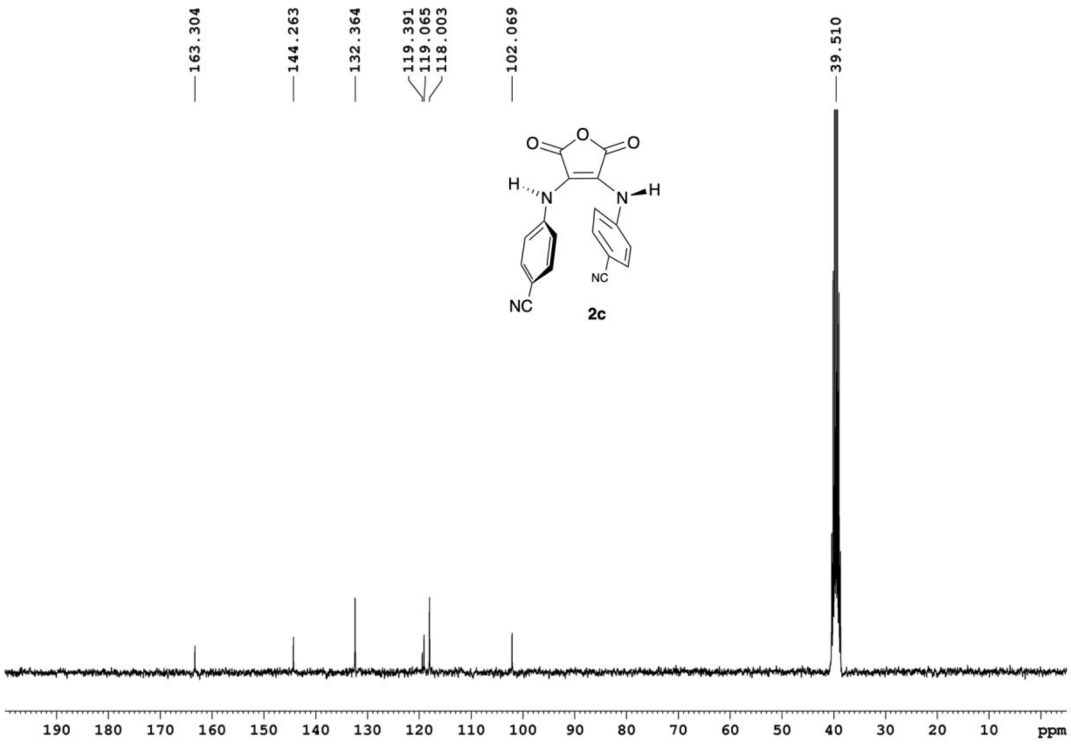

Figure S2.16. ^{13}C NMR (75 MHz) spectrum of anhydride**2c** in DMSO-d_6

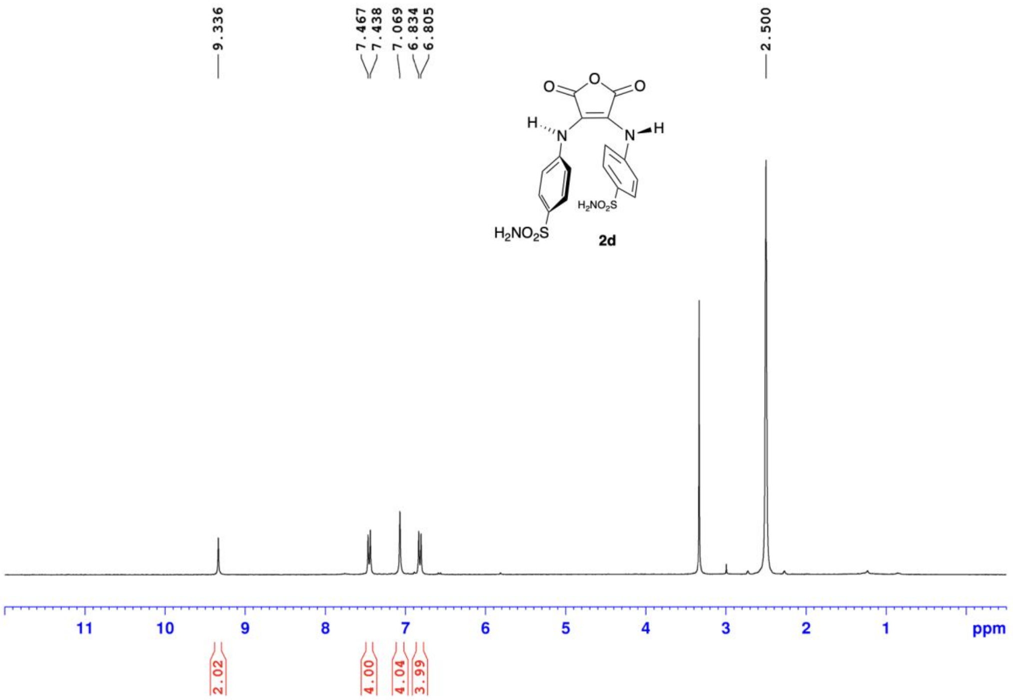

Figure S2.17. ^{1}H NMR (300 MHz) spectrum of anhydride **2d** in DMSO-d_6

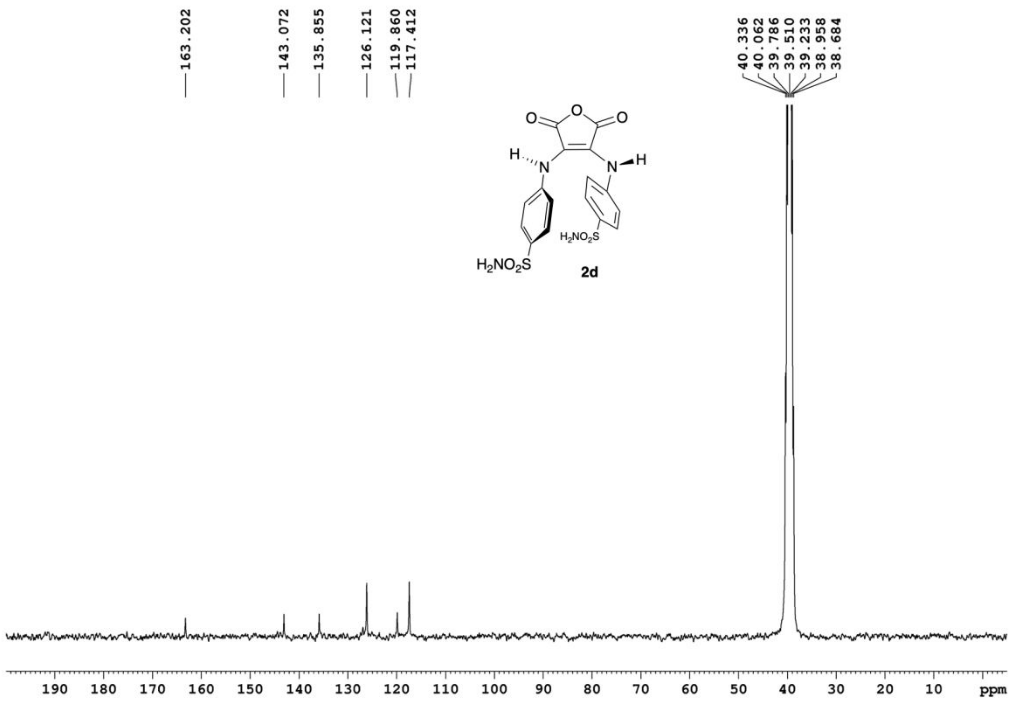

Figure S2.18. ^{13}C NMR (75 MHz) spectrum of anhydride **2d** in DMSO-d_6

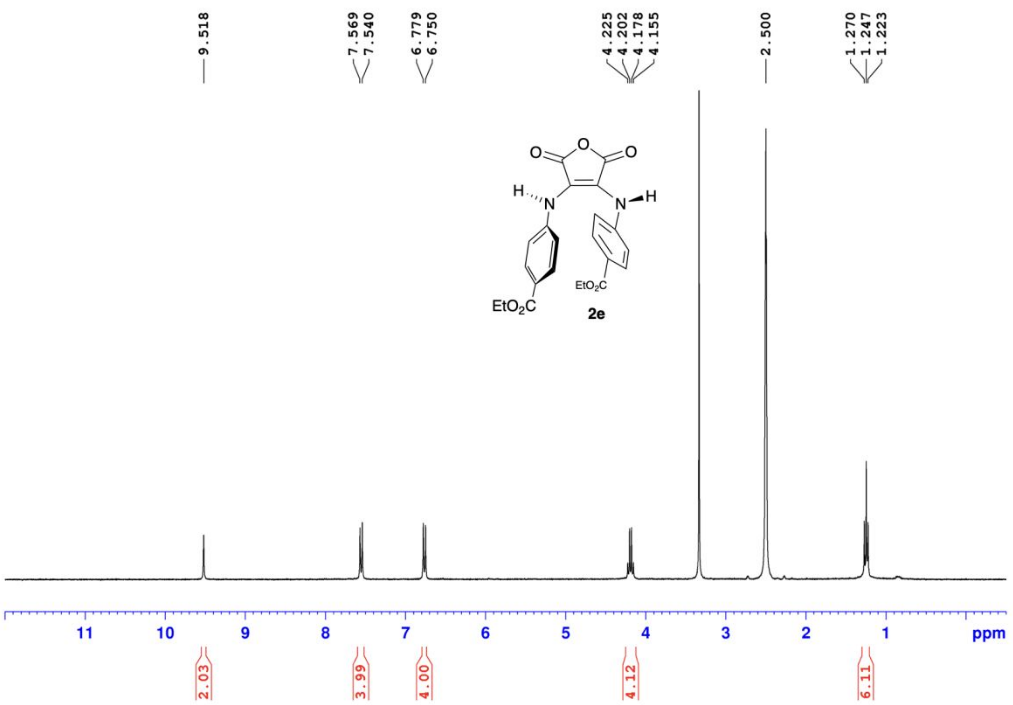

Figure S2.19. ^{1}H NMR (300 MHz) spectrum of anhydride **2e** in DMSO-d_6

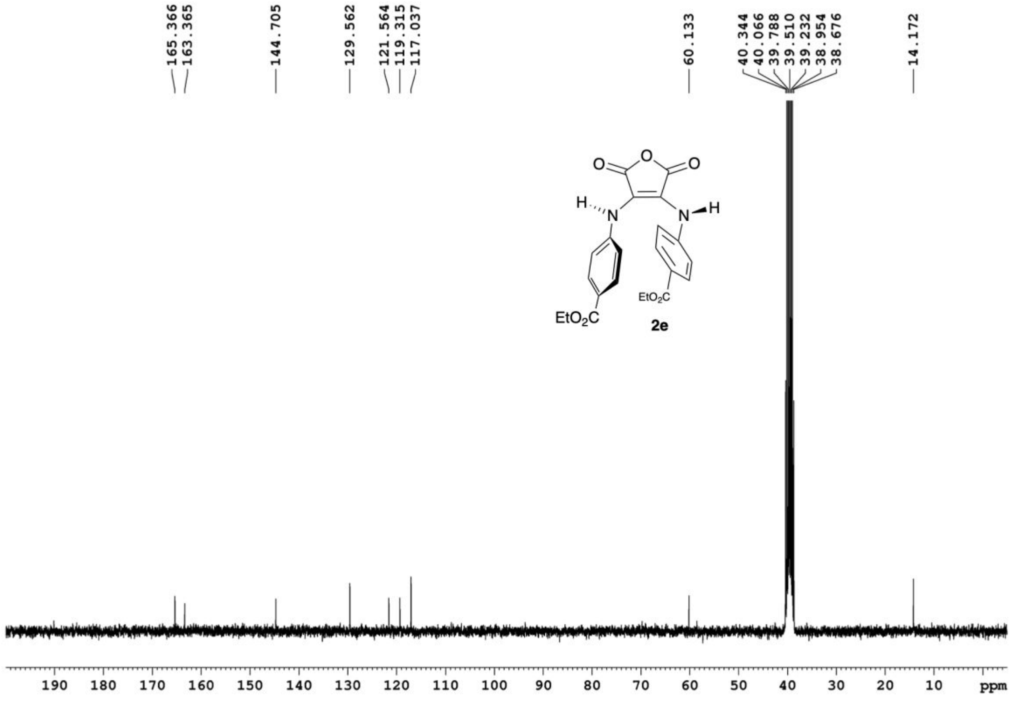

Figure S2.20. ^{13}C NMR (75 MHz) spectrum of anhydride **2e** in DMSO-d_6

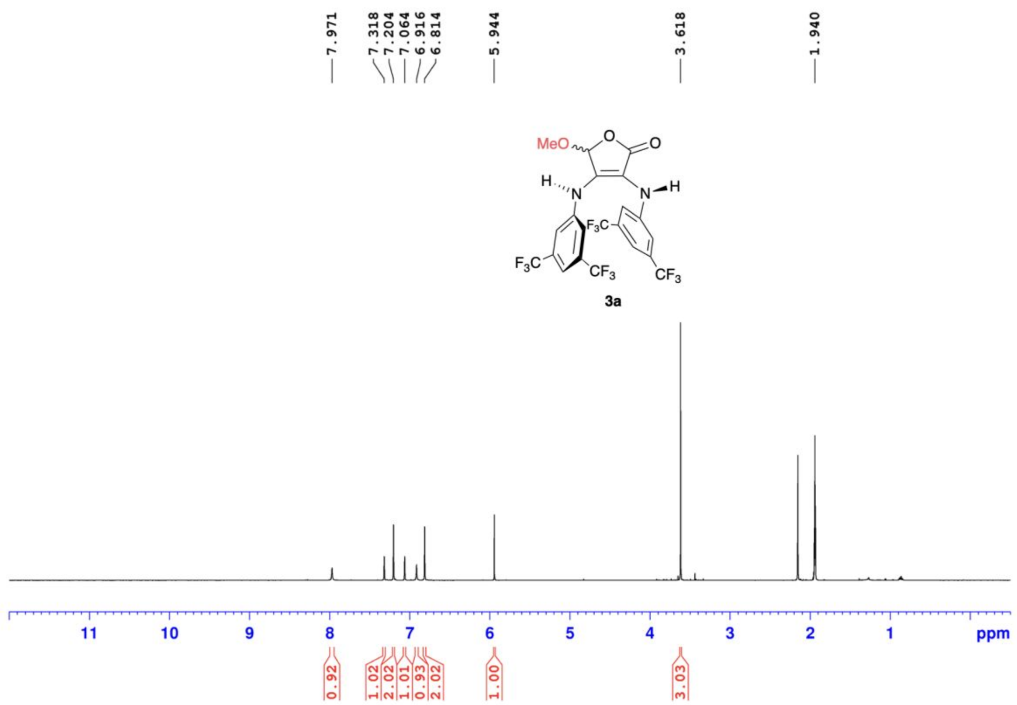

Figure S2.21. ^1H NMR (300 MHz) spectrum of **3a** in MeCN-d_3

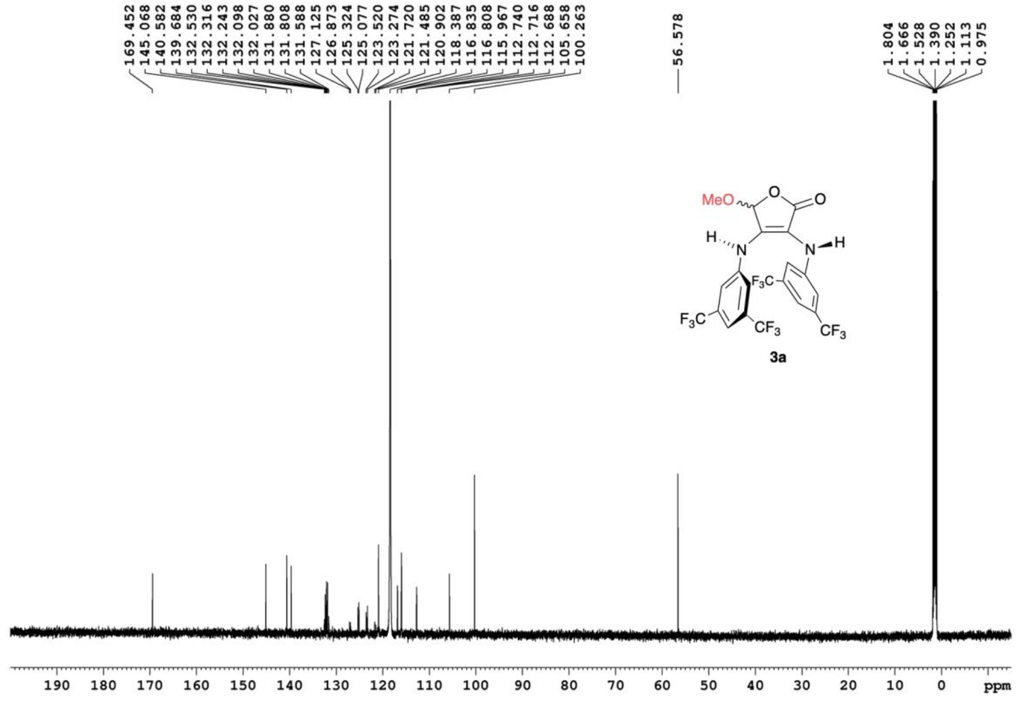

Figure S2.22. ^{13}C NMR (75 MHz) spectrum of **3a** in MeCN-d_3

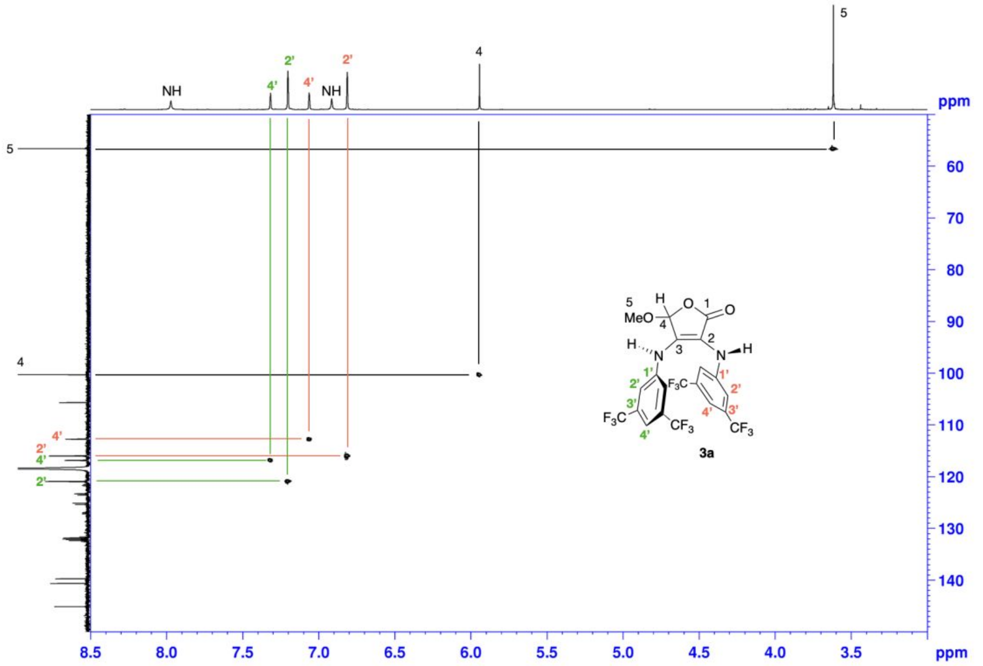

Figure S2.23: Heteronuclear single quantum correlation (HSQC, 600 MHz) spectrum of **3a** in MeCN-d_3 at 298 K**.**

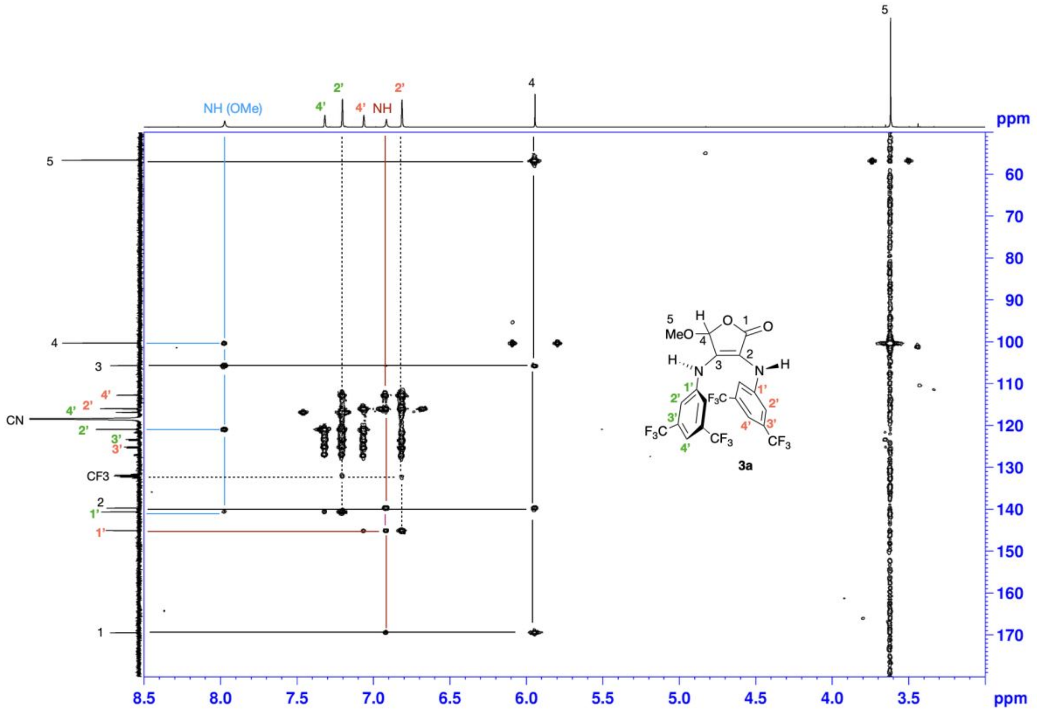

Figure S2.24: Heteronuclear multiple bond correlation (HMBC, 600 MHz) spectrum of **3a** in MeCN-d_3 at 298 K. Colored lines indicate several significant cross peaks.

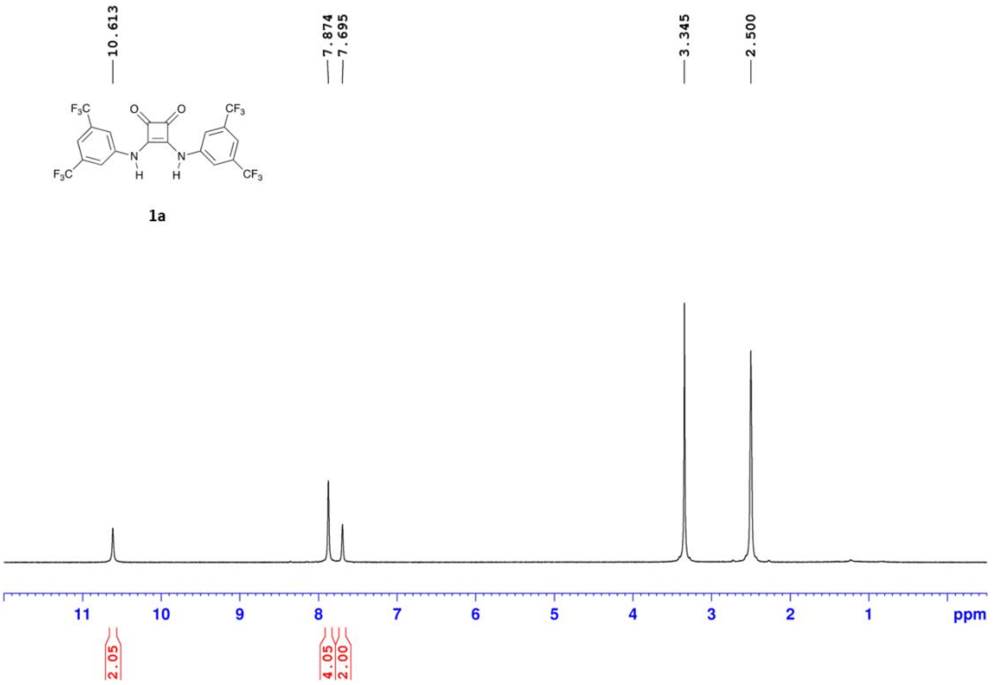

Figure S2.25 ^{1}H NMR (300 MHz) spectrum of AD-squaramide **1a** in DMSO-d_6

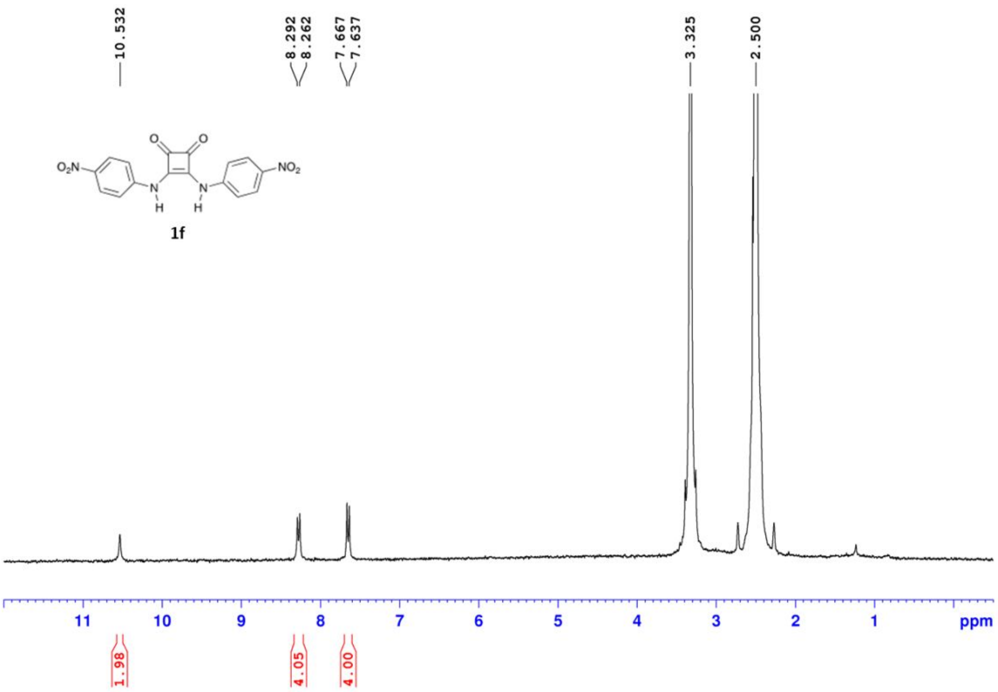

Figure S2.26 ^{1}H NMR (300 MHz) spectrum of AD-squaramide **1f** in DMSO-d_6

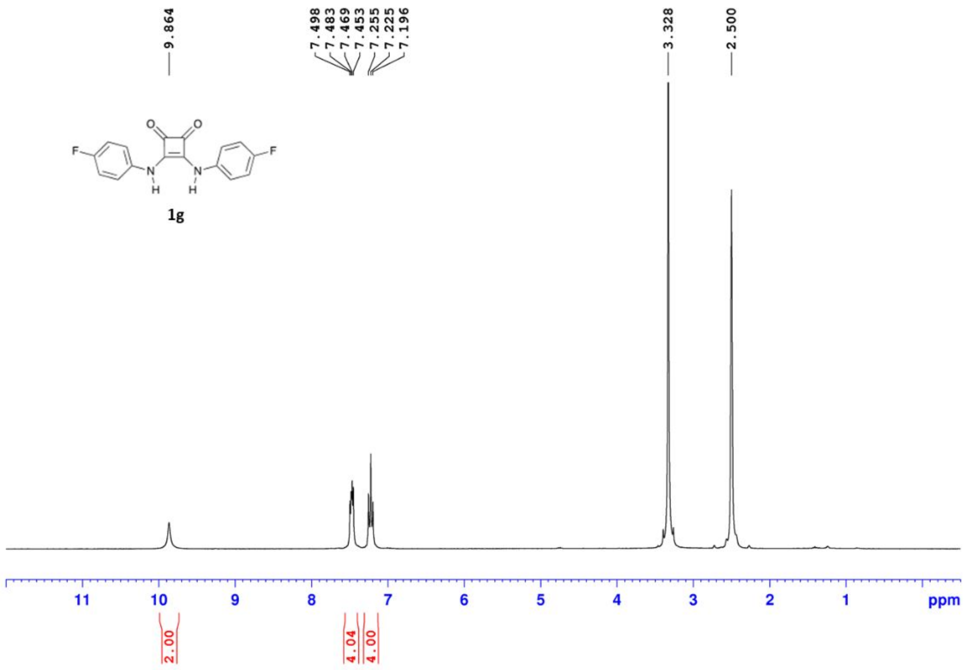

Figure S2.27 ^{1}H NMR (300 MHz) spectrum of AD-squaramide **1g** in DMSO-d_6

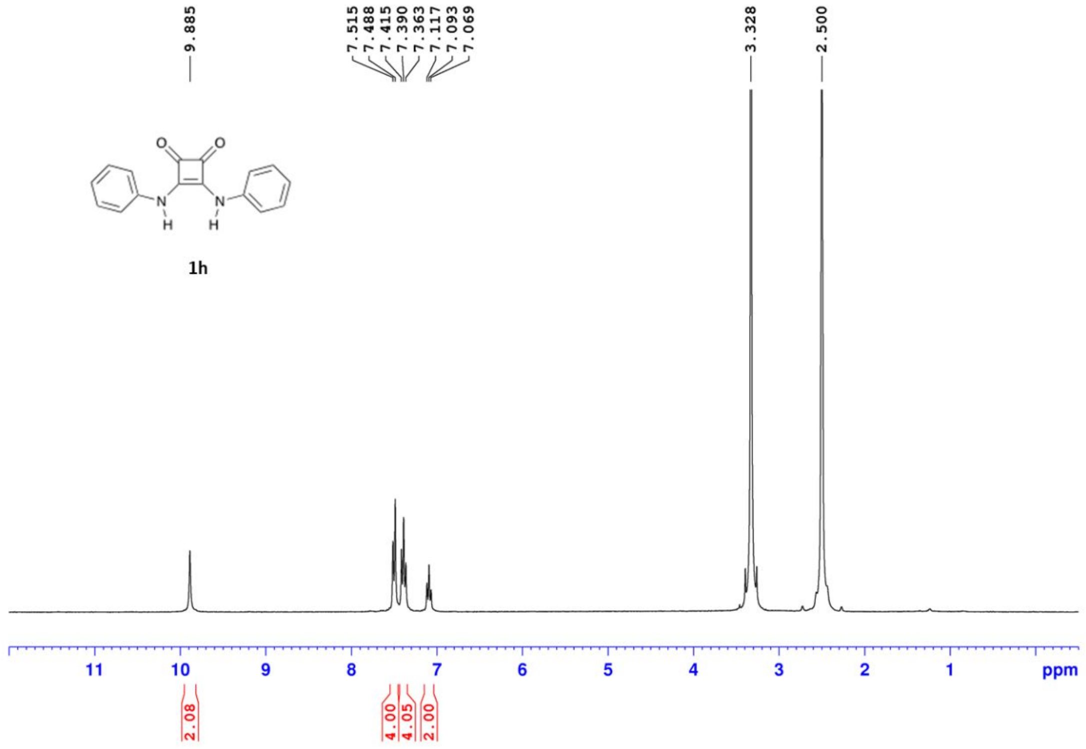

Figure S2.28 ^{1}H NMR (300 MHz) spectrum of AD-squaramide **1h** in DMSO-d_6

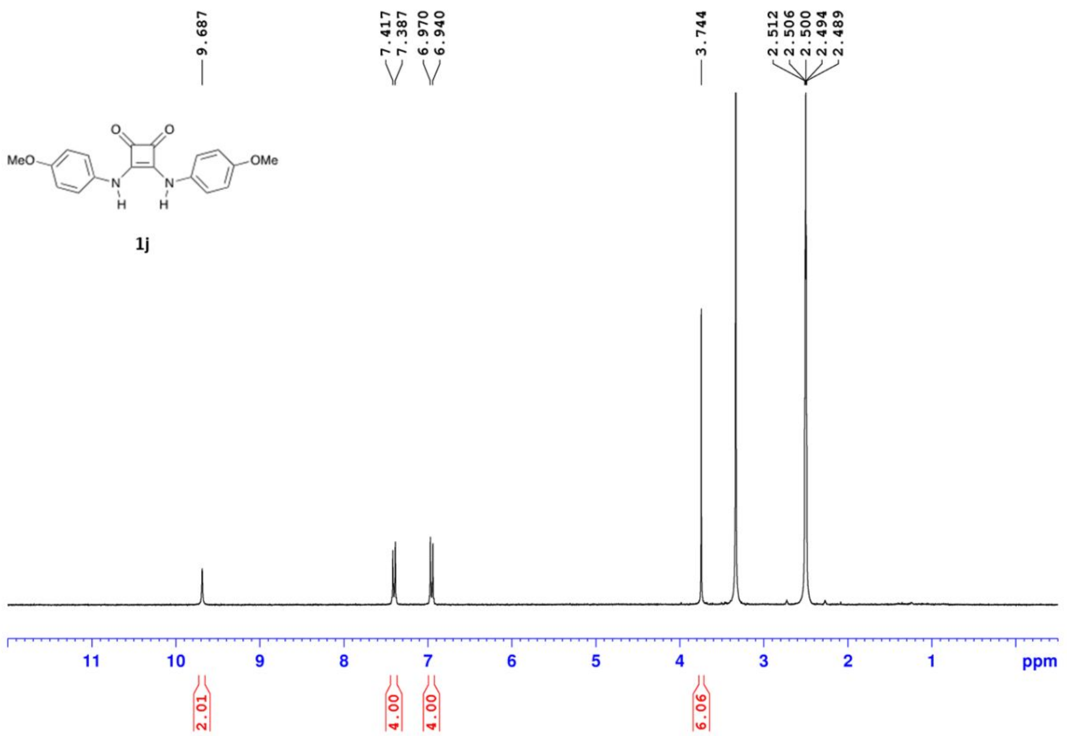

Figure S2.29 ^{1}H NMR (300 MHz) spectrum of AD-squaramide **1h** in DMSO-d_6

3. Photochemical reactions at analytical (^{1}H NMR) scale.

3.1 General photoreaction procedure

A 10^{-3} M solution of the AD-squaramide in DMSO-d_6 (0.5 mL) was introduced into a standard 5 mm ^{1}H NMR tube at room temperature (21 °C). Then, the test tube was irradiated with a commercial LED-UV flashlight (Alonefire X901; 10W, 365 nm) placed perpendicularly and at a distance of 10 cm from the tube. The irradiation periods were manually controlled by switching ON and OFF the flashlight. After each period, the sample was monitored by recording the ^{1}H NMR spectrum. The quantitation was achieved making use of 1,3,5-trioxane 5.0 × 10^{-4} M (5.20 ppm) as external calibration standard. The integrated peaks were used to construct the percentage plots. Initial reaction rates were calculated from these plots by fitting the linear portion of the curves. Curve fitting was performed with proFit 7.0.19 software (Quantum Soft).

3.2 Photoconversion of AD-squaramides 1a-j. Percentage plots.

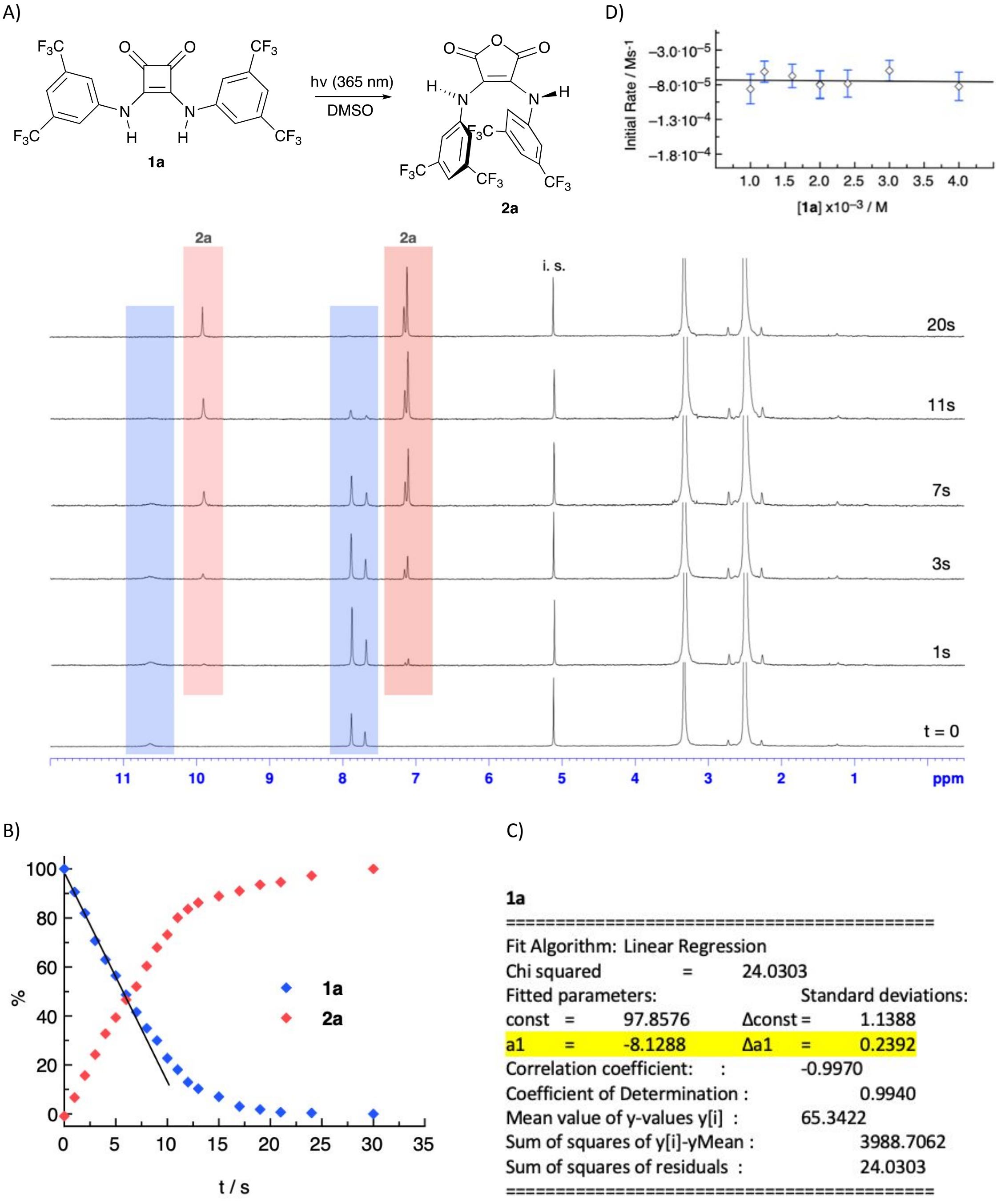

Figure S3.1 Photoreaction **1a** [1.0 × 10^{-3} M] to **2a.** (A) 1H NMR spectra recorded at indicated time periods (in seconds). (B) Time course percentage plots obtained by integration of the corresponding peaks and initial rate linear points. (C) Linear fitting results. (D) Zero-order rate-constant plot for **1a**.

A)

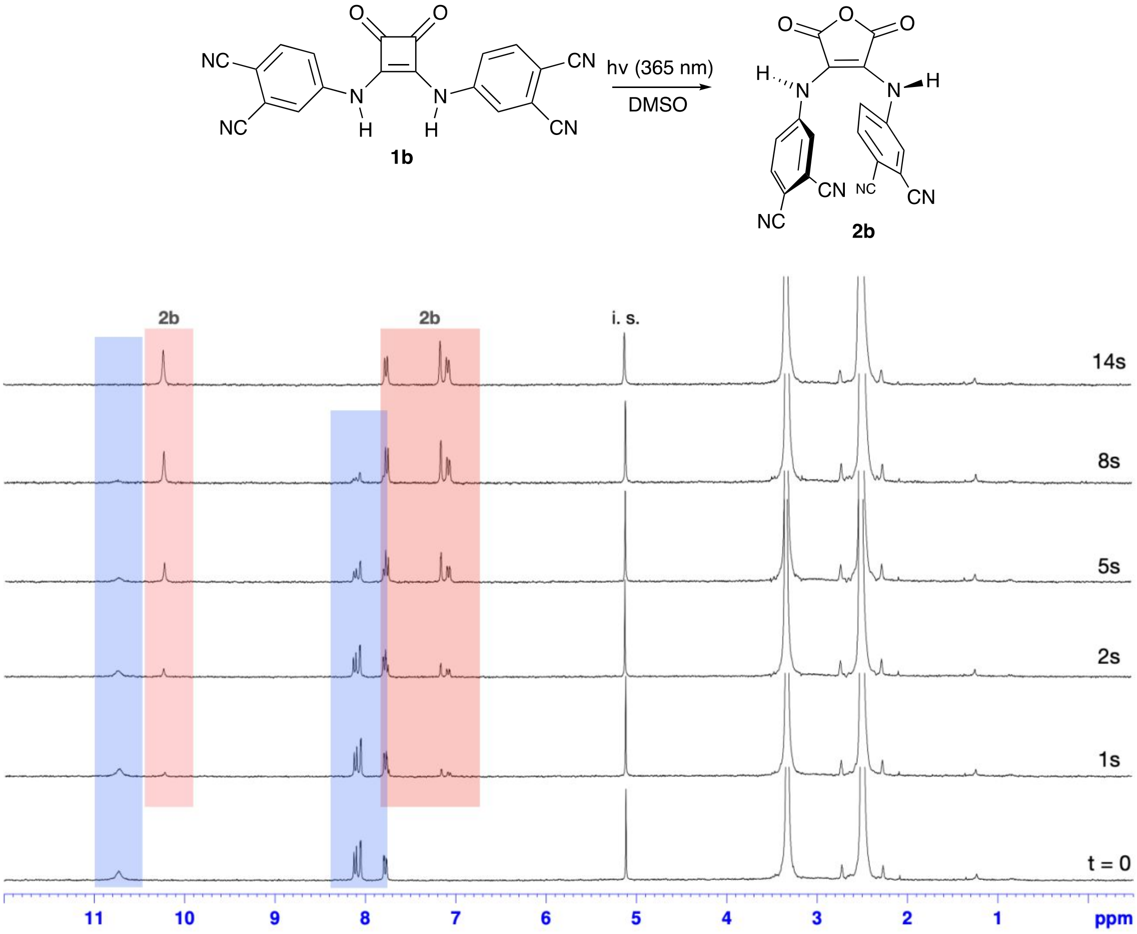

B)

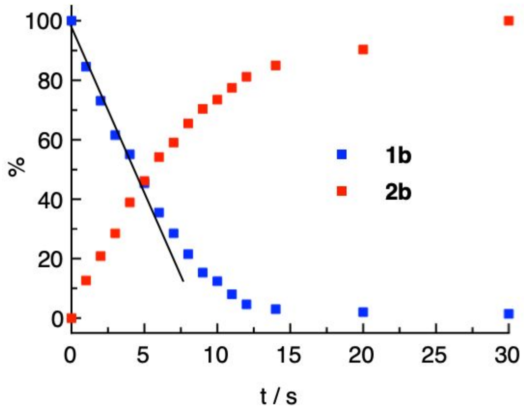

C)

1b

==

Fit Algorithm: Linear Regression

Chi squared	=	71.9465	
Fitted parameters:			Standard deviations:
const =	94.3840	Δconst =	1.9705
a1 =	-9.5610	Δa1 =	0.4139
Correlation coefficient: :		-0.9935	
Significance of correlation: :		3.3983 %	
Coefficient of Determination :			0.9871
Mean value of y-values y[i] :		56.1400	
Sum of squares of y[i]-yMean :			5556.7098
Sum of squares of residuals :			71.9465

==

Figure S3.2 Photoreaction **1b** [1.0×10^{-3} M] to **2b.** (A) ^{1}H NMR spectra recorded at indicated time periods (time i in seconds). (B) Time course percentage plots obtained by integration of the corresponding peaks and initial rate linear points. (C) Linear fitting results.

A)

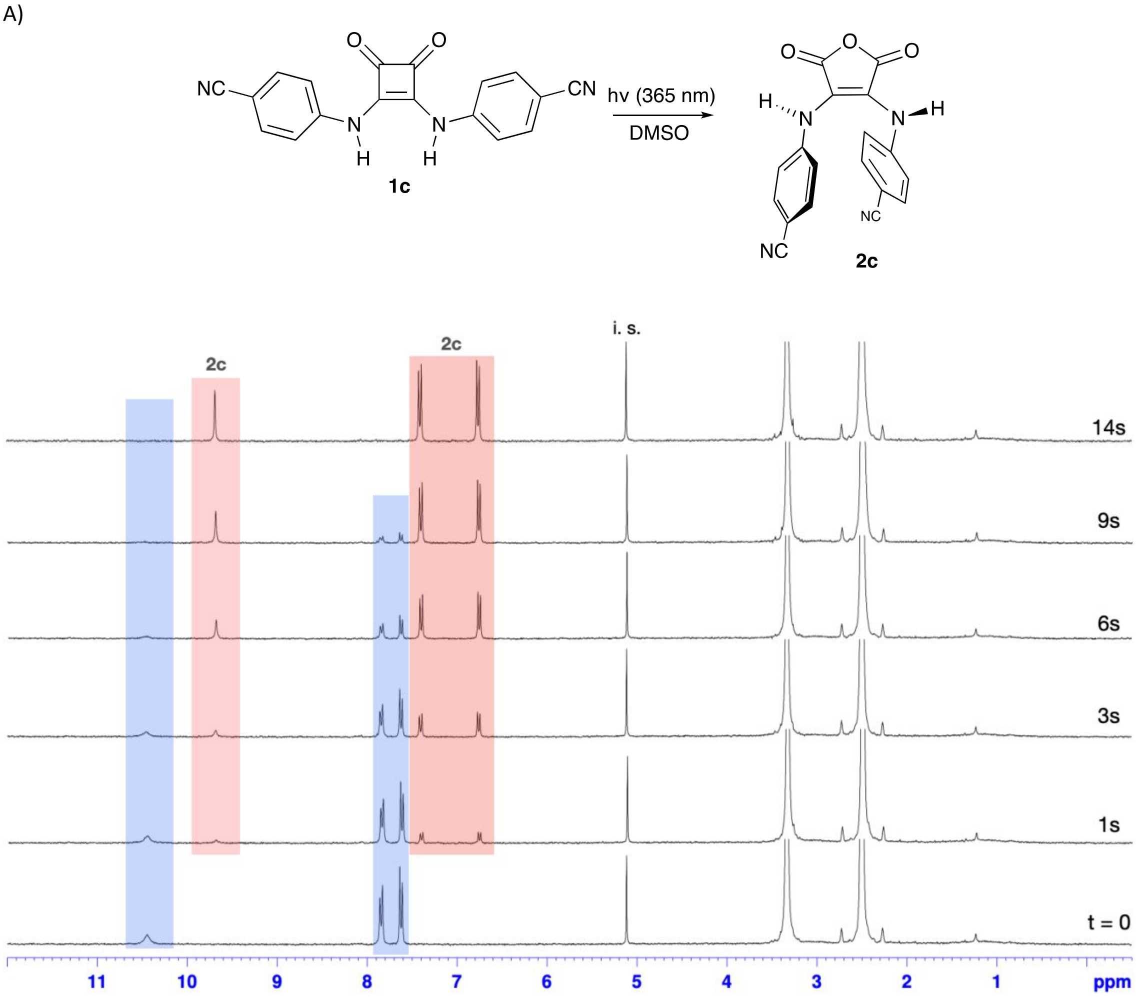

B)

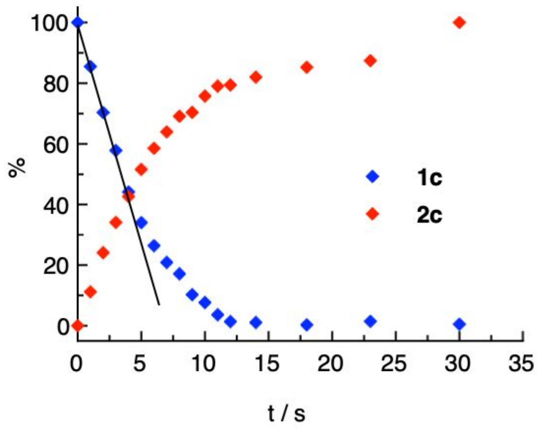

C)

1c

==

Fit Algorithm: Linear Regression

Chi squared =	32.8943		
Fitted parameters:		Standard deviations:	
const =	96.6095	Δconst =	2.0755
a1 =	-10.6611	Δa1 =	0.6855
Correlation coefficient: :		-0.9918	
Significance of correlation: :		11.8305 %	
Coefficient of Determination :			0.9837
Mean value of y-values y[i] :		69.9567	
Sum of squares of y[i]-yMean :			2021.9437
Sum of squares of residuals :			32.8943

==

Figure S3.3 Photoreaction **1c** [1.0 × 10^{-3} M] to **2c.** (A) ^{1}H NMR spectra recorded at indicated time periods (time in seconds). (B) Time course percentage plots obtained by integration of the corresponding peaks and initial rate linear points. (C) Linear fitting results.

A)

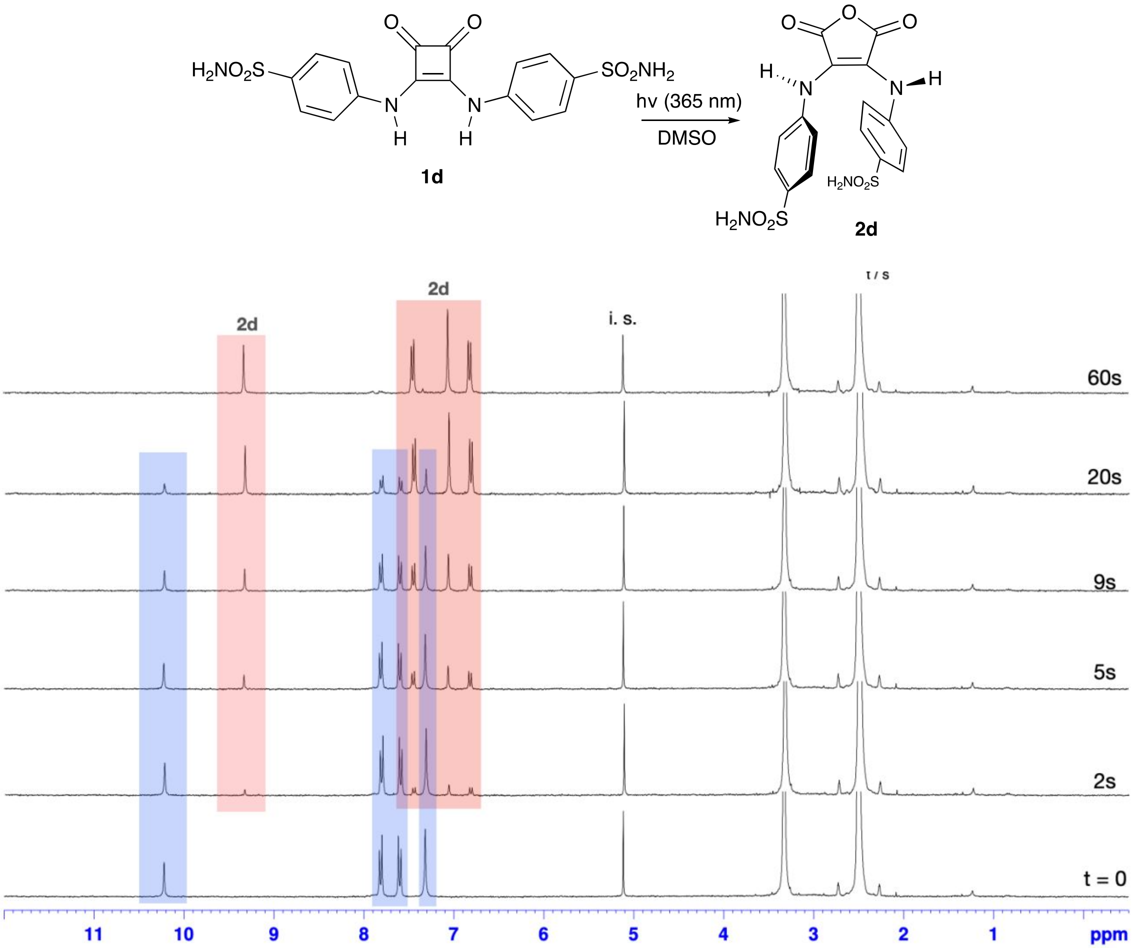

B)

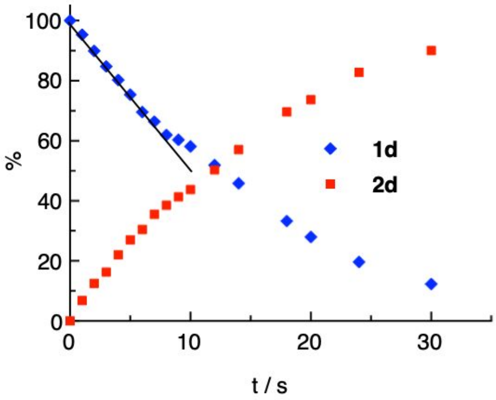

C)

1d

==

Fit Algorithm: Linear Regression

Chi squared = 2.8688

Fitted parameters: Standard deviations:

const = 99.6493 Δconst = 0.3935

a1 = -4.8173 Δa1 = 0.082646

Correlation coefficient: : -0.9990

Significance of correlation: : 3.3277 %

Coefficient of Determination : 0.9979

Mean value of y-values y[i] : 80.3800

Sum of squares of y[i]-yMean : 1395.2708

Sum of squares of residuals : 2.8688

==

Figure S3.4 Photoreaction **1d** [1.0 × 10^{-3} M] to **2d.** (A) ^{1}H NMR spectra recorded at indicated time periods (time in seconds). (B) Time course percentage plots obtained by integration of the corresponding peaks and initial rate linear points. (C) Linear fitting results.

A)

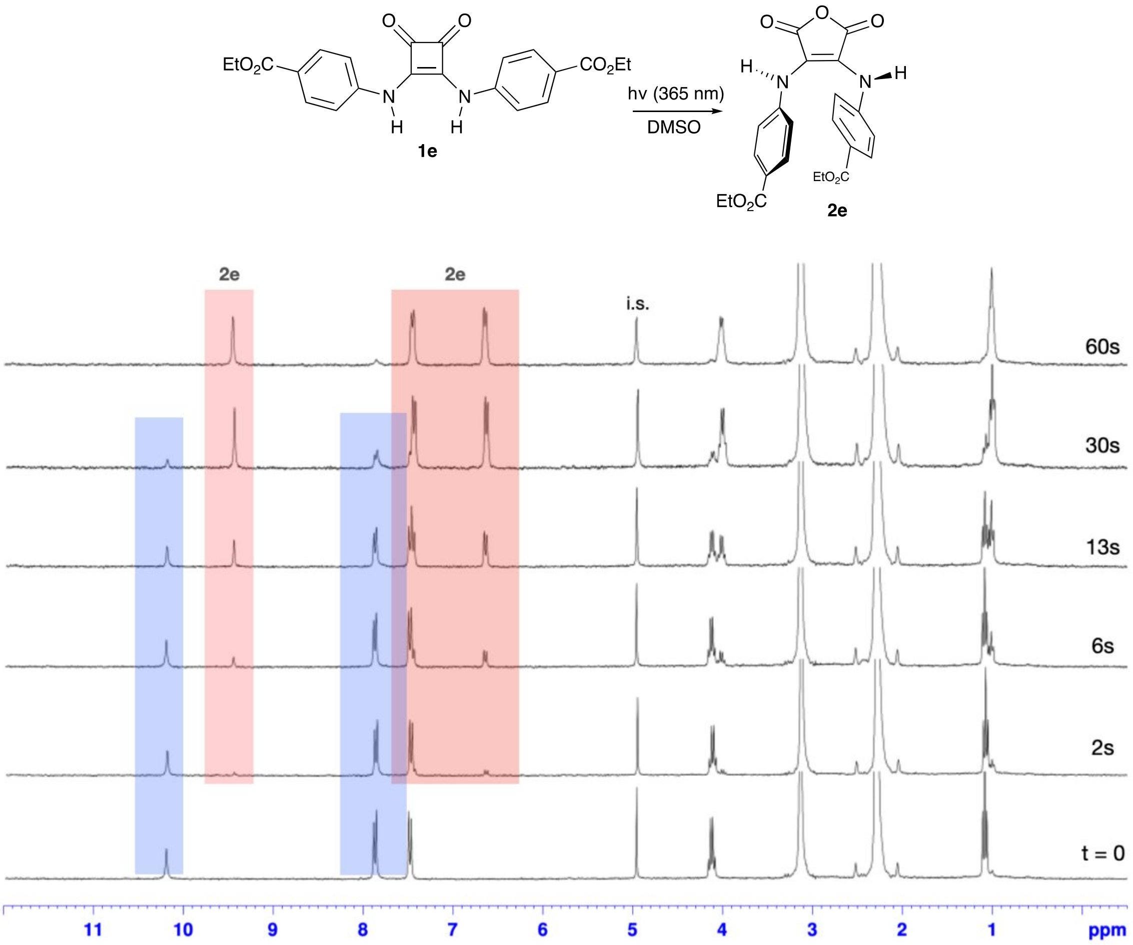

B)

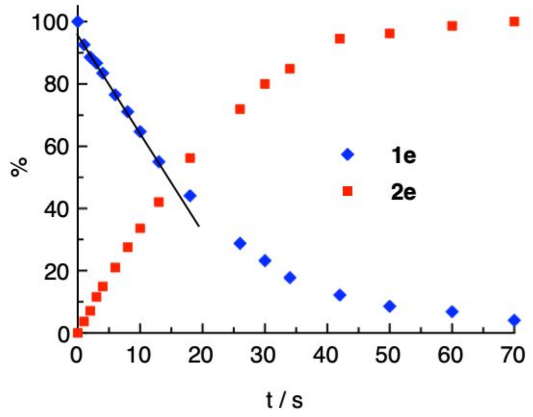

C)

1e

==

Fit Algorithm: Linear Regression

Chi squared = 15.3350

Fitted parameters: Standard deviations:

const = 96.8326 Δconst = 0.7953

a1 = -3.2507 Δa1 = 0.1194

Correlation coefficient: : -0.9953

Significance of correlation: : 3.3749 %

Coefficient of Determination : 0.9906

Mean value of y-values y[i] : 79.8567

Sum of squares of y[i]-yMean : 1637.9662

Sum of squares of residuals : 15.3350

==

Figure S3.5 Photoreaction **1e** [1.0 × 10^{-3} M] to **2e.** (A) ^{1}H NMR spectra recorded at indicated time periods (time in seconds). (B) Time course percentage plots obtained by integration of the corresponding peaks and initial rate linear points. (C) Linear fitting results.

A)

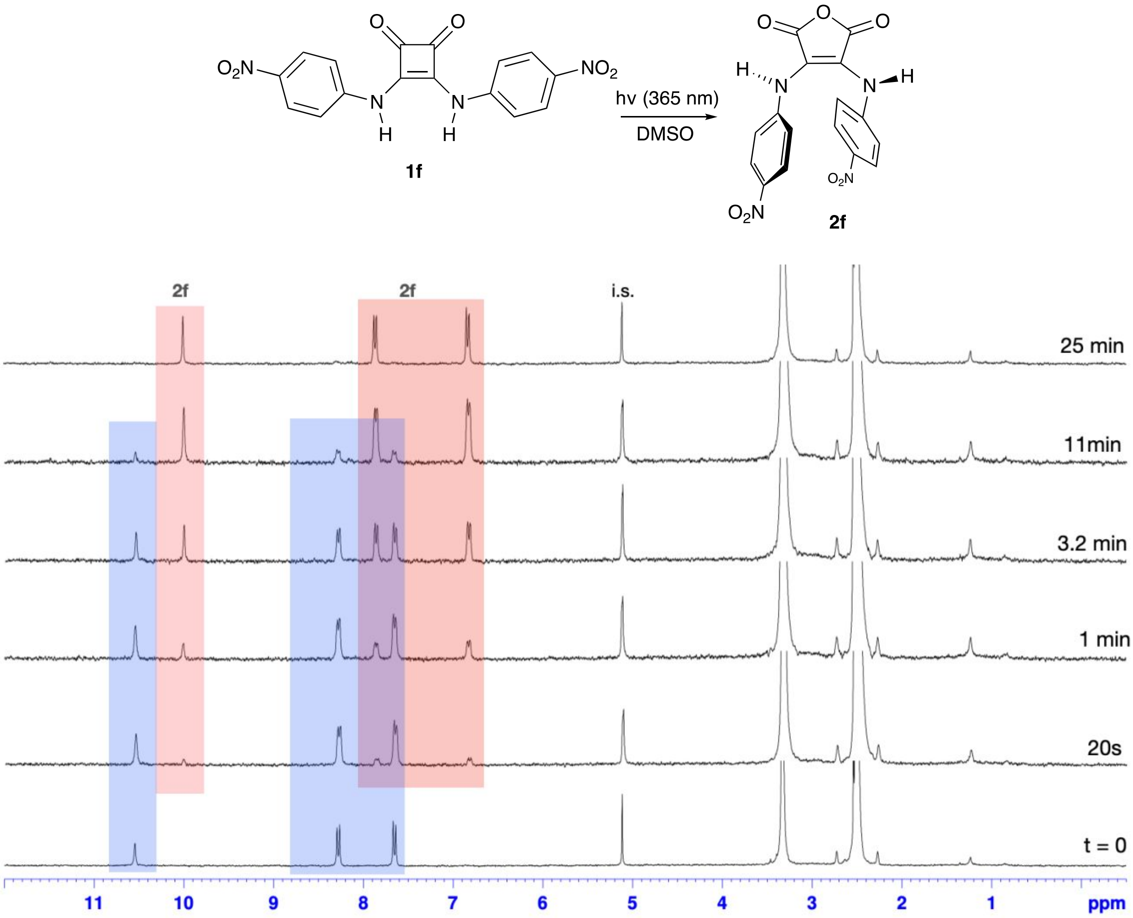

B)

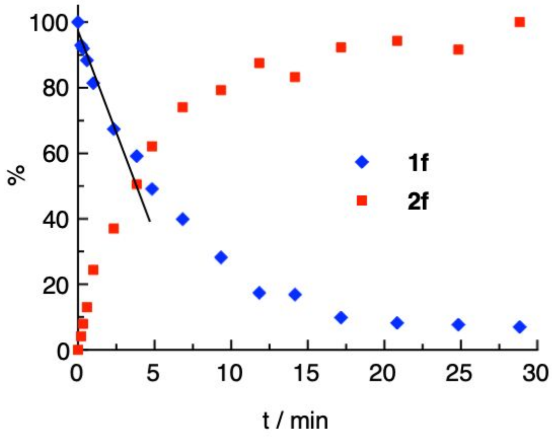

C)

1f

```
==========================================
Fit Algorithm:  Linear Regression
Chi squared          =        7.1060
Fitted parameters:                    Standard deviations:
const   =        98.2351        Δconst =        1.0894
a1      =        -0.2874        Δa1    =        0.033245
Correlation coefficient:    :         -0.9805
Significance of correlation:  :       18.8017 %
Coefficient of Determination :                 0.9614
Mean value of y-values y[i]  :        90.9360
Sum of squares of y[i]-yMean :                 184.0893
Sum of squares of residuals  :                 7.1060
==========================================
```

Figure S3.6 Photoreaction **1f** [1.0 × 10^{-3} M] to **2f.** (A) ^{1}H NMR spectra recorded at indicated time periods (time in minutes). (B) Time course percentage plots obtained by integration of the corresponding peaks and initial rate linear points. (C) Linear fitting results.

A)

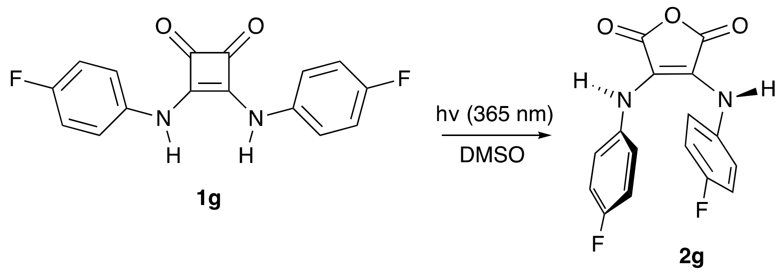

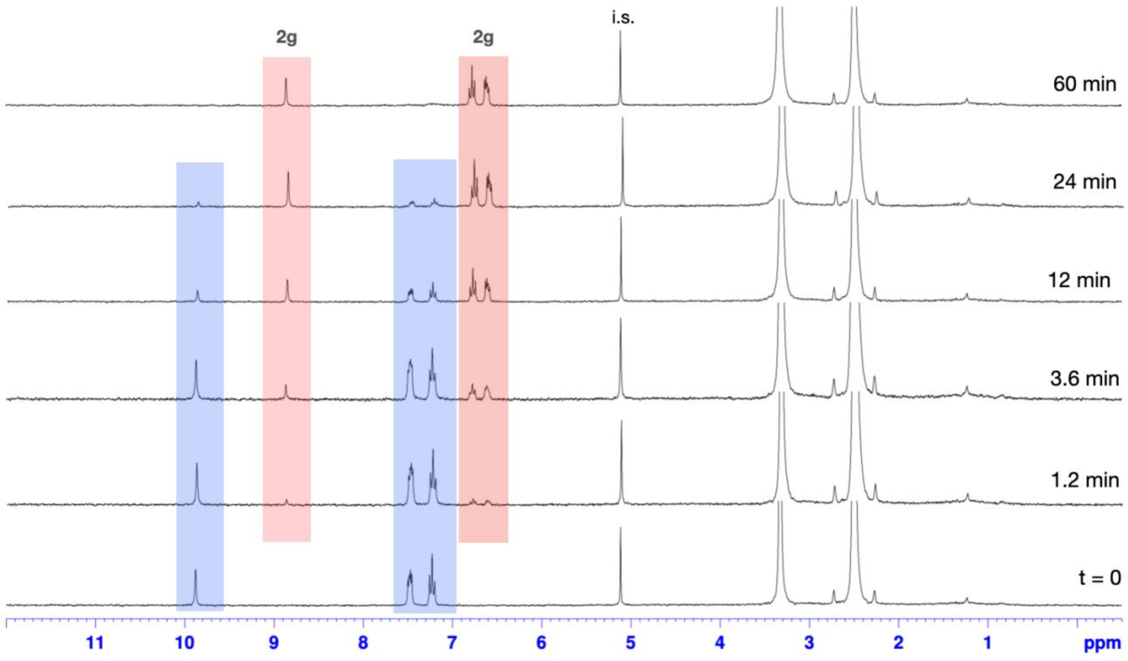

B)

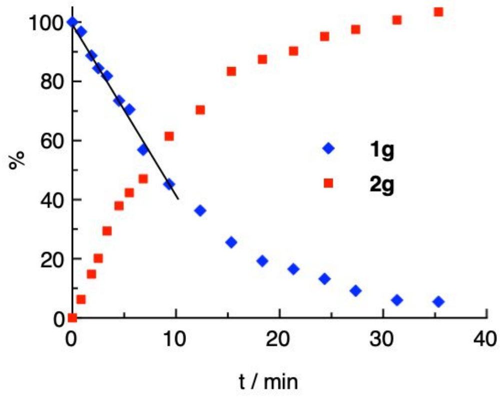

C)

1g

==

Fit Algorithm: Linear Regression

Chi squared = 9.1219

Fitted parameters: Standard deviations:

const = 99.8849 Δconst = 0.9023

a1 = -0.093229 Δa1 = 0.0046916

Correlation coefficient: : -0.9937

Significance of correlation: : 7.6930 %

Coefficient of Determination : 0.9875

Mean value of y-values y[i] : 85.1014

Sum of squares of y[i]-yMean : 729.5415

Sum of squares of residuals : 9.1219

==

Figure S3.7 Photoreaction **1g** [1.0 × 10^{-3} M] to **2g.** (A) ^{1}H NMR spectra recorded at indicated time periods (time in minutes). (B) Time course percentage plots obtained by integration of the corresponding peaks and initial rate linear points. (C) Linear fitting results.

A)

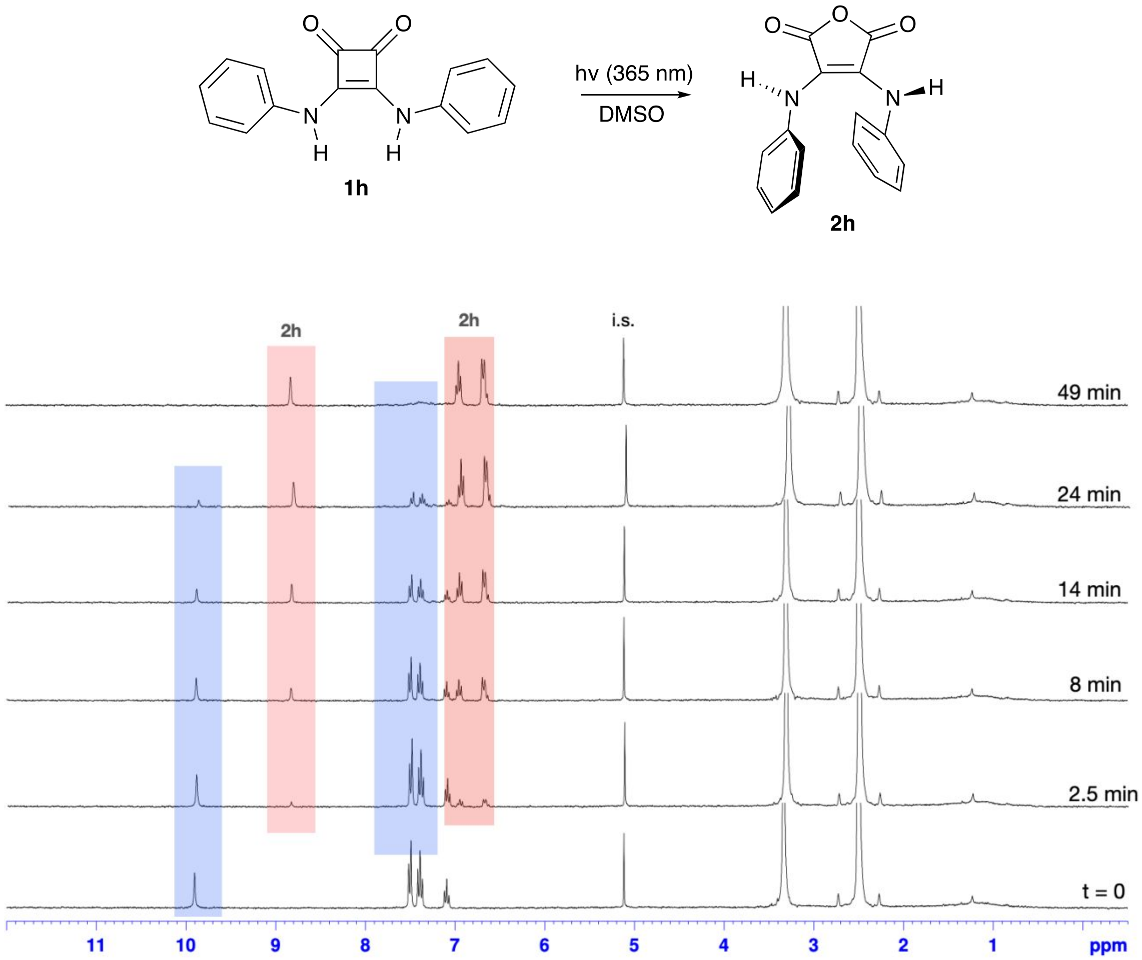

B)

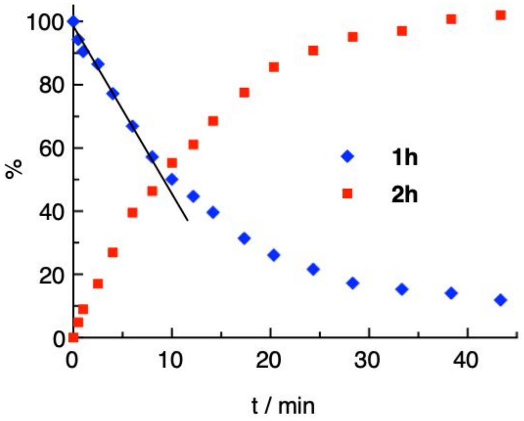

C)

1h

==

Fit Algorithm: Linear Regression
Chi squared = 12.8809
Fitted parameters: Standard deviations:
const = 97.9324 Δconst = 1.0909
a1 = -0.085958 Δa1 = 0.0057737
Correlation coefficient: : -0.9911
Significance of correlation: : 11.8504 %
Coefficient of Determination : 0.9823
Mean value of y-values y[i] : 85.8983
Sum of squares of y[i]-yMean : 726.6295
Sum of squares of residuals : 12.8809

==

Figure S3.8 Photoreaction **1h** [1.0 × 10^{-3} M] to **2h.** (A) ^{1}H NMR spectra recorded at indicated time periods (time in minutes). (B) Time course percentage plots obtained by integration of the corresponding peaks and initial rate linear points. (C) Linear fitting results.

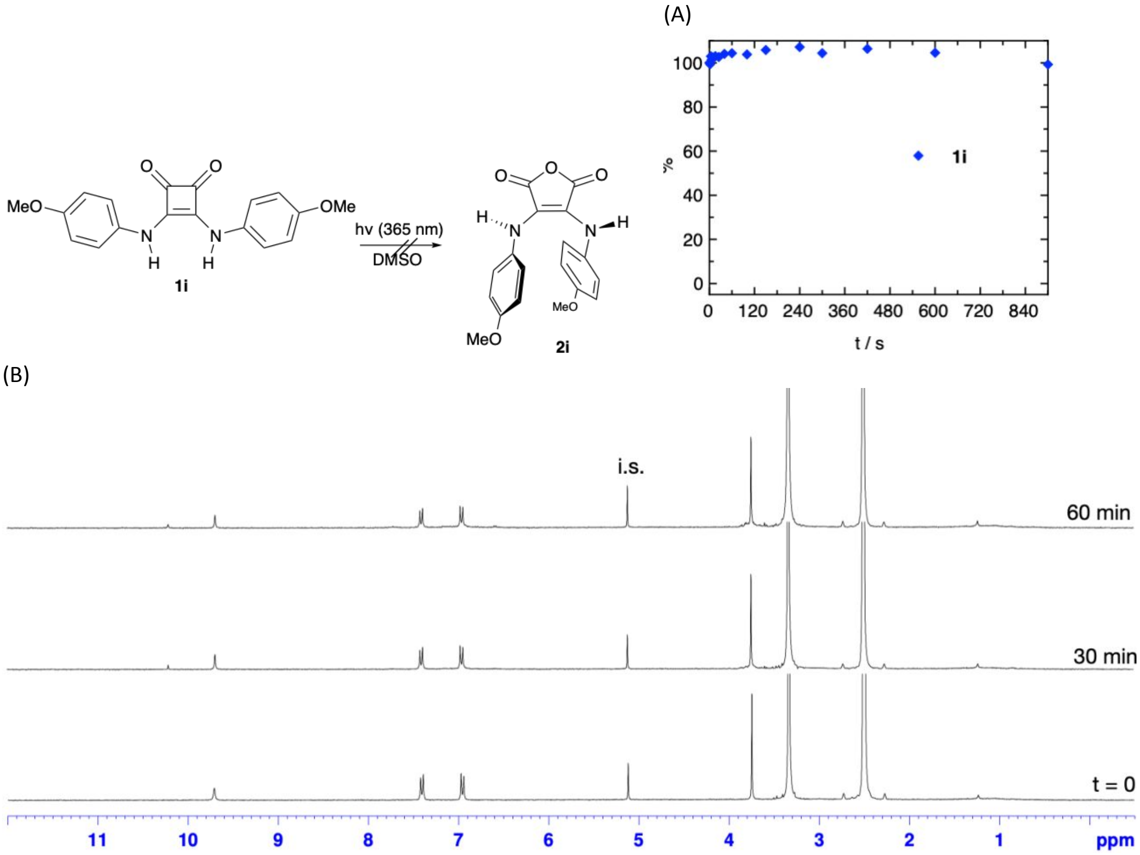

Figure S3.9. UV Irradiation of **1i** [1.0 × 10^{-3} M]**.** (A) Time course percentage plots obtained by integration of the corresponding peaks of **1i**. (B) ^{1}H NMR spectra recorded at indicated time periods (time in seconds).

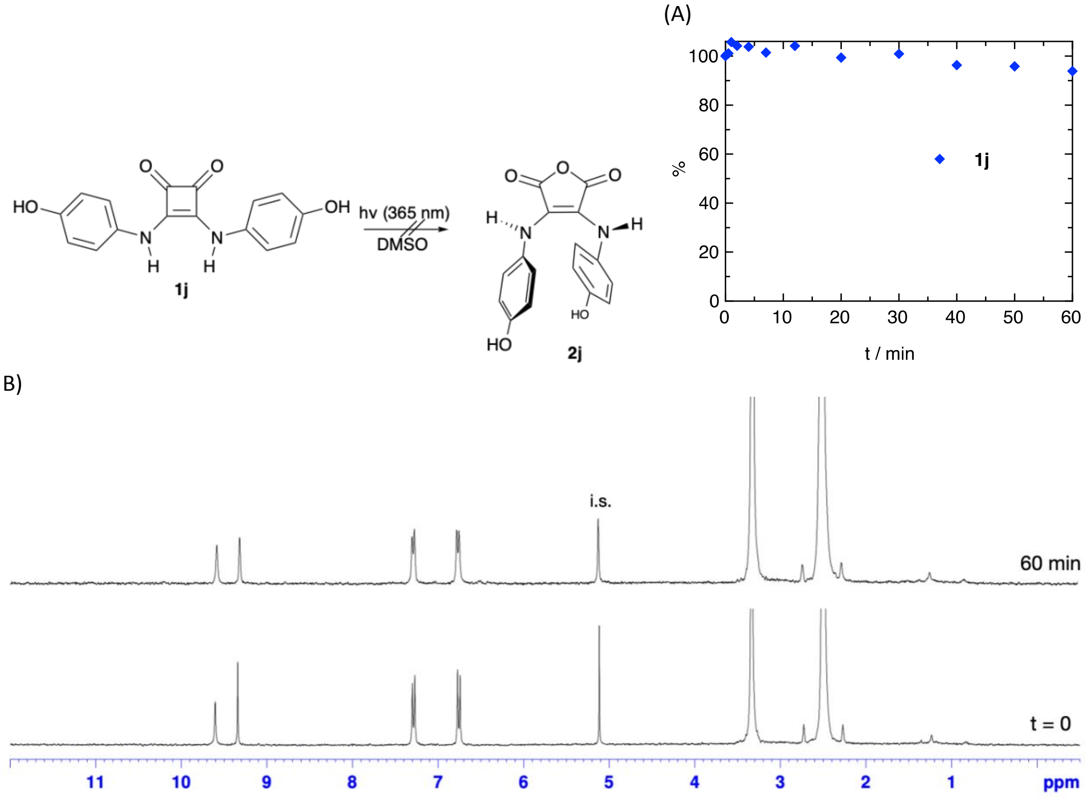

Figure S3.10 UV Irradiation of **1j** [1.0 × 10^{-3} M]**.** (A) Time course percentage plots obtained by integration of the corresponding peaks. (B) ^{1}H NMR spectra recorded at indicated time periods (time in minutes).

3.3. Influence of added anions and cations on the photoconversion

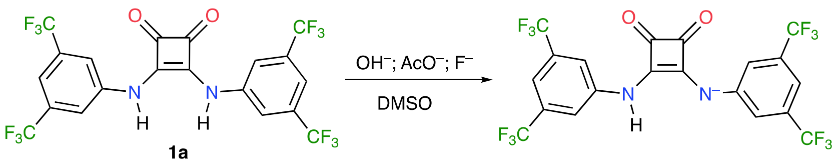

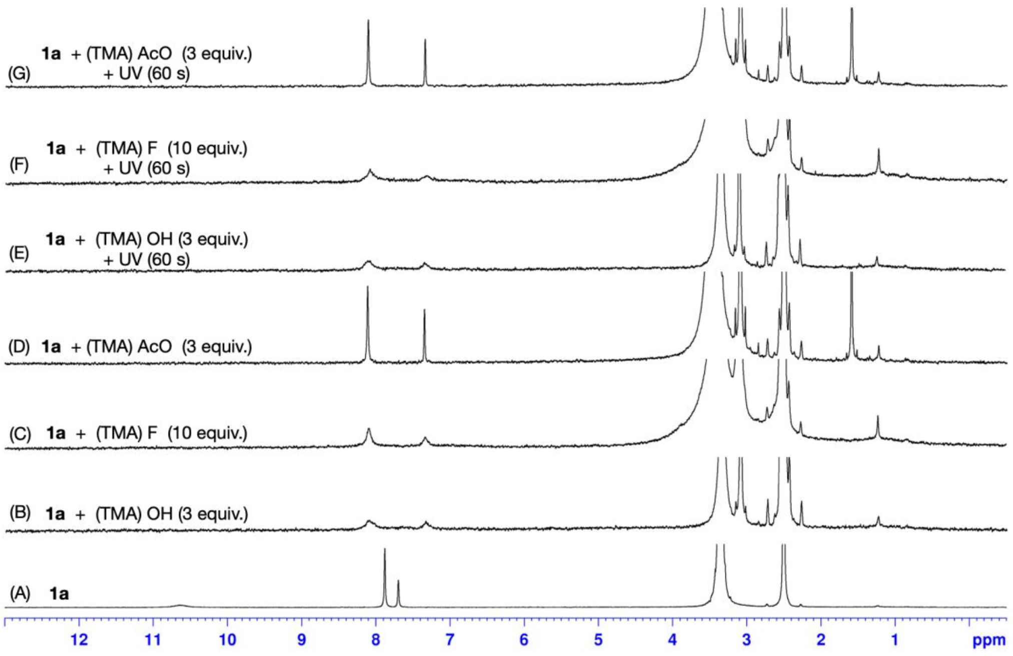

Figure S3.11. (A) ^{1}H NMR spectrum of **1a** [1.0×10^{-3} M] in DMSO-d_6. (B)(C)(D) ^{1}H NMR spectra of **1a** monoanion[S1] formed by adding OH^-; AcO^-; or F anions as tetraalkylammonium salts, respectively, to the initial **1a** sample. These anions deprotonate AD-squaramide **1a**. (E)(F)(G) ^{1}H NMR spectra showing the inhibitory effect of the ionizing anions on the photoconversion of **1a**. Irradiation time is indicated in seconds.

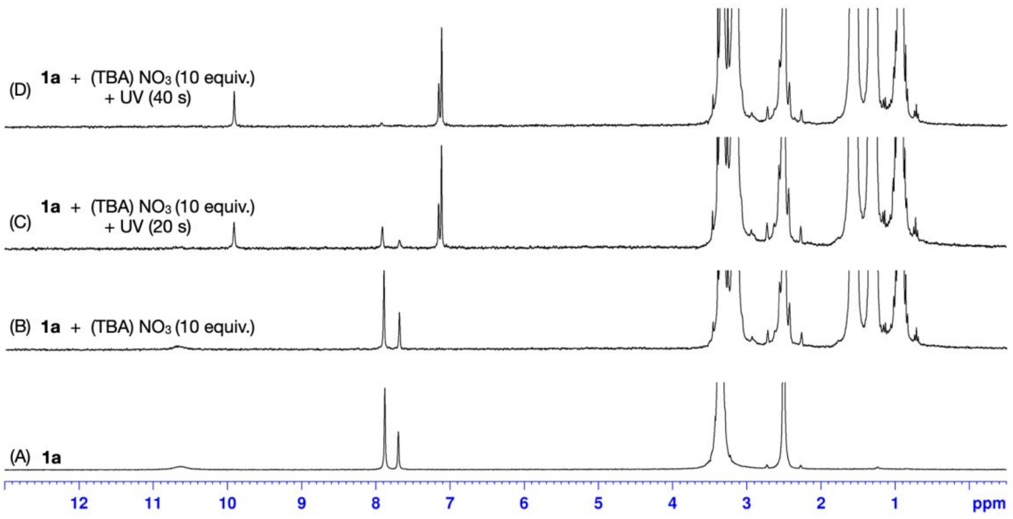

Figure S3.12. (A) ^{1}H NMR spectrum of **1a** [1.0×10^{-3} M] in DMSO-d_6. (B) ^{1}H NMR spectrum of **1a** in the presence of a excess of (TBA)NO_3, a non-ionising anion, to the initial **1a** sample. (C)(D) ^{1}H NMR spectra showing the time course of the photoconversion in the presence of a non-ionizing anion (NO_3^-).

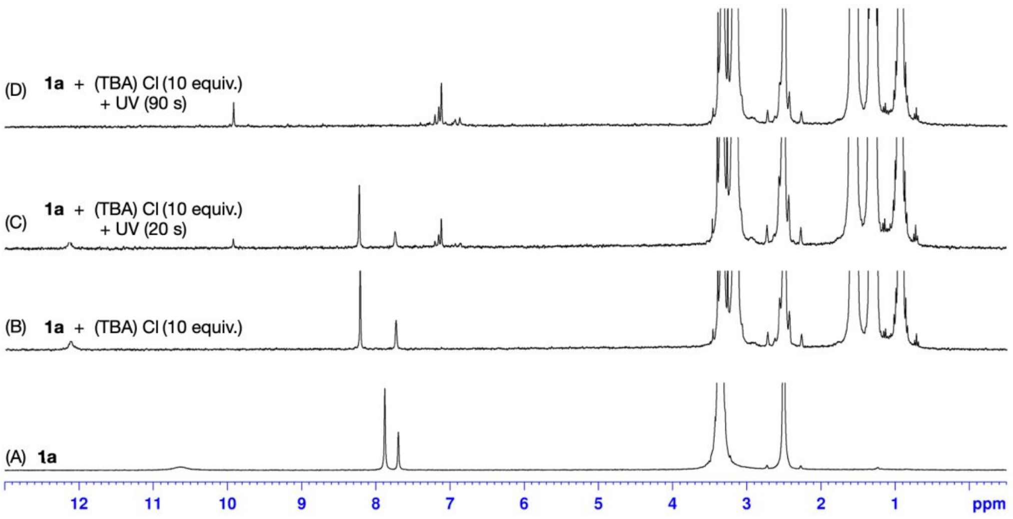

Figure S3.13. (A) 1H NMR spectrum of **1a** [1.0×10^{-3} M] in DMSO-d_6. (B) ^{1}H NMR spectrum of **1a** in the presence of a excess of (TBA)Cl , a non-ionising anion, to the initial **1a** sample. (C)(D) ^{1}H NMR spectra showing the time course of the photoconversion in the presence of a non-ionizing anion (Cl^-).

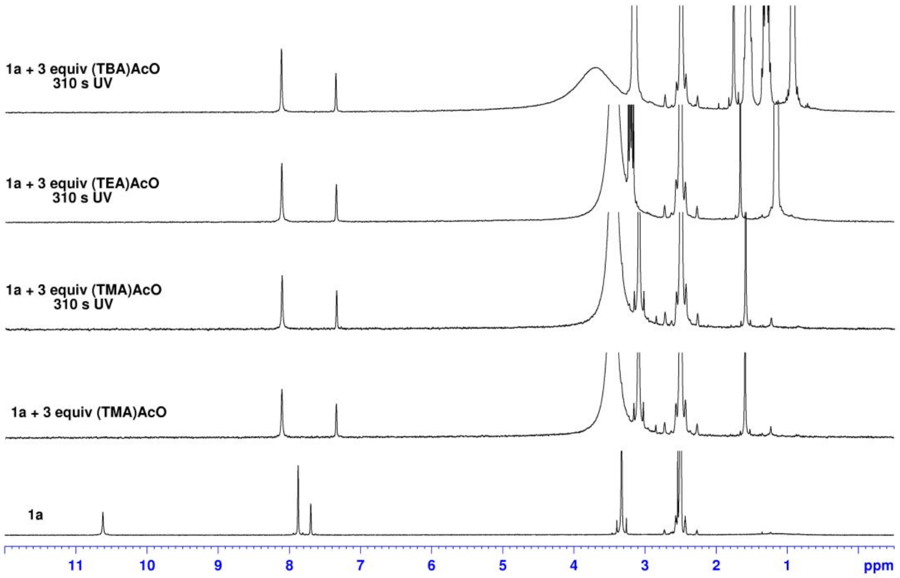

Figure S3.14. From bottom to top ^{1}H NMR (300 MHz) spectra of: **1a** [1.0 × 10^{-3} M] in DMSO-d_6., deprotonated **1a** obtained from adding to a **1a** [1.0 × 10^{-3} M] in DMSO-d_6 3 equivalents of (TMA) AcO; and 310 s UV irradiated samples of the mixture of **1a** [1.0 × 10^{-3} M] in DMSO-d_6 with 3 equivalents of (TMA)AcO, (TEA)AcO, and (TMA)AcO, respectively. The photoconversion of **1a** to **2a** is inhibited by the presence of the ion acetate which is basic enough for squaramide deprotonation. This behavior is not affected by the influence of the counter cation.

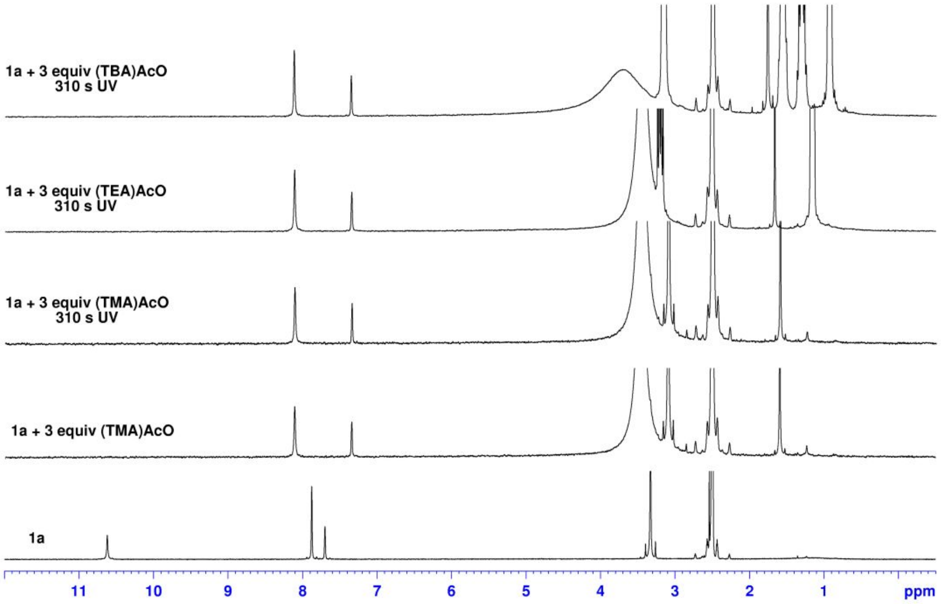

Figure S3.15 From bottom to top ^{1}H NMR (300 MHz) spectra of: **1a** [1.0 × 10^{-3} M] in DMSO-d_6, **2a** [1.0 × 10^{-3} M] in DMSO-d_6, and irradiated samples for 310 s of the mixture of **1a** [1.0 × 10^{-3} M] in DMSO-d_6 with 3 equivalents of (TMA)NO_3, (TEA)NO_3, and (TBA)NO_3. Nitrate is less basic than acetate and unable to deprotonate this squaramide **1a**. The clean photoconversion of **1a** to **2a** occurs unaffected by the counter cation changes.

4. Observation of dimethyl sulfide (^{1}H NMR) in a reaction sample

The formation of dimethyl sulfide was observed by recording the ^{1}H NMR spectra of an entire aliquot taken in a preparative scale phototoreaction.

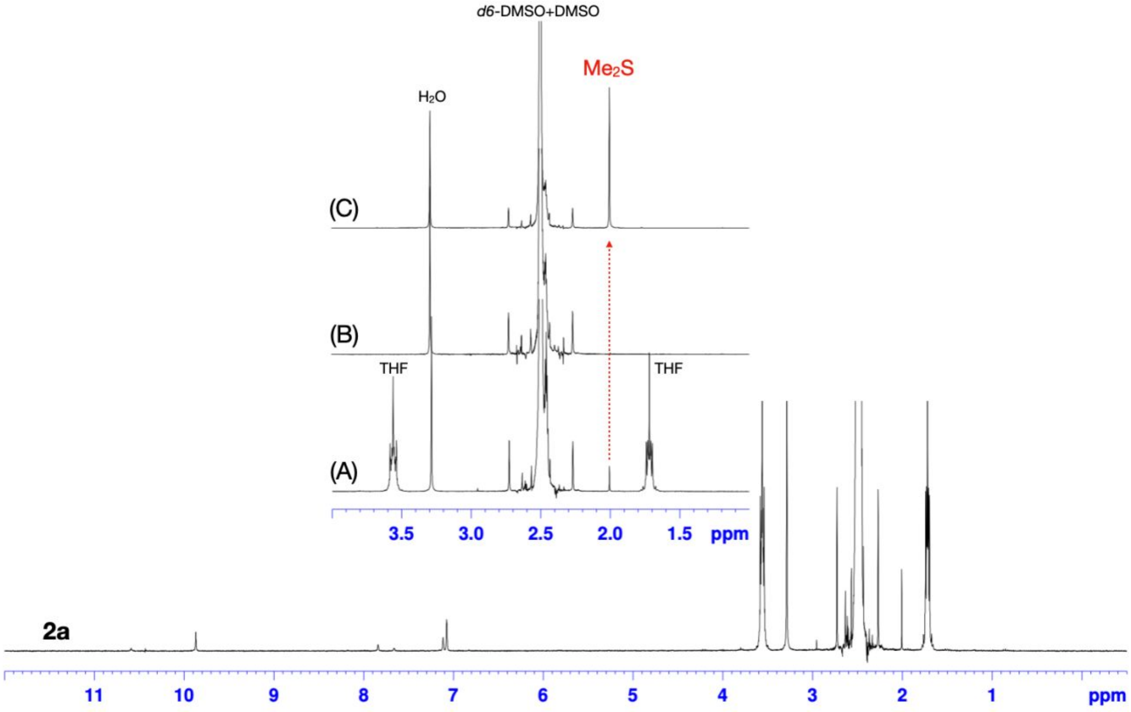

Figure. S4. Representative ^{1}H NMR (DMSO-d_6) whole spectrum showing the presence of Me_2S in a sample aliquot taken at the end of a preparative-scale photoreaction of AD-squaramide **1a** to **2a** in a 2:1 (v/v) mixture of THF-DMSO. The inset shows the aliphatic portion of the spectra. (A) Dimethyl sulfide appears as a sharp singlet at 2.0 ppm. (B) ^{1}H NMR spectrum of a sample of DMSO + DMSO-d_6. (C) ^{1}H NMR spectrum of the DMSO + DMSO-d_6 solvent + externally added authentic Me_2S for comparison.

5. Single-Crystal X-ray Diffraction

Single crystals of **1b**, **1c**, **1d**, **2a**, **2c,** and **3a** were selected, covered with Parabar 10320 (formally known as Paratone N), and mounted on a cryoloop on a D8 Venture diffractometer, with a Photon III 14 detector, using an Incoatec high brilliance IμS DIAMOND Cu tube equipped with an Incoatec Helios MX multilayer optics. The crystals were kept at room temperature during data collection. Data reduction and cell refinements were performed using the Bruker APEX4 program.[s3] Scaling and absorption correction was carried out using the SADABS program in all cases, except for **2c**, where TWINABS was used to produce an HKL4 file for the major component of the twin.[s3]

Using Olex2,[s4] the structures were solved with the XT structure solution program using intrinsic phasing and refined with the XL refinement package[s5] using least squares minimization. All non-hydrogen atoms were refined with anisotropic thermal parameters by full-matrix least-squares calculations on F^2. Hydrogen atoms were inserted at calculated positions and refined as riders, except for those from the water molecules in compound **1b** and those bound to N atoms in compound **1d** which were located using a Fourier difference map and refined isotropically. The DMSO molecule in **1c** has been split in two positions with 90 % and 10 % fractional occupancy. The DMSO molecule in **1d** has been split into two positions with 82.4 % and 17.6 % fractional occupancy. In compound **3a** the methoxy group has been split in two positions with 61.7 % and 38.3 %, while the trifluoromethyl moieties have been split in two positions of complementary occupancies: 71.9 % for F17-F19 and 28.1 % for F17A-F19A; 47.1 % for F21-F23 and F32-F34 and 52.9 % for F21A-F23A and F32A-F34A; 60.1 % for F36-F38 and 39.9 % for F36A and F38A.

The structures were checked for higher symmetry with the help of the program PLATON.[s6] The graphical materials have been prepared with the help of Mercury software.[s7]

A summary of the key crystallographic information is given in Table s1. CCDC 2242678-2242683 contains the supplementary crystallographic data.

5.1 Table S1. Crystal data and structure refinement for compounds 1b, 1c, 1d, 2a, 2c, 3a.

	**1b**	**1c**	**1d**	**2a**	**2c**	**3a**
CCDC	2242679	2242678	2242681	2242682	2242683	2242680
Empirical formula	$C_{20}H_{10}N_6O_3$	$C_{20}H_{16}N_4O_3S$	$C_{18}H_{20}N_4O_7S_3$	$C_{20}H_8F_{12}N_2O_3$	$C_{18}H_{10}N_4O_3$	$C_{23}H_{15}F_{12}N_3O_3$
Formula weight	382.34	392.43	500.56	552.28	330.30	609.38
Temperature (K)	303.0	302.00	301.00	290.00	297.00	298.00
Crystal system	monoclinic	monoclinic	triclinic	triclinic	triclinic	triclinic
Space group	$P2_1/c$	$P2_1/c$	P-1	P-1	P-1	P-1
a (Å)	10.6055(2)	16.4222(3)	10.2666(8)	8.991(2)	9.1282(9)	9.1390(3)
b (Å)	10.3128(2)	6.9568(2)	10.4673(9)	13.492(3)	11.8614(12)	12.2822(6)
c (Å)	16.1648(3)	18.0031(4)	11.3484(9)	17.790(4)	15.4657(16)	13.5379(7)
α (°)	90	90	111.963(3)	84.369(11)	70.257(4)	100.320(2)
β (°)	100.2090(10)	112.0430(10)	103.366(3)	83.215(10)	89.285(4)	98.468(2)
γ (°)	90	90	96.955(3)	89.251(10)	83.842(4)	105.172(2)
Volume ($Å^3$)	1739.99(6)	1906.44(8)	1070.89(15)	2132.4(9)	1566.5(3)	1326.7(1)
Z	4	4	2	4	4	2
ρ_{calc} (g/cm^3)	1.460	1.367	1.552	1.720	1.401	1.525
μ (mm^{-1})	0.863	1.759	3.616	1.695	0.824	1.430
F(000)	784.0	816.0	520.0	1096.0	680.0	612.0
Crystal size (mm^3)	0.25 × 0.12 × 0.08	0.28 × 0.25 × 0.12	0.16 × 0.11 × 0.08	0.2 × 0.03 × 0.03	0.15 × 0.12 × 0.08	0.11 × 0.10 × 0.08
2Θ range for data collection (°)	8.472 to 136.656	9.996 to 136.638	8.808 to 137.454	5.026 to 128.718	6.074 to 136.838	7.218 to 136.722
Index ranges	-12 ≤ h ≤ 12<br>-11 ≤ k ≤ 12<br>-19 ≤ l ≤ 19	-19 ≤ h ≤ 19<br>-8 ≤ k ≤ 8<br>-20 ≤ l ≤ 21	-12 ≤ h ≤ 12<br>-12 ≤ k ≤ 12<br>-13 ≤ l ≤ 13	-9 ≤ h ≤ 10<br>-15 ≤ k ≤ 13<br>-18 ≤ l ≤ 19	-10 ≤ h ≤ 10<br>-14 ≤ k ≤ 14<br>-18 ≤ l ≤ 18	-9 ≤ h ≤ 10<br>-14 ≤ k ≤ 14<br>-16 ≤ l ≤ 16
Reflections collected	20355	48527	39285	18726	9978	38192
Independent reflections	3154 [R_{int} = 0.0579, R_{sigma} = 0.0509]	3486 [R_{int} = 0.0527, R_{sigma} = 0.0360]	3818 [R_{int} = 0.0738, R_{sigma} = 0.0500]	5747 [R_{int} = 0.0577, R_{sigma} = 0.0530]	5560 [R_{int} = 0.0424, R_{sigma} = 0.0507]	4814 [R_{int} = 0.0512, R_{sigma} = 0.0396]
Data/restraints/parameters	3154/2/270	3486/0/264	3818/168/339	5747/0/668	5560/0/451	4814/15/505
Goodness-of-fit on F^2	1.436	1.045	1.218	1.093	1.100	1.069
Final R indexes [I>=2σ (I)]	R_1 = 0.1103<br>wR_2 = 0.3039	R_1 = 0.0408<br>wR_2 = 0.1147	R_1 = 0.0887<br>wR_2 = 0.2543	R_1 = 0.0909<br>wR_2 = 0.2564	R_1 = 0.0611<br>wR_2 = 0.1718	R_1 = 0.0492<br>wR_2 = 0.1413
Final R indexes [all data]	R_1 = 0.1143<br>wR_2 = 0.3111	R_1 = 0.0435<br>wR_2 = 0.1181	R_1 = 0.0933<br>wR_2 = 0.2640	R_1 = 0.1209<br>wR_2 = 0.2858	R_1 = 0.0648<br>wR_2 = 0.1739	R_1 = 0.0583<br>wR_2 = 0.1506
Largest diff. peak/hole (e $Å^{-3}$)	0.84/-0.52	0.21/-0.19	0.88/-0.72	0.90/-0.58	0.34/-0.26	0.16/-0.14

5.2. ORTEP (50%) Representations

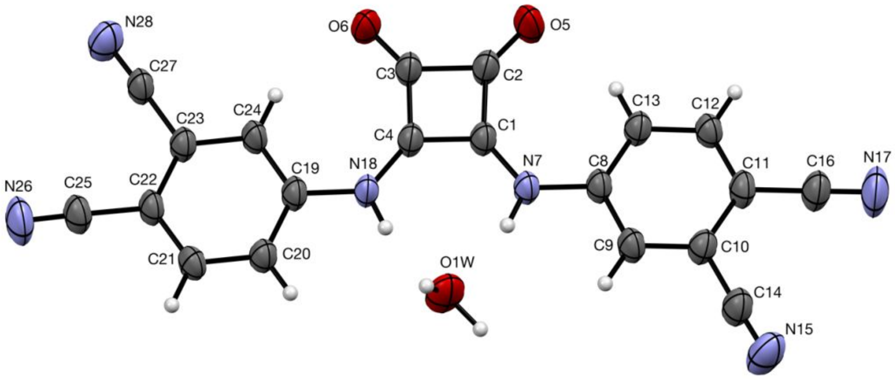

Figure S5.1. ORTEP representations (50 % probability) for the asymmetric units of AD-squaramide **1b**. Crystals were obtained from a wet MeCN solution. Two partial occupancy positions have been solved; the highest fraction is depicted. In this structure, the (a,a) conformation is particularly favoured due to CHAr···OSq (3.07-3.11Å; θ_{CHO} 155.4°) intramolecular hydrogen bonds. The angle between the planes formed by the squaramide and the aromatic rings is < 5°, giving rise to almost planar structures with the aryl rings fully conjugated to the squaramide moiety.

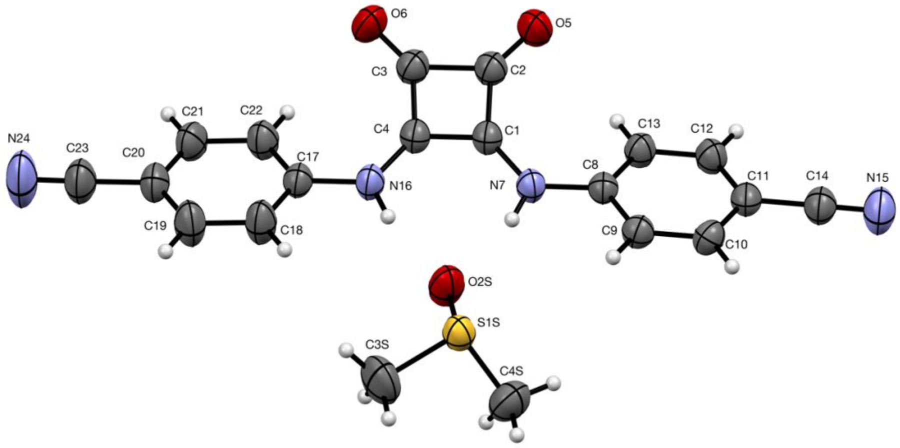

Figure S5.2. ORTEP representations (50 % probability) for the asymmetric units of AD-squaramide **1c**. Two partial occupancy positions have been solved (DMSO: 90 and 10%), the highest fraction is depicted. Crystals were obtained from a DMSO solution.

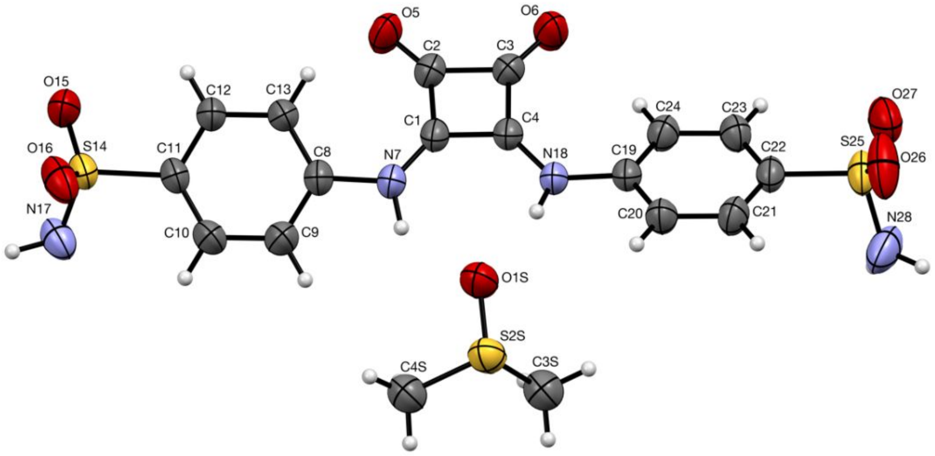

Figure S5.3. ORTEP representations (50 % probability) for the asymmetric units of AD-squaramide **1d**. Two partial occupancy positions have been solved (DMSO: 82.4, 17.6 %), the highest fraction is depicted. Crystals were obtained from a DMSO solution.

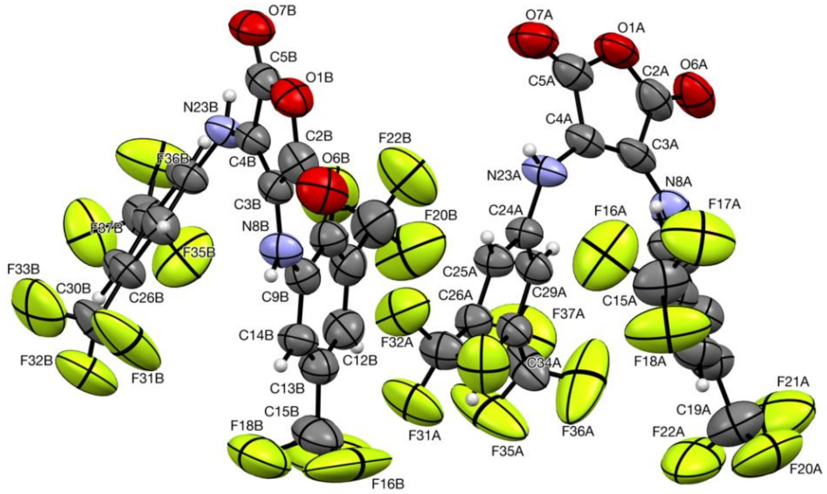

Figure S5.4. ORTEP representations (50 % probability) for the asymmetric units of AD-squaramide **2a**. Crystals were obtained by slow evaporation of a wet MeCN solution. The intramolecular distance between aryl centroids is 3.967Å, well within the range of arene stacking interactions.

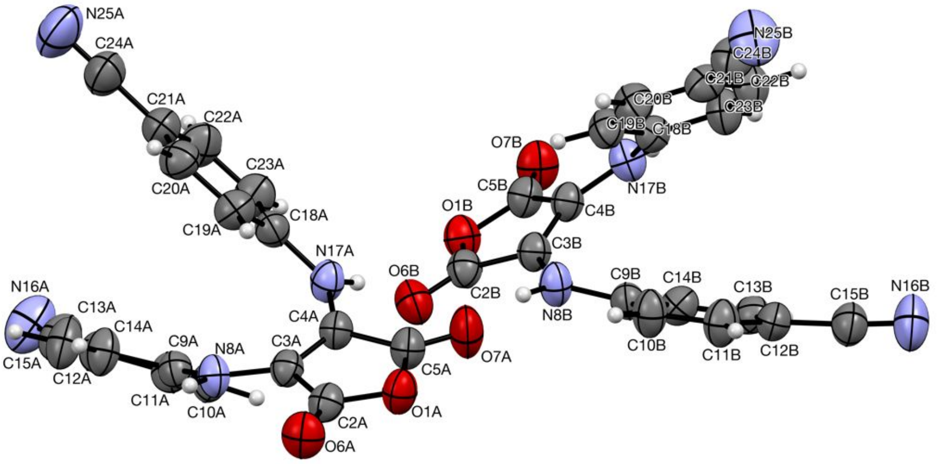

Figure S5.5. ORTEP representations (50 % probability) for the asymmetric units of AD-squaramide **2c**. Crystals were obtained by slow evaporation of a wet MeCN solution. The intramolecular distance between aryl centroids is 3.811Å, well within the range of arene stacking interactions.

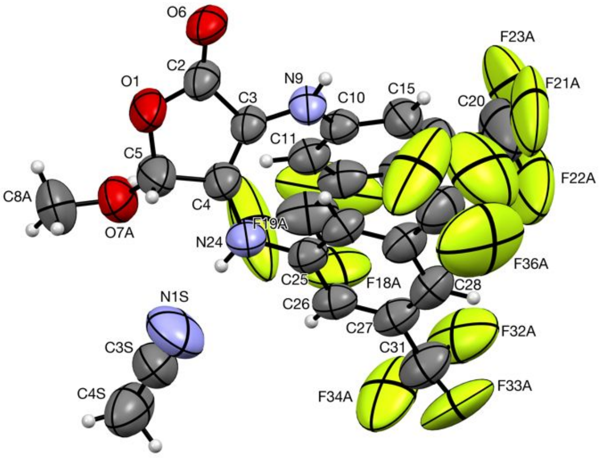

Figure S5.6. ORTEP representations (50 % probability) for the asymmetric units of AD-squaramide **3a**. Two partial occupancy positions have been solved (MeO: 61.7 and 38.3%), the highest fraction is depicted. Crystals were obtained by slow evaporation of a wet MeCN solution.

6 Theoretical calculations

Computational calculations were carried out using Spartan'18 software, version 1.4.4 (Wavefunction)

To evaluate the conformational preferences of the anhydrides, we performed DFT calculations (W97X-D/6-31G*). First, the initial geometries of **2a** conformers (a,a) and (s,s) were energy-minimized with a molecular mechanics force-field. Then, the geometries of the two conformers were optimized using DFT (W97X-D/6-31G*) level of theory. Both conformers lead to the energy minimum depicted in **Figure S6.1**, an (s,s)-**2a** conformer akin to that observed in the solid-state. The (a,a)-**2a** conformation is neither an energy minimum nor a saddle point (See, cartesian coordinates and frequencies lists in ANNEX I)

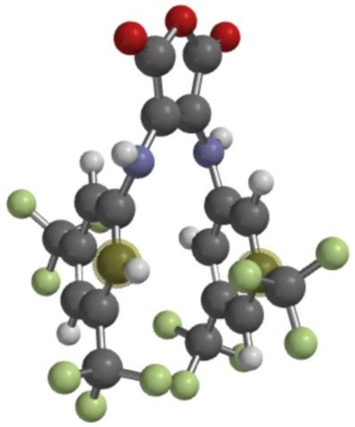

**Job type:**	Equilibrium Geometry
**Method:**	ωB97X-D
**Basis set:**	6-31G*
**With Options:**	PRINTCOORDS
**Energy:**	-2299.632459 hartrees

Figure S6.1. Energy-minimized structure of anhydride **2a**. See ANNEX I.

2a-(a,a)conformer

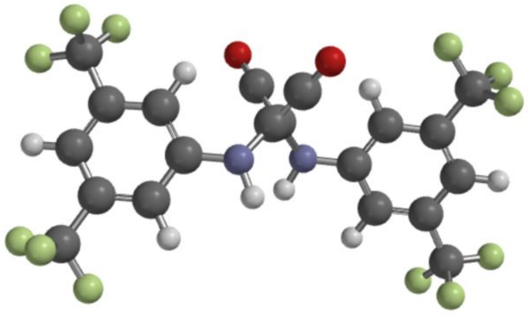

**Job type:**	Equilibrium Geometry
**Method:**	ωB97X-D
**Basis set:**	6-31G*
**With Options:**	PRINTCOORDS
**Energy:**	-2224.315918 hartrees

2a-(s,s) conformer

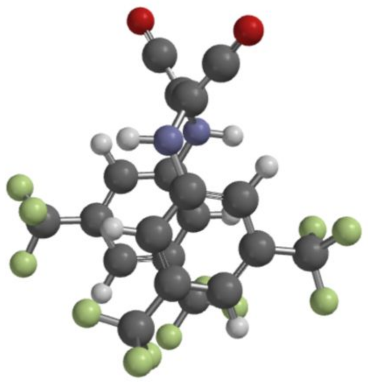

**Job type:**	Equilibrium Geometry
**Method:**	ωB97X-D
**Basis set:**	6-31G*
**With Options:**	PRINTCOORDS
**Energy:**	-2224.331581 hartrees

Figure S6.2. Relative energy difference between the two bis-ketene intermediates **2a** in (a,a) and (s,s) conformation, respectively. See ANNEX I.

Energy-minimized structures of the bisketene **1a** in (a,a)-type, and (s,s)-type conformations. The energy difference is 40 kjmol^{-1} in favour of conformer (s,s).

7. ^{1}H NMR titrations of 1a and 2a with $N(nBu)_4Cl$

Samples of host of known concentration (aprox. 4.0 $\times10^{-3}$ M) in DMSO-d_6- 0.5% H_2O or MeCN-d_3-0.5% H_2O were prepared. 500 µL of this solution was removed, and a ^{1}H NMR spectrum was recorded. Then, an accurately weighted sample of guest $N(nBu)_4Cl$ (5.0 $\times10^{-2}$ M), was dissolved in the remaining 1.5 mL of host solution. Appropiate aliquots of titrant guest were added to the NMR tube containing the initial host solution (titration at constant concentration of host). After each addition the solution was shaken and left to stand for 2 min before recording the spectra. For signals that moved more than 0.01 ppm, the chemical shifts of the NH and aromatic protons at all concentrations of guest were recorded and analysed using HypNMR2008.[s9]

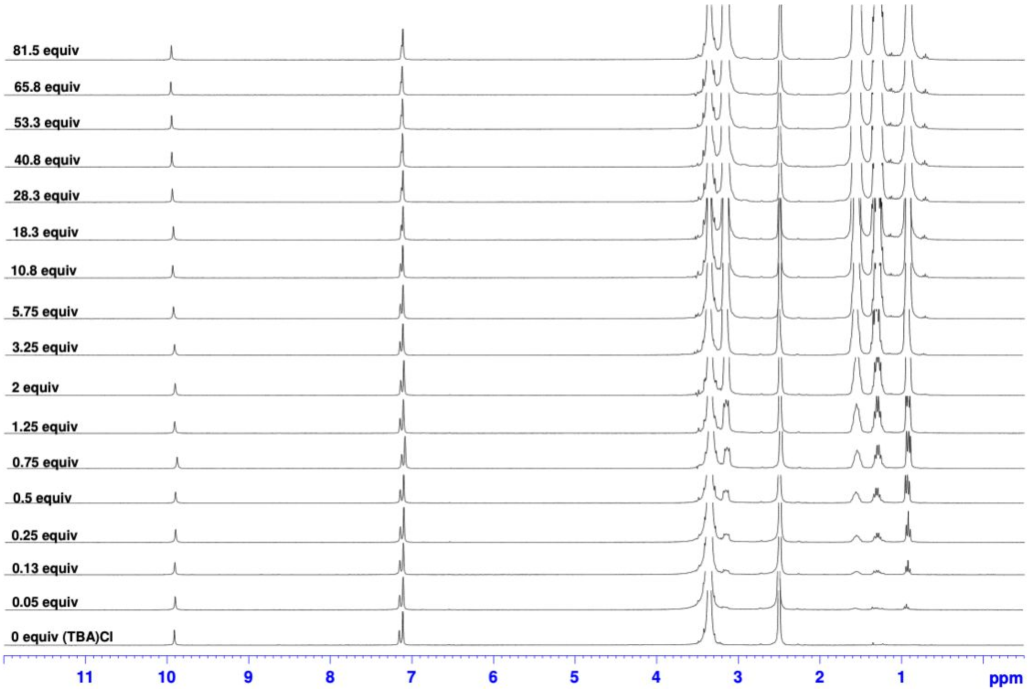

Figure S7.1. 1H NMR stacked plots representative of the titration of anhydride **2a** (4.0×10^{-3}M) in DMSO-d_6-0.5% H_2O with $N(nBu)_4Cl$ (5.0×10^{-2}M). After addition of > 80 equivalents of TBACl the NH and the aromatic protons move downfield and upfield, respectively, less than 0.05 ppm. The affinity constant is < 5M^{-1}.

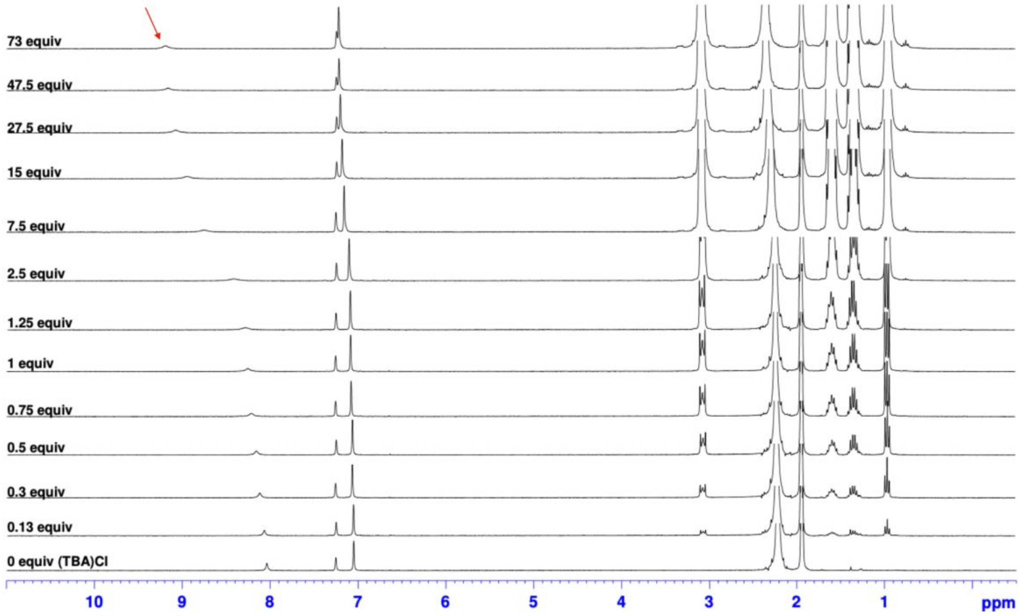

Figure S7.2. 1H NMR stacked plots representative of the titration of anhydride **2a** (4.0×10^{-3}M) in MeCN-d_6-0.5% H_2O with $N(nBu)_4Cl$ (5.0×10^{-2}M). The affinity constant derived from these data by non-linear curve-fitting was 28M^{-1}.

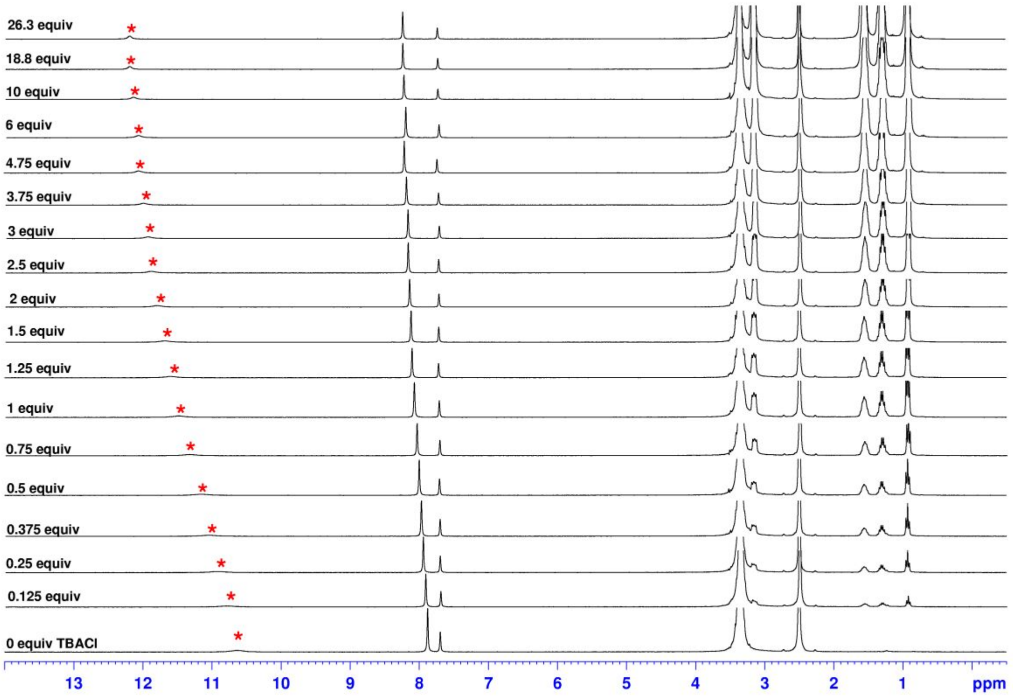

Figure S7.3. ^{1}H NMR stacked plots representative of the titration of squaramide **1a** (4.0×10^{-3}M) in DMSO-d_6-0.5% H_2O with $N(nBu)_4Cl$ (1.0×10^{-2}M) The affinity constant derived from these data by non-linear curve-fitting was 619 M^{-1}.

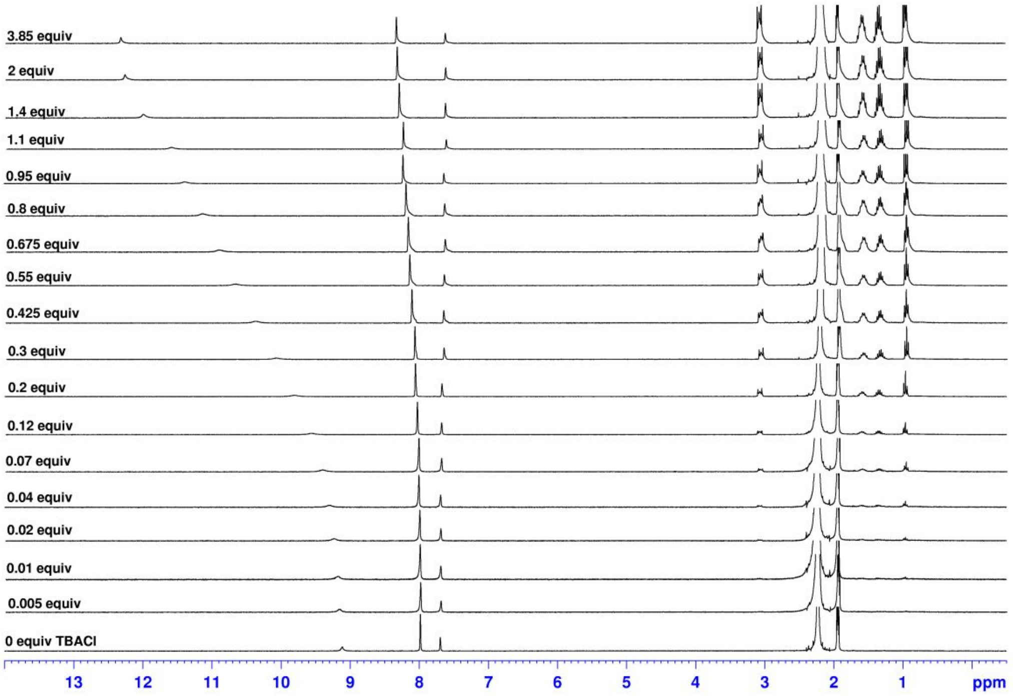

Figure S7.4. ^{1}H NMR stacked plots representative of the titration of anhydride **1a** (4.0×10^{-3}M) in MeCN-d_6-0.5% H_2O with $N(nBu)_4Cl$ (5.0×10^{-2}M). The affinity constant derived from these data by non-linear curve-fitting was > 10^4 M^{-1}.

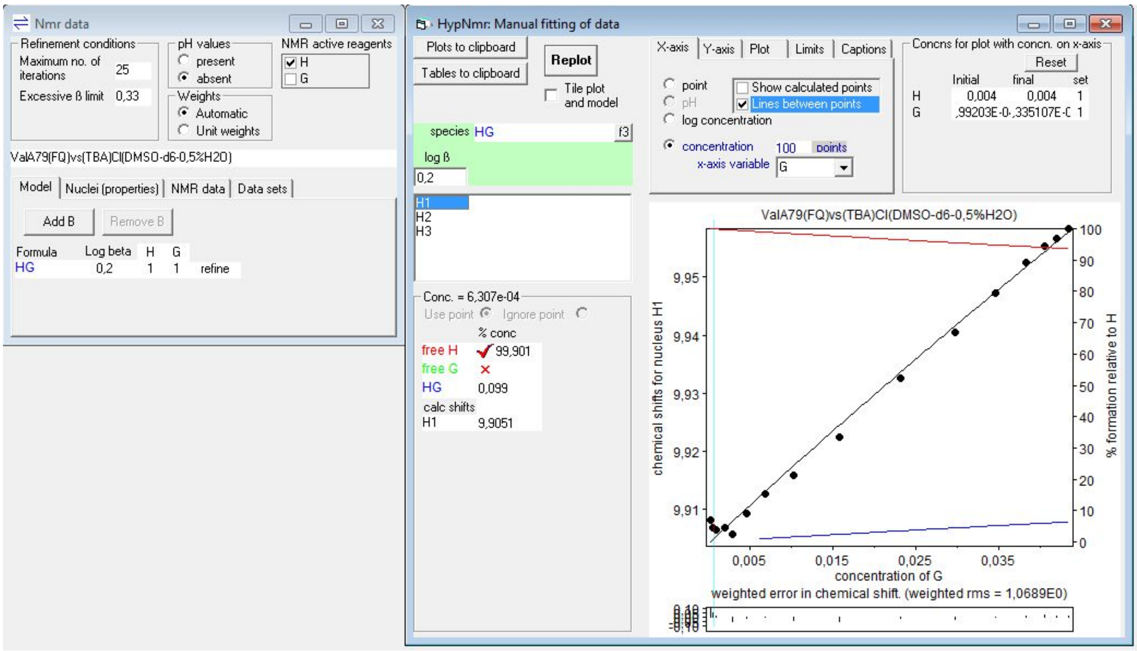

Figure S7.5. Data fitting to the 1:1 binding model of the^{1}H NMR (300 MHz) titration of anhydride **2a** (4.0×10^{-3}M) in DMSO-d_6-0.5% H_2O with $N(nBu)_4Cl$ ($5.0\ 10^{-2}$M). From these data, K_a < 5M^{-1}.

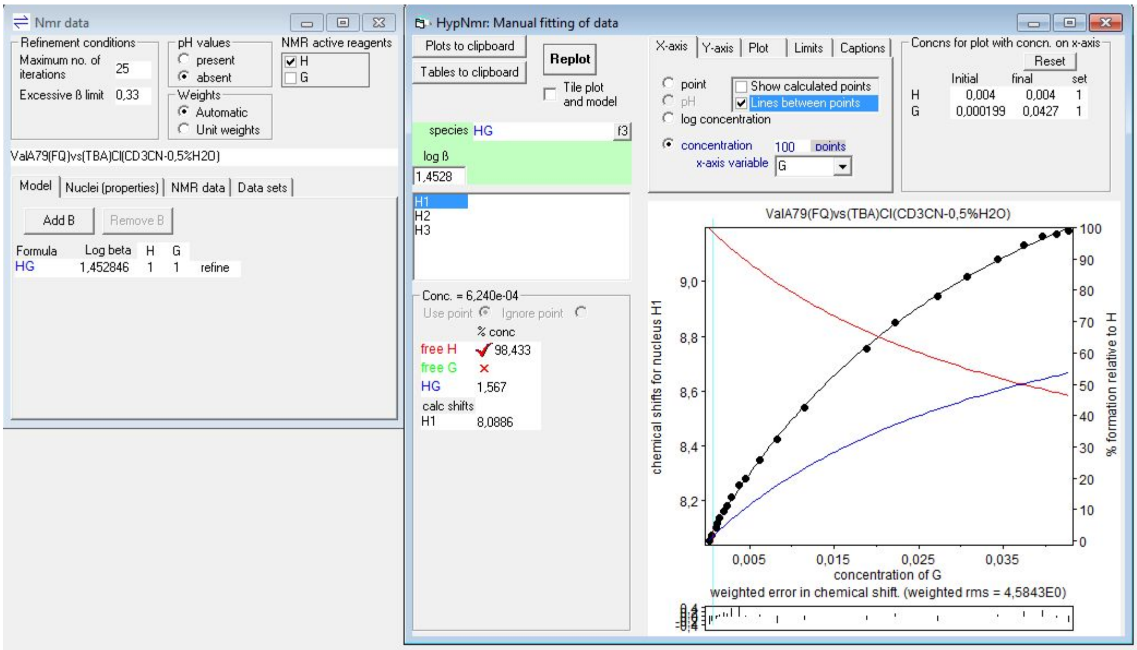

Figure S7.6. Data fitting to the 1:1 binding model of the^{1}H NMR (300 MHz) titration of anhydride **2a** (4.0×10^{-3}M) in CD_3CN-0.5% H_2O with $N(nBu)_4Cl$ (5.0×10^{-2}M). From these data, K_a = 28M^{-1}

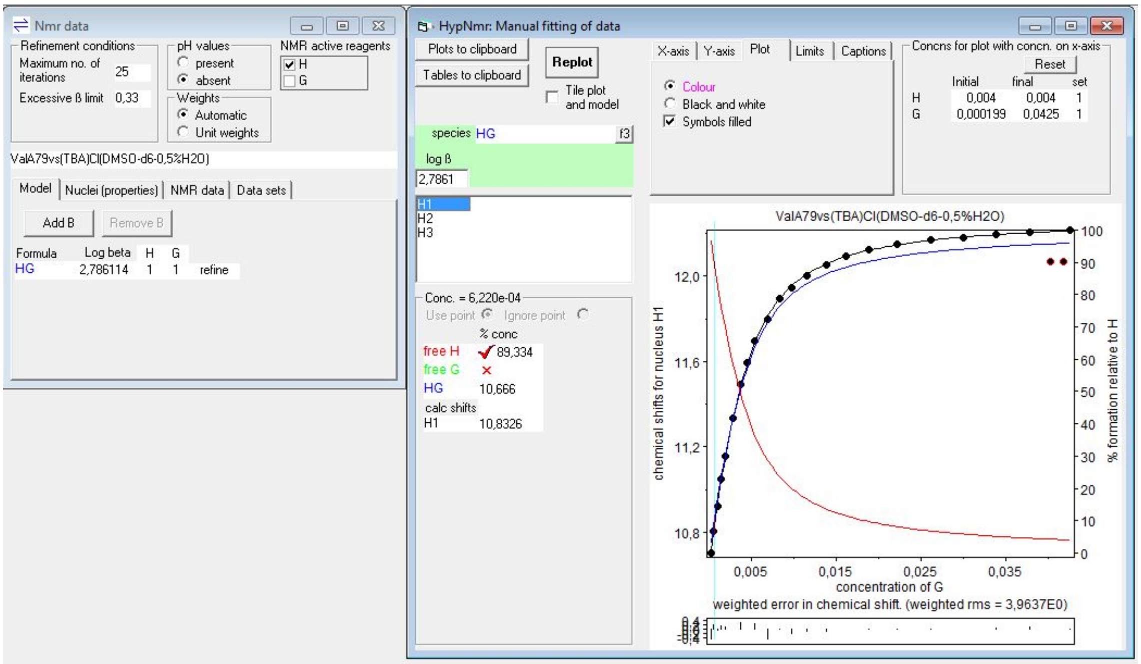

Figure S7.7. Data fitting to the 1:1 binding model of the^{1}H NMR (300 MHz) titration of squaramide **1a** (4.0×10^{-3}M) in DMSO-d_6-0.5% H_2O with $N(nBu)_4Cl$ (5.0×10^{-2}M). From these data, K_a = 611M^{-1}

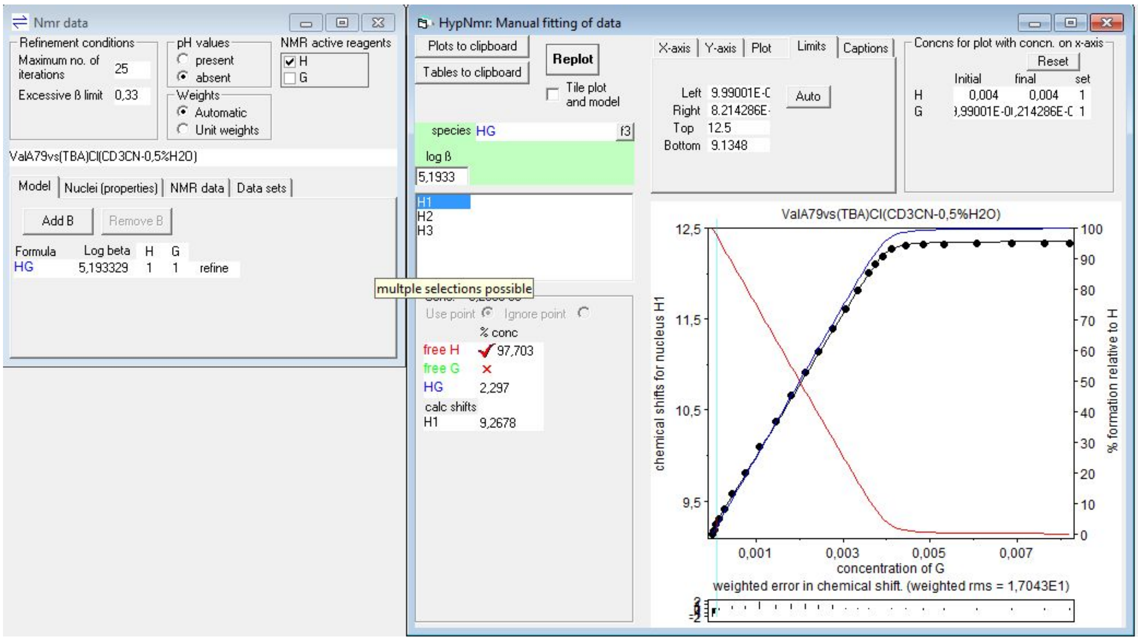

Figure S7.8. Data fitting to a 1:1 binding model of the ^{1}H NMR (300 MHz) titration of squaramide **1a** (4.0×10^{-3}M) in CD_3CN-0.5% H_2O with $N(nBu)_4Cl$ (5.0×10^{-2}M). From these data, K_a = 1.56×10^{5} M^{-1} (>$10^{4}M^{-1}$)

8 Transport experiments

8.1 Preparation of the vesicles

1-palmitoyl-2-oleoyl-sn-glycero-3-phosphocholine (POPC) and cholesterol solutions (15-30 mM) in deacidified chloroform were combined in a 5 mL round bottom flask. The volumes of the aliquots were calculated from the concentrations of the lipid solutions to obtain a POPC to cholesterol ratio of 7:3 (for instance by combining 7 µmol POPC and 3 µmol cholesterol). The solvents were evaporated under a flow of nitrogen and the resulting lipid film was dried under high vacuum for at least 1 h.

The lipid film was then hydrated with 750 µL of an aqueous solution of N,N′-Dimethyl-9,9′-biacridinium dinitrate (Lucigenin, 0.8 mM) in a solution of $NaNO_3$ (225 mM). The resulting mixture was sonicated for 30 s and stirred for 1 h to give heterogeneous vesicles. Multilamellar vesicles were disrupted by 10 freeze-thaw cycles. The mixture was diluted to 1 mL (by adding 0.5 mL of $NaNO_3$ solution) and extruded 29 times through a polycarbonate membrane with 200 nm pores in a mini-extruder (Avanti Polar Lipids). The external dye was removed by passing the liposomes through a pre-packed size exclusion column (containing 8.3 mL Sephadex G-25 medium), eluted with $NaNO_3$ solution. The collected large unilamellar vesicles (LUVs) were further diluted with $NaNO_3$ solution to obtain total lipid concentration of 0.4 mM (50 mL) and used for transport measurements the same day.

			Size (d.nm):	% Intensity:	St Dev (d.nm):
Z-Average (d.nm):	160.4	Peak 1:	177.4	98.8	66.84
PdI:	0.162	Peak 2:	4999	1.2	608.4
Intercept:	0.967	Peak 3:	0.000	0.0	0.000
Result quality :	Good				

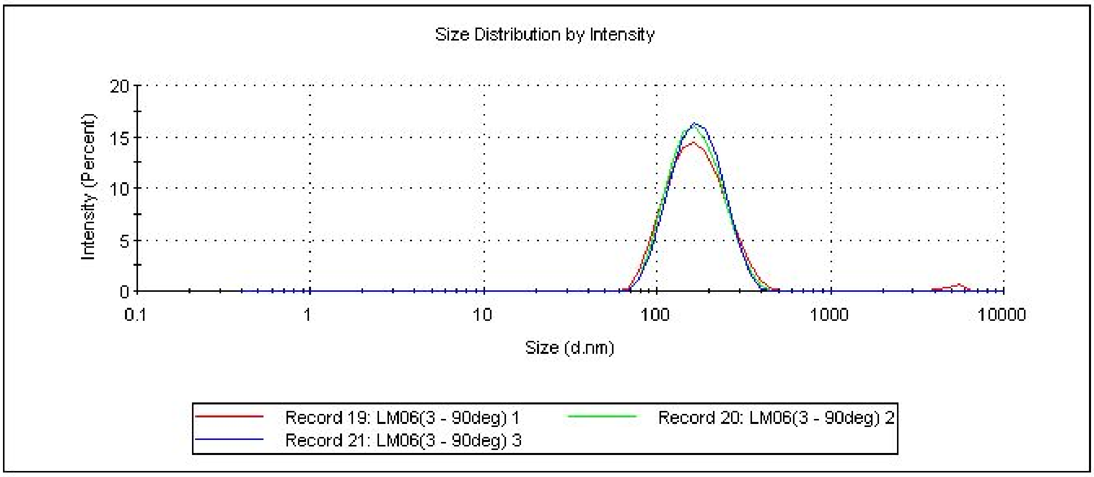

Figure S8.1 DLS (Malvern, ZS90) data of LUVs with lucigenin encapsulated and suspended in 225 mM $NaNO_3$ for three consecutive measurements (using a refractive index of 1.45 for the liposomes and a refractive index of 1.33 and viscosity of 0.88 mPa·s for the aqueous solution). These curves are indicative of a monodisperse suspension of vesicles with a mean diameter (z-average) of 160 nm

8.2 Chloride transport measurements

8.2.1 General measurements (irradiation in situ)

3.00 mL of the freshly prepared liposomes solution (0.4 mM lipids) were placed in a quartz cuvette with a small stir bar, and the cuvette was placed inside the sample compartment of a Varian Cary Eclipse Fluorescence Spectrometer. The transporter was added to the liposomes as 12 µL of a stock solution (0.1-0.0001 mM) in methanol. These additions were performed using a Hammilton syringe and placing the end of the tip close to the magnetic bar on the bottom of the cuvette, while stirring. For blank curves, 12 µL of methanol were added. The sample was irradiated for 0, 2, 5 or 8 seconds (365 nm, 10 W), by coupling a small LED-UV flashlight lamp right to the top of the cuvette. The temperature was allowed to stabilize by stirring at 25 ˚C for 3-5 minutes (these conditions were maintained during the whole transport experiment), and after equilibration the transport measurement was started. During transport

measurements the fluorescence intensity at 505 nm (20 nm slits) was monitored over time (15 minutes, 0.2 s interval) with excitation at 430 nm (10 nm slits).

For transport measurements 75 µL of NaCl (1 M in H_2O) were added to the liposomes 30 s after the start of the fluorescence recording, to create a Cl^- concentration gradient of 25 mM, and the fluorescence intensity was measured for another 10 minutes.

Each experiment was performed at least twice, and the data of the different runs were averaged and normalized. To normalize the curves, the initial 30-40 seconds were deleted to get rid of the fluorescence intensity in absence of NaCl (first 30 s) and the initial drop in fluorescence intensity after addition of NaCl (provoked by extravesicular lucigenin), and all the values were divided by the initial value of the resulting curve (F/F_0). The transport curves show the first 500 seconds of transport.

8.2.2 Effect of photodegradation on the transport activity

To prove that the photodegradation of squaramide **1a** involves a loss of chloride transport activity, we first recorded transport curves before and after irradiation (medium pressure Hg lamp, 400 W, 10 min) of a stock solution of **1a** (0.1 mM in methanol; 12 µL for a 1:1k transporter:lipid ratio) (Figure S8.2). The irradiation in methanol decreased the activity of **1a** around 100 times.

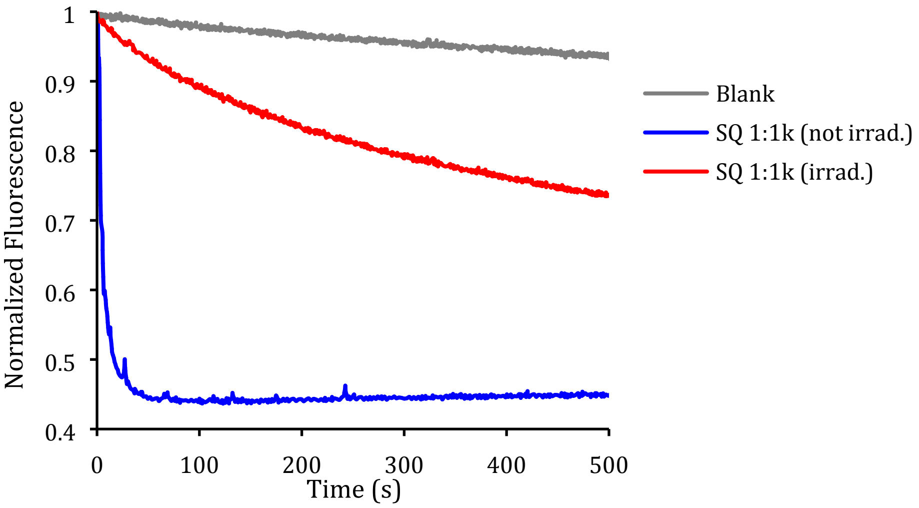

Figure S8.2. Chloride transport by squaramide **1a** (at 1:1k transporter:lipid ratio) before (blue) and after (red) irradiation of a stock solution of the squaramide in MeOH (0.1 mM). LUVs were formed of POPC:cholesterol (70:30) and suspended in 225 mM $NaNO_3$ solution (interior and exterior). Transport was monitored by the quenching of the fluorescence of encapsulated lucigenin (0.8 mM) upon addition of 25 mM NaCl. The blank curve was recorded in absence of transporter.

8.2.3. Functionality of the Lucigenin⊏LUVs after irradiation

To assess the effect of irradiation on the transport data obtained from the vesicles, we performed transport studies by the procedure described above, but adding the transporter after the irradiation step. This proved that the irradiation of the Lucigenin⊏LUVs has little effect on the transport curve registered with the active transporter (Figure S8.3).

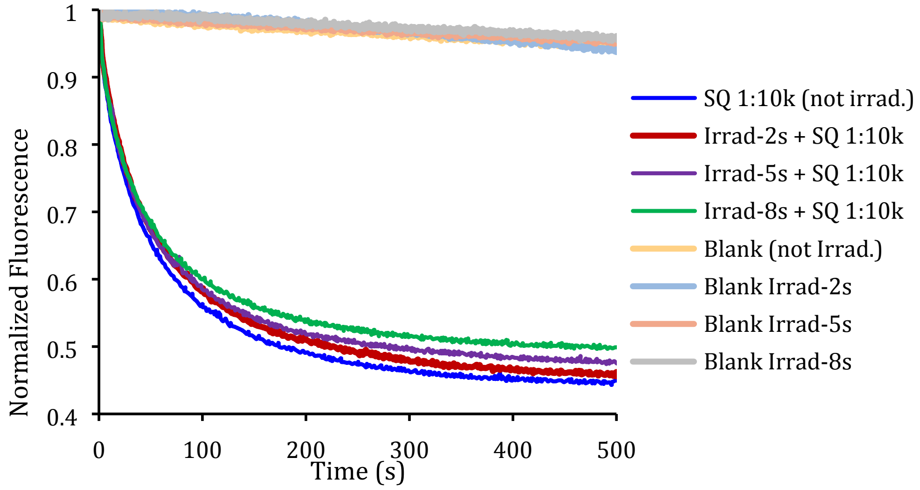

Figure S8.3. Chloride transport by squaramide **1a** (at 1:10k transporter:lipid ratio) into LUVs irradiated for 0, 2, 5 and 8 seconds. The transporter was added after the irradiation step to avoid its photodegradation. LUVs were formed of POPC:cholesterol (70:30) and suspended in 225 mM $NaNO_3$ solution (interior and exterior). Transport was monitored by the quenching of the fluorescence of encapsulated lucigenin (0.8 mM) upon addition of 25 mM NaCl. The blank curves were recorded in absence of transporter. The subtle differences observed for the normalized transport curves might be a consequence of partial photo-bleaching of lucigenin.

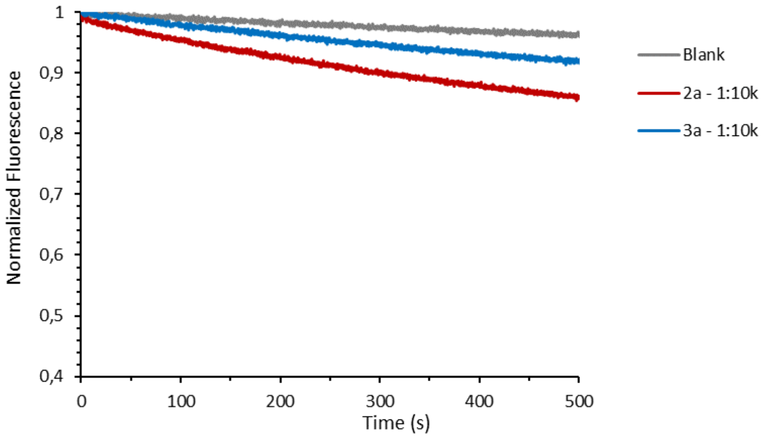

Figure S8.4. Chloride transport by compounds **2a** (red line) and **3a** (blue line) (at 1:10k transporter:lipid ratio) into LUVs. LUVs were formed of POPC:cholesterol (70:30) and suspended in 225 mM $NaNO_3$ solution (interior and exterior). Transport was monitored by the quenching of the fluorescence of encapsulated lucigenin (0.8 mM) upon addition of 25 mM NaCl. The blank curve was recorded in absence of transporter.

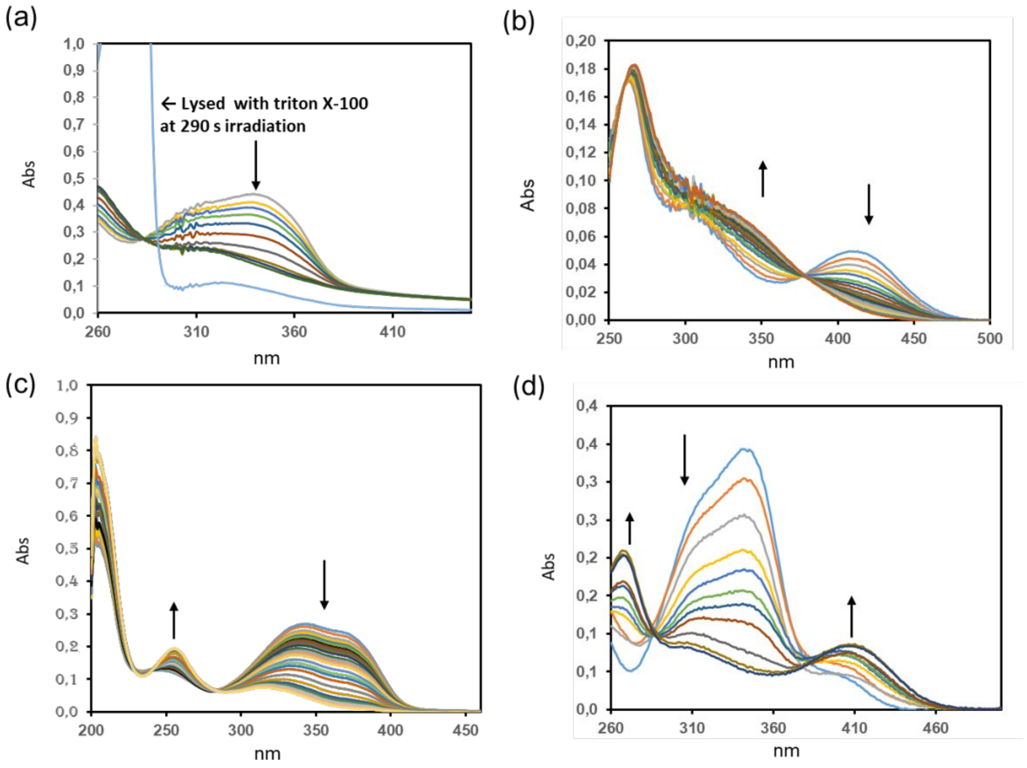

Figure S8.5. UV studies (a) photoconversion of **1a** in LUVs 0.2 mM (10% DMSO) at 1:10k transporter:lipid ratio) into LUVs irradiated with UV lamp (365nm, 10 W) for 0 to 290 s. LUVs were formed of POPC:cholesterol (70:30) and suspended in 225 mM $NaNO_3$ solution (interior and exterior) After 290 s of irradiation LUVs were lysed with triton X-100. (b) Changes registered in the UV spectra of **2a** (1.0×10^{-5} M) in H_2O ($NaNO_3$ 225 mM-1% DMSO) with time 0-200 s. (c) Changes registered for the UV spectrum of **1a** (1.0×10^{-5} M) in MeOH upon irradiation with a UV lamp (365nm, 10 W) for 0-120 s. (d) Changes registered for the UV spectrum of **1a** (1.0×10^{-3} M) in DMSO upon irradiation with a UV lamp (365nm, 10 W) for 0-20 s. UV spectra were recorded with 0.01 mm optical pass.

UV experiments in **Figure S8.5** show the photoconversion of **1a** in different solvents and in LUVs as we have seen by ^{1}H NMR. All the experiments show similar behaviour. The absorption band(s) of **1a** in the visible region decreases in intensity during the transformation and a new band of higher energy and lower intensity appears in the final state. The band observed in Figure S8.5a after the LUVs lysis agrees with the band observed as consequence of the hydrolysis of anhydride **2a** in water (Figure S8.5b).

9. References

s1 A. Rostami, A. Colin, X. Y. Li, M. G. Chudzinski, A. J. Lough and M. S. Taylor, *J. Org. Chem.*, 2010, **75**, 3983–3992.

s2 L. Pfeifer, K. M. Engle, G. W. Pidgeon, H. A. Sparkes, A. L. Thompson, J. M. Brown, V. Gouverneur, *J. Am. Chem. Soc.,* 2016, **138**, 13314-13325.

s3 APEX3, SAINT, SADABS and XP, Bruker AXS Inc., Madison, Wisconsin, USA, 2015.

s4 O. V. Dolomanov, L. J. Bourhis, R. J. Gildea, J. A. K. Howard and H. Puschmann, J. Appl. Crystallogr., 2009, **42**, 339–341.

s5 G. M. Sheldrick, *Acta Crystallogr., Sect. C: Struct. Chem*., 2015, **71**, 3–8.

s6 A. L. Spek, *Acta Crystallogr., Sect. D: Biol. Crystallogr*., 2009, **65**, 148–155.

s7 C. F. Macrae, I. J. Bruno, J. A. Chisholm, P. R. Edgington, P. McCabe, E. Pidcock, L. Rodriguez-Monge, R. Taylor, J. van de Streek and P. A. Wood, *J. Appl. Crystallogr*., 2008, **41**, 466470.

s8 Y. Shao, L.F. Molnar, Y. Jung, J. Kussmann, C. Ochsenfeld, S.T. Brown, *Phys. Chem. Chem. Phys*., 2006, **8**, 3172.

s9 HypNMR 2008 program (Protonic Software; http ://www.hyperquad.co.uk). (a) C. Frassineti, S. Ghelli, P. Gans, A. Sabatini, M. S. Moruzzi, A. Vacca, *Anal. Biochem*. 1995, ***231***, 374-382, (b) C. Frassineti, L. Alderighi, P. Gans, A. Sabatini, A. Vacca, S. Ghelli, *Anal. Bioanal. Chem*. 2003, ***376***, 1041-1052.

ANNEX I

Computational parameters and cartesian coordinates of anhydride **2a**

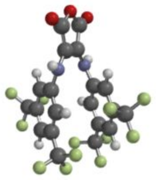

```
SPARTAN '18 Quantum Mechanics Program:  (x86/Darwin)        build  1.4.4

Job type: Geometry optimization.
Method: RWB97X-D
Basis set: 6-31G(D)
Number of basis functions: 571
Number of electrons: 274

SCF model:
 A restricted hybrid HF-DFT SCF calculation will be
 performed using Pulay DIIS + Geometric Direct Minimization


Optimization:
         Step      Energy          Max Grad.       Max Dist.
            1    -2299.632458       0.000411        0.004003
            2    -2299.632459       0.000258        0.007527

<step 2>
Job type: Frequency calculation.
Method: RWB97X-D
Basis set: 6-31G(D)

  Reason for exit: Successful completion
  Quantum Calculation CPU Time :   5:51:48.06
  Quantum Calculation Wall Time:   3:20:06.42


SPARTAN '18 Properties Program:  (x86/Darwin)                  build  1.4.4
  Use of molecular symmetry disabled
```

Cartesian Coordinates (Angstroms)

Atom			X	Y	Z
1	C	C11	0.9511091	0.3551167	1.3398158
2	C	C12	2.3587274	-1.0472450	-0.6316289
3	C	C13	1.6208398	1.0460974	0.3304002
4	C	C14	1.0178021	-1.0366655	1.3824199
5	C	C15	1.7048337	-1.7244848	0.3884357
6	C	C16	2.3217569	0.3434818	-0.6398791
7	C	C17	-1.7576602	-0.2734706	-0.0204176
8	C	C18	-0.7569092	0.8912801	-2.3530202
9	C	C19	-1.2590447	-1.0793553	-1.0451417
10	C	C20	-1.7898765	1.1085313	-0.1785715
11	C	C21	-1.2781538	1.6800759	-1.3402899
12	C	C22	-0.7584472	-0.4935821	-2.1960033
13	H	H31	1.5334442	2.1254597	0.2707441
14	H	H32	0.5129244	-1.5807549	2.1731739
15	H	H34	-1.1844312	-2.1496540	-0.8944328
16	H	H35	-2.1840934	1.7371811	0.6123705
17	N	N1	-2.1394132	-0.8678482	1.2006425
18	H	H3	-2.8257100	-1.6110615	1.1882271
19	N	N2	0.1683922	1.0739742	2.2648508
20	H	H6	0.5976265	1.8539780	2.7454085
21	C	C1	-0.9468144	0.5507891	2.8658860
22	C	C23	-1.9322052	-0.2637064	2.4090320
23	H	H10	2.8711149	-1.5893938	-1.4155157
24	C	C7	1.6759494	-3.2290173	0.4221151
25	C	C8	3.0129216	1.1265385	-1.7259468
26	F	F1	2.1302945	-3.7003647	1.5937923
27	F	F2	2.4085260	-3.7759650	-0.5530059
28	F	F3	0.4139196	-3.6895294	0.2868144
29	F	F4	2.1708812	2.0059946	-2.3012995
30	F	F5	4.0361515	1.8396281	-1.2290644
31	F	F6	3.4975843	0.3375629	-2.6909038
32	H	H9	-0.3247399	1.3436010	-3.2362416

Calculated frequencies of anhydride **2a**

	Uncorrected cm^{-1}	Corrected	Intensity
1	18	17	0.01
2	20	18	0.07
3	24	23	0.03
4	32	30	2.91
5	35	33	0.79
6	37	35	0.27
7	45	43	2.19
8	47	45	0.06
9	49	46	0.17
10	74	70	0.12
11	83	78	0.00
12	104	98	0.82
13	115	108	1.22
14	119	112	0.82
15	122	115	0.08
16	143	135	0.82
17	161	152	0.51
18	168	158	1.55
19	179	168	1.13
20	197	185	0.59
21	200	188	2.95
22	206	194	1.67
23	247	233	5.10
24	266	250	1.23
25	278	262	2.01
26	286	269	5.66
27	286	270	2.93
28	296	279	0.49
29	320	302	1.36
30	326	307	8.32
31	335	315	29.04
32	356	335	1.48
33	360	339	7.76
34	363	342	43.54
35	368	347	17.27
36	389	367	16.88
37	400	377	22.49
38	407	383	83.19
39	412	388	21.87
40	448	422	2.93
41	464	437	2.64
42	473	446	6.07
43	506	477	0.28
44	507	478	0.29
45	510	480	0.44
46	521	491	10.98
47	532	501	3.42
48	566	533	3.14
49	573	540	0.44
50	577	543	0.21
51	599	564	6.29
52	600	565	6.94
53	626	590	1.69
54	630	593	1.58
55	650	612	1.11
56	657	619	4.03
57	679	639	5.16
58	686	646	4.02
59	690	650	4.77
60	692	652	43.64
61	693	653	22.77
62	718	676	0.11
63	724	682	24.91
64	729	687	17.70
65	735	693	6.46
66	742	699	30.74
67	748	705	14.97
68	789	744	3.89

69	793	747	0.79
70	874	823	22.04
71	878	827	6.57
72	904	851	54.02
73	913	860	32.97
74	932	878	52.17
75	935	881	0.74
76	942	888	0.16
77	960	904	3.77
78	972	916	57.31
79	982	925	48.98
80	1013	955	24.23
81	1027	968	0.90
82	1028	968	1.23
83	1133	1067	4.55
84	1136	1070	19.13
85	1144	1077	14.23
86	1147	1080	4.78
87	1159	1092	104.54
88	1167	1100	7.91
89	1207	1137	16.17
90	1210	1140	375.96
91	1220	1150	391.71
92	1234	1162	228.63
93	1242	1170	186.79
94	1255	1182	61.22
95	1260	1187	242.53
96	1264	1191	27.57
97	1275	1201	54.31
98	1284	1210	70.40
99	1311	1235	234.37
100	1317	1240	3.51
101	1322	1245	561.32
102	1326	1250	299.97
103	1343	1265	348.75
104	1364	1285	106.11
105	1381	1300	85.62
106	1392	1311	58.40
107	1427	1345	137.97
108	1431	1348	328.52
109	1506	1419	7.58
110	1516	1428	61.48
111	1530	1441	29.15
112	1536	1447	26.39
113	1574	1483	262.37
114	1578	1486	148.44
115	1702	1604	9.89
116	1705	1606	31.88
117	1707	1608	17.53
118	1709	1610	13.83
119	1788	1684	251.53
120	1903	1792	742.38
121	1974	1859	207.56
122	3247	3059	0.35
123	3252	3063	1.13
124	3259	3070	0.41
125	3267	3077	2.55
126	3276	3086	3.85
127	3286	3095	2.54
128	3640	3429	101.33
129	3646	3435	87.70

Computational parameters and cartesian coordinates of bis-ketene conformer **2a**-(a,a)

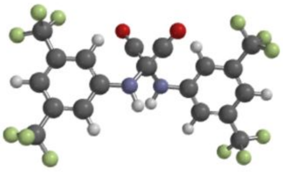

```
SPARTAN '18 Quantum Mechanics Program:  (x86/Darwin)        build  1.4.4

Job type: Geometry optimization.
Method: RWB97X-D
Basis set: 6-31G(D)
Number of basis functions: 556
Number of electrons: 266

SCF model:
 A restricted hybrid HF-DFT SCF calculation will be
 performed using Pulay DIIS + Geometric Direct Minimization


Optimization:
         Step      Energy          Max Grad.      Max Dist.
            1    -2224.315918       0.000367       0.002035
            2    -2224.315918       0.000386       0.002349

<step 2>
Job type: Frequency calculation.
Method: RWB97X-D
Basis set: 6-31G(D)

  Reason for exit: Successful completion
  Quantum Calculation CPU Time :   4:10:51.23
  Quantum Calculation Wall Time:   2:23:41.45


SPARTAN '18 Properties Program:  (x86/Darwin)                  build  1.4.4
  Use of molecular symmetry disabled
```

Cartesian Coordinates (Angstroms)

Atom			X	Y	Z
1	N	N1	-0.2168400	-1.3464181	1.0105009
2	C	C23	-0.9696327	-0.4890812	0.1678639
3	C	C24	-2.2567493	-0.3031859	0.4683866
4	O	O2	-3.3771465	-0.1239766	0.7097840
5	H	H4	0.3441370	-0.8877083	1.7158102
6	C	C2	0.2991739	-2.5510652	0.5343161
7	C	C4	1.3342471	-4.9967723	-0.3854572
8	C	C3	1.3880136	-3.1473003	1.1746452
9	C	C7	-0.2750869	-3.1963041	-0.5667626
10	C	C8	0.2471387	-4.3993288	-1.0161635
11	C	C9	1.8915582	-4.3586783	0.7147725
12	H	H2	1.8405041	-2.6685634	2.0380623
13	H	H1	-1.1362678	-2.7640836	-1.0616202
14	N	N3	0.4604359	1.3839554	-0.8314504
15	C	C1	-0.3966857	0.2627742	-0.9711254
16	C	C11	-0.6405440	-0.1747808	-2.2081591
17	O	O1	-0.8657636	-0.5759068	-3.2731570
18	H	H5	1.4231998	1.1756250	-0.6041352
19	C	C14	-0.0168526	2.5953501	-0.3331759
20	C	C15	-0.9461673	5.0542049	0.6670445
21	C	C16	0.8708799	3.5104657	0.2435267
22	C	C17	-1.3709776	2.9276957	-0.4107658
23	C	C18	-1.8210908	4.1427917	0.0915613
24	C	C19	0.4039193	4.7228241	0.7310345
25	H	H20	1.9271597	3.2698994	0.3181880
26	H	H21	-2.0740068	2.2400023	-0.8669329
27	H	H9	-1.3060117	5.9940732	1.0662492
28	C	C5	-3.2835094	4.4793533	-0.0481452
29	C	C6	1.3854201	5.7170961	1.2960129
30	F	F1	-4.0563043	3.3965216	0.1385825
31	F	F2	-3.6698038	5.4119136	0.8343031
32	F	F3	-3.5573366	4.9514093	-1.2757577

Calculated frequencies of bis of bis-ketene **2a**-(a,a) showing one imaginary frequency.

	Uncorrected cm^{-1}		Corrected Intensity
1	-11	-10	0.03
2	6	6	0.02
3	11	10	0.01
4	14	13	0.26
5	17	16	0.59
6	20	19	0.07
7	22	21	0.03
8	26	25	0.40
9	30	29	0.02
10	57	53	0.01
11	63	60	0.20
12	83	78	0.07
13	88	83	0.18
14	91	86	0.32
15	98	92	0.10
16	118	111	2.28
17	124	117	0.10
18	153	144	0.05
19	154	145	0.70
20	168	158	0.25
21	179	169	0.11
22	201	190	1.84
23	231	218	0.48
24	271	255	1.68
25	278	262	1.86
26	285	269	0.49
27	289	272	2.79
28	296	278	1.41
29	313	295	1.20
30	319	300	1.37
31	322	303	0.08
32	352	332	0.27
33	358	337	0.00
34	368	347	1.21
35	381	359	3.53
36	398	375	4.12
37	418	393	76.30
38	447	421	54.75
39	459	432	0.15
40	465	438	3.25
41	477	449	7.44
42	488	460	21.91
43	499	470	0.98
44	507	478	0.49
45	509	479	0.54
46	534	503	0.26
47	535	504	1.54
48	559	526	41.21
49	571	537	0.82
50	580	547	6.81
51	581	547	1.24
52	600	565	0.27
53	616	581	35.54
54	622	586	0.06
55	646	608	1.43
56	650	612	20.86
57	673	634	51.85
58	674	635	0.40
59	686	646	2.38
60	690	650	14.20
61	692	652	11.25
62	693	653	65.77
63	723	681	17.27
64	727	685	3.02
65	735	692	21.12
66	747	703	1.60
67	747	704	13.08
68	830	781	6.95

69	875	825	3.76
70	885	833	24.35
71	894	842	51.25
72	900	847	47.06
73	907	854	3.56
74	918	865	5.58
75	923	869	5.24
76	924	871	5.86
77	973	916	127.71
78	987	930	0.91
79	1022	963	5.66
80	1023	964	4.29
81	1105	1041	75.27
82	1141	1075	4.34
83	1145	1078	5.36
84	1158	1091	36.35
85	1162	1094	14.70
86	1225	1154	92.37
87	1229	1157	73.84
88	1231	1159	380.20
89	1233	1162	451.84
90	1249	1176	72.07
91	1250	1178	152.92
92	1258	1185	612.82
93	1258	1185	20.49
94	1271	1197	3.65
95	1285	1210	30.90
96	1315	1239	131.15
97	1319	1242	152.13
98	1323	1247	371.28
99	1325	1248	476.49
100	1339	1261	23.36
101	1380	1300	2.95
102	1384	1304	31.06
103	1394	1313	4.75
104	1397	1316	39.81
105	1426	1343	582.03
106	1432	1349	139.07
107	1474	1388	170.58
108	1478	1393	28.08
109	1536	1447	207.95
110	1538	1449	26.16
111	1576	1485	40.38
112	1577	1486	14.08
113	1707	1608	158.65
114	1708	1609	20.43
115	1708	1609	4.74
116	1710	1611	37.93
117	2246	2116	866.43
118	2259	2128	473.15
119	3232	3044	3.20
120	3236	3048	2.97
121	3250	3061	2.01
122	3255	3066	0.99
123	3267	3077	1.83
124	3270	3080	1.92
125	3625	3415	31.56
126	3626	3416	17.12

Computational parameters and cartesian coordinates of bis-ketene conformer **2a**-(s,s)

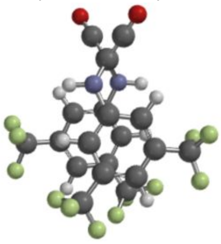

```
SPARTAN '18 Quantum Mechanics Program:  (x86/Darwin)        build  1.4.4

Job type: Geometry optimization.
Method: RWB97X-D
Basis set: 6-31G(D)
Number of basis functions: 556
Number of electrons: 266

SCF model:
 A restricted hybrid HF-DFT SCF calculation will be
 performed using Pulay DIIS + Geometric Direct Minimization


Optimization:
         Step      Energy          Max Grad.       Max Dist.
            1    -2224.331581       0.000664        0.004152
            2    -2224.331581       0.000627        0.002963

<step 2>
Job type: Frequency calculation.
Method: RWB97X-D
Basis set: 6-31G(D)

  Reason for exit: Successful completion
  Quantum Calculation CPU Time :   6:10:56.81
  Quantum Calculation Wall Time:   3:28:07.62


SPARTAN '18 Properties Program:  (x86/Darwin)                build  1.4.4
  Use of molecular symmetry disabled
```

Atom			Cartesian Coordinates (Angstroms) X	Y	Z
1	C	C11	-1.1238490	0.8495250	1.2672791
2	C	C12	-1.6270116	1.3259983	-1.4553809
3	C	C13	-0.3458084	1.7646089	0.5511690
4	C	C14	-2.1688878	0.1968330	0.6167134
5	C	C15	-2.3981334	0.4273742	-0.7352407
6	C	C16	-0.6001610	1.9920053	-0.7919460
7	C	C17	0.7461130	-1.5652399	0.4572459
8	C	C18	2.1935739	-0.0182850	-1.3944166
9	C	C19	1.8205991	-0.7792678	0.8751744
10	C	C20	0.4182735	-1.5898619	-0.9009962
11	C	C21	1.1440182	-0.8305206	-1.8085990
12	C	C22	2.5147576	-0.0029251	-0.0438930
13	H	H31	0.4996694	2.2476997	1.0289821
14	H	H32	-2.7837550	-0.5156621	1.1552981
15	H	H34	2.0927049	-0.7479195	1.9245683
16	H	H35	-0.4350055	-2.1674829	-1.2430234
17	N	N1	-0.0062387	-2.3168860	1.3680009
18	H	H3	-0.8370872	-2.7343232	0.9679149
19	N	N2	-0.8249014	0.5823328	2.6113981
20	H	H6	0.0167155	1.0412813	2.9378238
21	C	C1	-1.0006354	-0.7356801	3.1149529
22	C	C2	-2.0347159	-0.9494553	3.9295921
23	O	O1	-2.9168217	-1.1507284	4.6578020
24	C	C23	-0.1534576	-1.8828658	2.7129526
25	C	C24	0.5636975	-2.5127608	3.6444688
26	O	O2	1.1683864	-3.0608676	4.4705953
27	H	H10	-1.7923853	1.4761751	-2.5140569
28	C	C7	-3.4442946	-0.4083873	-1.4193963
29	C	C8	0.2572132	2.9610843	-1.5621141
30	F	F1	-3.0464426	-1.6993798	-1.4940637
31	F	F2	-3.6996605	0.0024661	-2.6648213
32	F	F3	-4.6027909	-0.4082368	-0.7399889

Calculated frequencies of bis-ketene conformer **2a**-(s,s)

	**Uncorrected cm^{-1}**	**Corrected**	**Intensity**
1	17	16	0.04
2	24	22	0.02
3	27	26	0.10
4	31	29	0.04
5	38	36	0.45
6	41	39	0.77
7	46	43	0.01
8	51	48	0.06
9	55	52	0.18
10	64	61	0.03
11	68	64	0.03
12	89	84	0.49
13	95	90	1.23
14	115	108	0.55
15	121	114	1.71
16	122	115	0.79
17	132	124	0.57
18	157	147	0.45
19	158	149	0.32
20	178	167	0.56
21	199	187	0.13
22	210	198	1.31
23	235	222	0.05
24	266	250	1.87
25	275	259	3.12
26	281	264	0.90
27	294	277	0.64
28	301	284	0.40
29	314	296	0.85
30	321	302	3.48
31	333	314	0.98
32	360	339	0.37
33	364	343	0.34
34	376	354	0.82
35	380	358	1.60
36	403	380	6.39
37	404	381	11.22
38	457	430	5.16
39	469	442	7.53
40	471	443	4.05
41	485	457	3.29
42	507	478	0.66
43	508	479	0.31
44	517	487	12.38
45	524	494	31.76
46	537	506	5.97
47	548	516	9.84
48	560	527	22.64
49	565	532	85.93
50	578	544	4.48
51	579	546	11.79
52	598	564	8.67
53	617	581	17.86
54	620	584	13.85
55	646	609	19.53
56	659	621	35.12
57	672	633	35.42
58	685	646	2.40
59	687	647	7.80
60	689	649	49.83
61	692	652	78.17
62	695	655	11.56
63	721	679	12.95
64	726	684	21.43
65	741	698	6.44
66	751	707	2.51
67	774	730	38.80
68	811	764	19.77

69	874	824	20.91
70	880	829	13.99
71	886	835	77.00
72	895	843	46.50
73	903	851	2.37
74	909	856	1.04
75	914	861	17.14
76	943	888	29.75
77	966	910	13.93
78	988	930	64.21
79	1022	962	2.61
80	1025	966	2.59
81	1096	1032	12.63
82	1137	1071	9.19
83	1140	1074	5.10
84	1141	1075	13.65
85	1155	1088	20.38
86	1198	1129	18.77
87	1202	1132	438.99
88	1215	1145	448.67
89	1227	1156	229.09
90	1238	1166	197.08
91	1253	1181	48.75
92	1258	1185	169.53
93	1261	1188	47.73
94	1270	1197	221.50
95	1282	1207	8.66
96	1306	1230	139.38
97	1315	1238	22.15
98	1318	1241	87.09
99	1323	1247	736.33
100	1333	1256	15.02
101	1379	1299	5.24
102	1381	1301	15.39
103	1385	1304	32.47
104	1403	1322	2.16
105	1428	1345	97.83
106	1438	1355	495.82
107	1471	1386	82.23
108	1477	1391	72.45
109	1537	1448	43.80
110	1541	1451	121.39
111	1574	1482	12.55
112	1576	1485	27.39
113	1706	1607	20.19
114	1706	1607	10.08
115	1710	1611	118.31
116	1711	1612	6.23
117	2242	2112	791.72
118	2265	2133	1016.40
119	3235	3047	1.05
120	3241	3053	0.95
121	3249	3061	1.55
122	3256	3067	1.09
123	3283	3093	2.46
124	3293	3102	3.60
125	3609	3399	18.43
126	3613	3403	19.42
